# The Challenge in Burden of Pulmonary Arterial Hypertension: A Perspective From the Global Burden of Disease Study

**DOI:** 10.1002/mco2.70175

**Published:** 2025-04-24

**Authors:** Yicheng Yang, Zhiwei Zeng, Qiaoxi Yang, Huan Wang, Hanwen Zhang, Wenjie Yan, Peizhi Wang, Chuangshi Wang, Zhanhao Su, Pugazhenthan Thangaraju, Sher Zaman Safi, Beilan Yang, Yaoyao Wang, Jingjing Zhou, Zhiyong Zou, Yuan Huang, Songren Shu, Changming Xiong

**Affiliations:** ^1^ State Key Laboratory of Cardiovascular Disease Fuwai Hospital, National Center for Cardiovascular Diseases, Chinese Academy of Medical Sciences and Peking Union Medical College Beijing China; ^2^ Center of Respiratory and Pulmonary Vascular Disease Fuwai Hospital, National Center for Cardiovascular Disease, Chinese Academy of Medical Sciences and Peking Union Medical College Beijing China; ^3^ Department of Cardiology Anzhen Hospital Beijing China; ^4^ Institute of Child and Adolescent Health School of Public Health National Health Commission Key Laboratory of Reproductive Health, Peking University Haidian District Beijing China; ^5^ Center for Molecular Cardiology University of Zurich Schlieren Zurich Switzerland; ^6^ Medical Research and Biometrics Center National Clinical Research Center for Cardiovascular Diseases Fuwai Hospital, National Center for Cardiovascular Diseases, Peking Union Medical College & Chinese Academy of Medical Sciences Beijing China; ^7^ Department of Cardiovascular Surgery Guangdong Cardiovascular Institute Guangdong Provincial People's Hospital, Guangdong Academy of Medical Sciences Guangzhou China; ^8^ All India Institute of Medical Sciences Raipur India; ^9^ Faculty of Medicine Bioscience & Nursing MAHSA University Selangor Malaysia; ^10^ Echocardiography Medical Center Beijing Anzhen Hospital, Capital Medical University Beijing China; ^11^ Department of Cardiovascular Surgery Fuwai Hospital, National Center for Cardiovascular Diseases, Chinese Academy of Medical Sciences and Peking Union Medical College Beijing China

**Keywords:** pulmonary arterial hypertension, global burden of disease study, disability‐adjusted life years, death, prevalence, pediatric

## Abstract

Pulmonary arterial hypertension (PAH) poses significant clinical management challenges due to gaps in understanding its global epidemiology. We analyzed PAH‐related disability‐adjusted life years (DALYs), deaths, and prevalence from 1990 to 2021. Age‐period‐cohort models and regression analyses assessed temporal trends and projected burdens to 2050. Globally, PAH‐related DALYs declined by 6.6%, but increased by 13.9% in high socio‐demographic index (SDI) countries. Middle SDI regions reported the highest DALYs in 1990 and 2021. Deaths rose by 48.5% worldwide, with high SDI nations experiencing a 76.6% surge. Age‐standardized rates (ASRs) of DALYs and deaths decreased across SDI countries, with high‐middle SDI regions showing the steepest declines. Younger age groups, especially males, had a higher proportion of global DALYs in earlier years, but the burden shifted toward older populations over time, with this trend more pronounced in high‐SDI countries. Age‐period‐cohort analysis revealed declining DALYs in younger ages but rising rates in older cohorts. By 2050, deaths and prevalence are projected to rise, disproportionately affecting females. Significant regional disparities in PAH burden persist, necessitating targeted policies, improved healthcare access, and early detection strategies, especially in underserved areas. Addressing these disparities is critical for mitigating PAH’ s global impact.

## Graphical abstract

**Pulmonary arterial hypertension (PAH) imposes a major global health burden**. In 2021, China mainland reported the highest disability‐adjusted life years (DALYs). Age‐standardized DALYs/death rates inversely correlated with socio‐demographic index (SDI) and universal health coverage index (UHCI), while PAH prevalence increased with these indices. Age‐period‐cohort analysis revealed rising burden with age. Projections predict escalating deaths and prevalence by 2025, demanding targeted interventions.

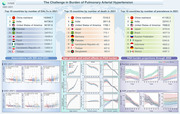

## 1 Introduction

Pulmonary arterial hypertension (PAH) is a progressive and fatal disease characterized by continuously increasing pulmonary vascular pressure and resistance stemming from uncontrolled arteriole remodelling [1]. This disease confounds medical specialists and presents formidable challenges in management [2, 3]. Individuals with PAH face increased mortality risks due to heart hypertrophy and irreversible heart failure without prompt treatment. Despite a moderate improvement in the 5‐year survival rate (from 34 to 60%) following the approval of PAH‐specific drugs [4–3, 6], clinical management and patient survival remain daunting tasks, imposing substantial global health and economic burdens.

With the transformation of lifespan into health span, epidemiological data allow high‐value, patient‐centered, and regionalized disease care. It offers opportunities to enhance access to health services in underserved regions. PAH is a cancer‐like cardiovascular disease that has caused widespread concern [3]. Epidemiological data provide insights into PAH trends and disease burden, enabling timely responses in disease control and treatment. However, the paucity of comprehensive epidemiological evidence significantly hinders a thorough understanding of the PAH landscape and its management.

The Global Burden of Diseases, Injuries, and Risk Factors Study 2021 (GBD 2021) provides the first systematic assessment of PAH epidemiological data. GBD 2021 PAH Collaborators provide a broad overview of the global epidemiology of PAH in terms of prevalence, mortality, and years of life lost, offering a macroscopic demonstration of the disease [7]. However, the lack of a detailed analysis across different regions and countries undermines a deeper understanding of the disease. The GBD Cardiovascular Diseases and Risks Collaborators have provided comprehensive data on cardiovascular diseases, including PAH, across 21 GBD regions, but lacks country‐specific granularity. Additionally, changes in the PAH burden over the past several decades have not been thoroughly discussed [8]. Dividing the world into five socio‐demographic index (SDI) categories provides a valuable advantage in analyzing disease burden, as SDI levels reflect key socio‐economic factors, healthcare access, and infrastructure. This categorization facilitates more meaningful comparisons between countries with comparable socio‐economic profiles, potentially revealing disease burden patterns that traditional geographic analyses might overlook. It also highlights disparities in disease prevention, diagnosis, and treatment across the SDI spectrum, thereby informing targeted public health strategies. However, the epidemiological characteristics and trends of PAH across different SDI regions remain inadequately characterized, highlighting the need for further research. Therefore, this study aims to comprehensively elucidate the specific PAH burden including disability‐adjusted life years (DALYs), deaths, and prevalence using GBD 2021 data, highlighting epidemiological characteristics and trends in age‐ and sex‐related discrepancies at global, different SDI regions and country levels. Pediatric PAH can be diagnosed in children of all ages, posing significant health threats to children and contributing to premature deaths, thereby impeding societal progress [9]. A comprehensive understanding of the epidemiological characteristics of pediatric PAH patients is still lacking. Here, we also analyzed age‐ and sex‐stratified epidemiological patterns among PAH patients under 19 years old.

The age‐period‐cohort (APC) model examined the effects of age, period, and birth cohort on trends in PAH burden, addressing collinearity among age, period, and cohort, offering a distinct advantage over conventional epidemiological analyses [10]. Therefore, we constructed an APC model to analyze the temporal trends, dissecting the specific impacts of age, period, and birth cohort to reveal the actual epidemiological characteristics in countries with different SDI levels. In addition, projected global trends of DALYs, deaths, and prevalence of PAH through 2050 were also analyzed in this study to properly evaluate the burdens of the disease and its long‐term outcomes.

## 2 Results

### 2.1 Global and Regional Trends in PAH From 1990 to 2021

Globally, the numbers of DALYs in 1990 and 2021 were 687,419.3 (95% uncertainty interval [UI]: 535,240.8–813,086.3) and 642,104.3 (95% UI: 552,272.7–728,993.2) with a decrease of 6.6% during the past 30 years, while a 13.9% increase was exhibited in high SDI countries. The middle SDI regions recorded the highest cases of DALYs in both 1990 and 2021, as well as the greatest number of PAH cases. The ASRs for DALYs globally were 13.2 out of 100,000 in 1990 and 8.2 out of 100,000 in 2021, indicating a 37.9% reduction. A decrease in DALY ASR was observed across all five SDI regions over the past few decades, with the high‐middle SDI showing the largest significant decrease of 50.4% and 2.20 in the estimated annual percent change (EAPC) (Table 1).

**TABLE 1:** Disability adjusted life years (DALYs) and age‐standardized rates for pulmonary arterial hypertension in 1990 and 2021 and estimated overall and annual percentage changes from 1990 to 2021 by five SDI quantiles.

Number				Age‐standardized rate			
Location	1990 (95% UI)	2021 (95% UI)	Percent change (95% UI)	1990 per 100, 000 (95% UI)	2021 per 100, 000 (95% UI)	Percent change (95% UI)	EAPC (95% CI)
Global	687,419.3 (535,240.8, 813,086.3)	642,104.3 (552,272.7, 728,993.2)	−0.07 (−0.27, 0.18)	13.2 (10.8, 15.4)	8.2 (7.1, 9.4)	−0.38 (−0.48, −0.25)	−1.31 (−1.43, −1.19)
SDI							
High SDI	81,792.0 (77,184.7, 88,110.3)	93,181.6 (84,873.4, 99,191.5)	0.14 (0.03, 0.24)	9.2 (8.7, 9.9)	6.2 (5.8, 6.5)	−0.33 (−0.40, −0.27)	−1.39 (−1.57, −1.22)
High‐middle SDI	127,637.6 (106,711.1, 154,437.7)	99,448.2 (85,757.4, 117,639.4)	−0.22 (−0.40, 0.02)	13.1 (10.9, 16.0)	6.5 (5.6, 7.9)	−0.51 (−0.62, −0.35)	−2.20 (−2.36, −2.05)
Middle SDI	210,945.6 (172,856.8, 258,349.1)	197,170.7 (148,781.5, 232,321.2)	−0.07 (−0.35, 0.23)	14.3 (11.7, 17.7)	8.2 (6.3, 9.7)	−0.42 (−0.58, −0.25)	−1.40 (−1.61, −1.18)
Low‐middle SDI	195,280.8 (117,185.1, 245,590.7)	156,400.3 (122,426.3, 194,166.5)	−0.20 (−0.39, 0.20)	14.9 (10.7, 18.4)	9.1 (7.1, 11.6)	−0.39 (−0.51, −0.21)	−1.33 (−1.43, −1.24)
Low SDI	71,124.9 (48,628.4, 111,613.7)	95,341.8 (67,470.9, 133,050.0)	0.34 (−0.03, 0.81)	12.4 (7.8, 19.2)	9.3 (6.1, 13.2)	−0.25 (−0.43, −0.07)	−0.78 (−0.85, −0.71)

*Abbreviations*: SDI, sociodemographic index; EAPC, estimated annual percent change.

Global PAH deaths consistently increased from 14,842.5 in 1990 to 22,020.5 in 2021. During the past 30 years, almost all SDI countries were on an upward trend, especially in the high SDI regions with a 76.6% increase. ASR of deaths decreased globally from 3.5 cases/million in 1990 to 2.7 cases/million in 2021 (a 22.9% and a 0.57 EAPC decrease), with significant declines in high‐middle SDI countries (a 31.4% and a 1.07 EAPC decrease) (Table 2).

**TABLE 2:** Deaths and age‐standardized rates for pulmonary arterial hypertension in 1990 and 2021 and estimated overall and annual percentage changes from 1990 to 2021 by five SDI quantiles.

Number				Age‐standardized Rate			
Location	1990 (95% UI)	2021 (95% UI)	Percent change (95% UI)	1990 per 100, 000 (95% UI)	2021 per 100, 000 (95% UI)	Percent change (95% UI)	EAPC (95% CI)
Global	14,842.5 (12,369.9, 17,484.9)	22,020.5 (18,239.2, 25,351.6)	0.48 (0.21, 0.78)	0.4 (0.3, 0.4)	0.3 (0.2, 0.3)	−0.22 (−0.35, −0.08)	−0.57 (−0.72, −0.42)
SDI							
High SDI	2616.7 (2379.3, 2850.0)	4620.8 (3919.1, 5054.5)	0.77 (0.55, 0.97)	0.3 (0.2, 0.3)	0.2 (0.2, 0.2)	−0.17 (−0.25, −0.08)	−0.56 (−0.74, −0.38)
High‐middle SDI	3213.8 (2771.6, 3908.4)	4326.3 (3594.3, 5141.3)	0.35 (0.08, 0.70)	0.4 (0.3, 0.4)	0.2 (0.2, 0.3)	−0.32 (−0.45, −0.14)	−1.07 (−1.27, −0.87)
Middle SDI	4728.5 (3774.3, 5852.1)	7548.4 (5141.3, 9025.5)	0.60 (0.12, 1.14)	0.5 (0.4, 0.6)	0.3 (0.2, 0.4)	−0.28 (−0.50, −0.02)	−0.63 (−0.88, −0.37)
Low‐middle SDI	3124.6 (2219.6, 3904.4)	3727.7 (2756.5, 5091.4)	0.19 (−0.09, 0.62)	0.3 (0.2, 0.5)	0.3 (0.2, 0.4)	−0.25 (−0.39, −0.08)	−0.71 (−0.79, −0.63)
Low SDI	1145.0 (740.5, 1754.9)	1781.8 (1146.6, 2544)	0.56 (0.12, 0.97)	0.3 (0.2, 0.5)	0.3 (0.1, 0.4)	−0.16 (−0.34, 0.10)	−0.33 (−0.44, −0.22)

*Abbreviations*: SDI, sociodemographic index; EAPC, estimated annual percent change.

The prevalence of total PAH cases nearly doubled between 1990 and 2021, rising from an estimated 105,703.3 cases (95% UI: 86,381.4–130,334.3) to 191,808.2 cases (95% UI: 155,356.9–235,787.1). This increase was observed across all five SDI regions, with middle SDI countries accounting for the largest number of patients in both 1990 and 2021. Notably, the low SDI regions experienced the greatest percentage increase, with a 1.18‐fold rise. The global ASR of PAH prevalence has remained relatively stable at 22.8 cases/million, and the increase in ASR of prevalence was more pronounced in middle and low‐middle SDI quantiles, with percentage changes of 7.0% and EAPCs of 0.21 and 0.27, respectively (Table 3).

**TABLE 3:** Prevalence cases and age‐standardized rates for pulmonary arterial hypertension in 1990 and 2021 and estimated overall and annual percentage changes from 1990 to 2021 by five SDI quantiles.

Number				Age‐standardized rate			
Location	1990 (95% UI)	2021 (95% UI)	Percent change (95% UI)	1990 per 100, 000 (95% UI)	2021 per 100, 000 (95% UI)	Percent change (95% UI)	EAPC (95% CI)
Global	105,703.3 (86,381.4, 130,334.3)	191,808.2 (155,356.9, 235,787.1)	0.81 (0.73, 0.89)	2.3 (1.9, 2.8)	2.3 (1.8, 2.8)	−0.01 (−0.02, 0.00)	0.03 (0.01, 0.06)
SDI							
High SDI	27,053.8 (21,977.2, 33,298.4)	41,452.3 (33,445.0, 51,587.9)	0.53 (0.46, 0.60)	2.7 (2.2, 3.3)	2.6 (2.2, 3.2)	−0.01 (−0.02, 0.00)	−0.02 (−0.05, 0.01)
High‐middle SDI	27,439.2 (22,296.2, 33,800.5)	42,635.9 (34,236.2, 52,931.7)	0.55 (0.47, 0.63)	2.6 (2.1, 3.2)	2.5 (2.1, 3.1)	−0.03 (−0.04, −0.02)	0.11 (0.01, 0.21)
Middle SDI	29,051.3 (23,769.5, 35,768.6)	59,666.7 (48,064.6, 73,648.4)	1.05 (0.90, 1.20)	2.1 (1.7, 2.5)	2.2 (1.8, 2.7)	0.07 (0.06, 0.08)	0.21 (0.14, 0.28)
Low‐middle SDI	15,078.7 (12,416.6, 18,609.1)	32,714.8 (26,546.4, 40,620.3)	1.17 (1.09, 1.24)	1.8 (1.4, 2.2)	1.9 (1.5, 2.3)	0.07 (0.06, 0.09)	0.27 (0.24, 0.29)
Low SDI	6965.7 (5737.0, 8608.7)	15,180.8 (12,474.4, 18,747.1)	1.18 (1.14, 1.22)	2.1 (1.7, 2.5)	1.9 (1.6, 2.4)	−0.06 (−0.08, −0.05)	−0.21 (−0.29, −0.13)

*Abbreviations*: SDI, sociodemographic index; EAPC, estimated annual percent change; CI, confidence interval; UI, uncertainty interval.

### 2.2 National Trend of PAH Burden From 1990 to 2021

Tables S3–S5 provide the DALYs, deaths, and prevalence data for each country and region. In 2021, China mainland, India, and the United States remained the top three countries in terms of DALY numbers, with 150,940.7 (99,583.3–186,503.4), 94,736.7 (69,016.9–131,902.1), and 36,197.6 (33,003.8–38,647.8) cases, respectively. From 1990 to 2021, the number of DALYs in five countries or regions, including Taiwan (Province of China), Qatar, Mauritius, Afghanistan, and Kuwait, more than doubled. Taiwan (Province of China) and Qatar ranked first and second, with percentage changes of 273.9% and 268.7%, respectively. Puerto Rico, Greenland, and Egypt were the top three regions with the largest reductions in DALYs, showing decreases of 81.0, 76.4, and 69.8% over the past three decades. Regarding ASR, Egypt (74.2 out of 100,000 in 1990 vs. 16.7 out of 100,000 in 2021) and Mongolia (50.7 out of 100,000 in 1990 vs. 43.9 out of 100,000 in 2021) were the highest‐ranking countries in 1990 and 2021. Puerto Rico, Egypt, and Greenland exhibited a significant decrease in DALY ASR, with reductions of 83.2, 77.8, and 77.6%. While the ASR of DALYs decreased in most countries, six nations and territories showed the opposite trend: Mauritius, Latvia, Georgia, Guyana, Taiwan (Province of China), and Kuwait (in descending order of growth change). Notably, the SDI in these countries was greater than or equal to the medium SDI level (Table S3).

In terms of the number of deaths, similarly, China mainland, India, and the United States of America were the top three countries in both 1990 and 2021, with 7318.0 (4835.7–9075.8), 2611.6 (1738.6–4059.8), and 1785.1 (1535.4–1944.9) deaths, respectively, in 2021. From 1990 to 2021, Puerto Rico experienced the most significant decrease in the number of deaths. Notably, of the top 10 countries with the largest increases in deaths, 80% were high‐ or high‐middle‐SDI countries. Taiwan (Province of China) and Latvia, both with high SDI levels, recorded the largest increases in the number of deaths, with increases of 510.0 and 486.4%, respectively. After age standardization, Cyprus ranked first in 1990 with 20.9 cases per million, while Mongolia ranked first in 2021 with 15.9 cases per million. Similar to the DALY ASR, Puerto Rico, Egypt, and Greenland also saw a significant decrease in death ASR, with reductions of 70.9, 63.7, and 61.2%, respectively. While age‐standardized mortality declined in most countries, ASR increased in nine countries or regions, including Latvia, the Republic of Moldova, Georgia, Mauritius, Guyana, Lithuania, Taiwan (Province of China), Kuwait, and Estonia (in descending order of positive percentage change), most of which were high‐SDI areas (Table S4).

The number of prevalent cases increased in nearly all countries over the past three decades. In 1990 and 2021, China mainland led with 22,027.9 (17,840.2–27,321.5) and 41,135.2 (32,838.9–51,357.3) cases, respectively, followed by India with 10,592.0 (8,714.5–13,169.4) and 23,741.7 (19,176.4–29,559.1) cases, respectively. Among the top 10 countries in 1990, the United States of America, Japan, Brazil, and Indonesia saw increased rankings in 2021, whereas Russia, Germany, Italy, and France declined. From 1990 to 2021, Qatar experienced the greatest percentage increase in cases of 7.17, followed by the United Arab Emirates of 6.16 and Jordan of 3.54. Countries with low or low‐middle SDI levels comprised more than half of the top 30 countries with increased prevalence. Bulgaria had the most significant reduction, with a 29.1% decrease in prevalence number. ASR data showed that Switzerland consistently ranked first, with 67.6 cases/million in 1990 and 70.9 cases/million in 2021, followed by Sweden, with 60.5–63.0 cases/million. In 1990, the top 20 countries by ASR were predominantly high or high‐middle SDI, with exceptions such as Ghana, a low‐middle SDI country. In 2021, high‐SDI countries dominated the top 20. Nigeria had the most notable increase in ASR of 52.9%, followed by El Salvador of 46.7% and Bangladesh of 42.1%. Among the top 10 countries with the highest ASR increases, seven were from low‐middle SDI regions, two were high SDI countries, including Denmark and Germany, and one had middle SDI level (Türkiye) (Table S5).

### 2.3 Age and Sex Differences in PAH from 1990 to 2021

Based on the established age division of GBD, individuals with PAH were divided into four age groups: younger than 14 years, 14–49 years, 50–69 years, and older than 70 years.

**FIGURE 1:**
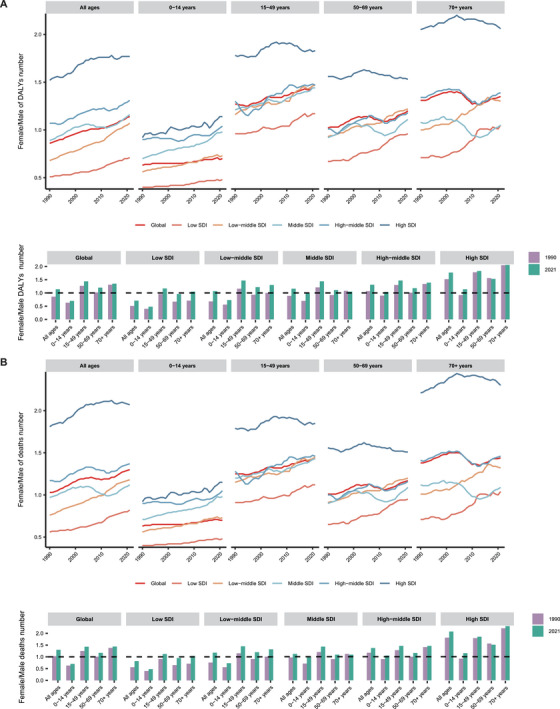
DALYs and deaths shifts across age and sex difference. (A) The female/male proportion of DALYs number among different age groups including 0–14 years, 15–49 years, 50–69 years, and more than 70 years groups from 1990 to 2021. (B) The female/male proportion of death number among different age groups including 0–14 years, 15–49 years, 50–69 years, and more than 70 years groups from 1990 to 2021. *Abbreviations*: DALYs, disability‐adjusted life years; SDI, socio‐demographic index.

Regarding the global DALYs number, younger age groups (0–14 and 15–49 years) predominated in earlier years. Notably, the number of DALYs in younger populations steadily declined over time, while the burden in older age groups, particularly those over 70 years, continued to rise. Similar trends were observed across different SDI countries, with higher proportions of DALYs attributed to older populations in countries with higher SDI. This trend was most pronounced in middle‐high and high SDI nations. In contrast, low and lower‐middle SDI countries still saw a high proportion of DALYs in younger populations, indicating a significant difference in the age distribution of DALYs between higher and lower SDI regions. A similar trend was observed in both males and females. Compared with females, the proportion of DALYs number among males was higher in younger individuals across different SDI countries, especially in children aged 0–14 years (Figures 1A and S3A and Tables S6 and S7).

Over the past 30 years, the number of deaths among children aged 0–14 years has gradually declined, while the number of elderly individuals aged over 70 years has continued to rise. This trend is more pronounced in countries with middle, high‐middle, and high SDI. In terms of gender differences, the proportion of child deaths in the male population remains higher than in females, especially in those low and low‐middle SDI regions (Figures 1B and S3B and Tables S6 and S7).

Globally, the highest prevalence was observed in the 15–49 and 50–69 years age groups. In countries with higher SDI, particularly in high‐SDI countries, individuals aged 70+ years constituted a greater proportion, whereas younger age groups (0–14 and 15–49 years) were more prevalent in lower‐SDI countries. Females accounted for 62.71% of the global prevalence and there was a pronounced increase in older male populations (aged 50–69 and 70+ years) in high‐middle‐ and high‐SDI countries during the past decades (Figures S2 and S3C and Tables S6 and S7).

### 2.4 The Trends in Pediatric PAH from 1990 to 2021

The presentation of epidemiological data on pediatric PAH is crucial for enhancing disease management and control. The data across age and sex groups from 1990 to 2021 in the population aged 0–19 years were analyzed. We categorized individuals under 19 years of age into six age groups—less than 12 months, 12–23 months, 2–4 years, 5–9 years, 10–14 years, and 15–19 years—based on GBD age divisions.

**FIGURE 2:**
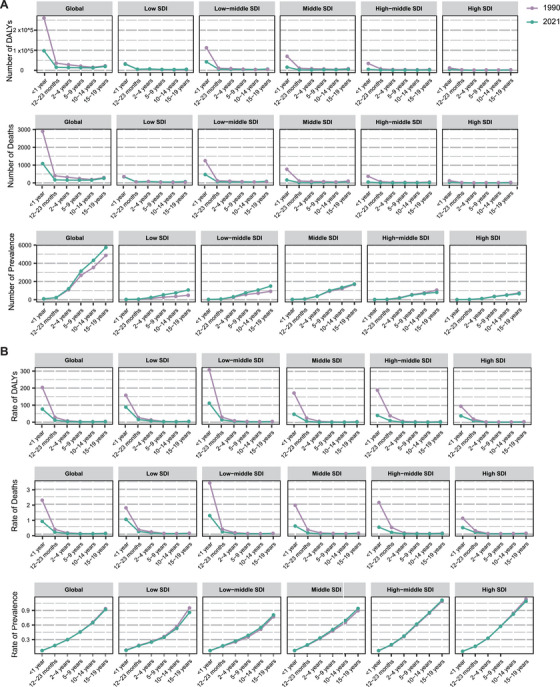
The trends of pediatric PAH by age and SDI group. (A) The number of DALYs, deaths, and prevalence in 1990 and 2021 among different age groups, including the less than 12 months, 12–23 months, 2–4 years, 5–9 years, 10–14 years, and 15–19 years age groups. (B) The rates of DALYs, deaths, and prevalence in 1990 and 2021 among different age groups, including the less than 12 months, 12–23 months, 2–4 years, 5–9 years, 10–14 years, and 15–19 years age groups. The dotted line represents the 50% confidence interval. *Abbreviations*: DALYs, disability‐adjusted life years; PAH, pulmonary arterial hypertension; SDI, socio‐demographic index.

Globally, the highest numbers of DALYs and deaths occurred in children under 12 months of age, followed by a significant decline after the first year. Over the past three decades, the numbers of DALYs and deaths in children under 12 months of age have decreased by 62.6% globally, with the most notable reductions seen specifically in low‐middle and middle SDI countries. The number of DALYs for children aged 10–19 years has remained nearly unchanged from 1990 to 2021. The ASR of DALYs and deaths followed a similar pattern, peaking in children under 1 year of age. From 1990 to 2021, the ASR of DALYs and deaths in children under 1 year significantly declined across all five SDI country groups. Nonetheless, low and low‐middle SDI regions continue to exhibit higher ASRs of DALYs and deaths compared with higher SDI regions, which requires further attention (Figure 2A,B and Tables S8 and S9). Among PAH patients under 10 years of age, males demonstrated higher ASRs of DALYs and deaths, especially in low‐SDI countries, with this trend reversing as age increased. In the 10–19 years age group, females accounted for a higher proportion of DALYs and deaths, showing a significant upward trend over the past three decades. Globally, compared with 1990, the proportion of DALYs and deaths among female children aged 1–4 years declined in 2021 (Figure S4A,B).

In 2021, the prevalence of PAH globally increased with age. For the 10–14 and 15–19 years age groups, prevalence rose by 22.1 and 17.8%, respectively, compared with 1990. Trends in prevalence across different age groups in 1990 and 2021 were generally consistent with the trends in absolute numbers. The ASR of prevalence in children under 5 years old was similar across all five SDI groups. However, in older age groups, higher ASRs were observed in high‐middle and high‐SDI countries (Figure 2A,B and Tables S8 and S9). PAH was more prevalent among females under 19 years of age, accounting for over 50% of cases, particularly in low‐SDI regions. The sex distribution of PAH prevalence did not change significantly from 1990 to 2021 (Figure S4C).

### 2.5 Associations Between PAH Burden with SDI and Universal Health Coverage Index Levels

**FIGURE 3:**
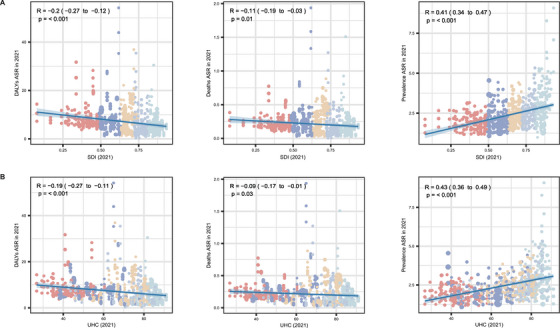
Associations of the SDI (A) and UHCI (B) with age‐standardized rates of DALYs, deaths, and prevalence from PAH in 2021. Pearson's correlation test was used to evaluate the associations between the SDI and UHCI and age‐standardized rates. *Abbreviations*: PAH, pulmonary arterial hypertension; SDI, socio‐demographic index; UHCI, universal health coverage index; CIs, confidence intervals.

Figure 3 presents the associations of SDI and universal health coverage index (UHCI) in 2021 with the ASRs of DALYs, deaths, and prevalence. In summary, all ASRs were significantly correlated with both SDI and UHCI levels. As SDI levels improved, the ASR of DALYs (*r* = −0.20 [−0.27, −0.12], *p* < 0.001) and deaths (*r* = −0.11 [−0.19, −0.03], *p *= 0.010) decreased. In contrast, SDI was positively correlated with the prevalence ASR (*r* = 0.41 [95% CI: 0.34–0.47], *p* < 0.001), indicating higher PAH prevalence in countries with higher SDI levels. Similarly, a higher UHCI was associated with a lower ASR of DALYs (*r* = −0.19 [−0.27, −0.11], *p* < 0.001) and deaths (*r* = −0.09 [−0.17, −0.01], *p* = 0.030), but a higher ASR of prevalence (*r* = 0.43 [0.36–0.49], *p* < 0.001).

### 2.6 Age, Period, and Cohort Effects on PAH Burden in Different SDI Regions

**FIGURE 4:**
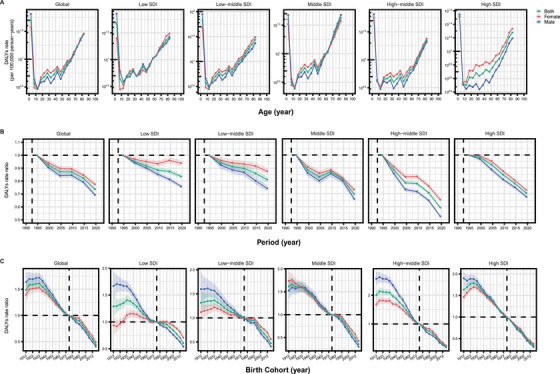
Age‐period‐cohort models of PAH DALYs in global and five SDI quantiles. (A) Age effects show the fitted longitudinal age curves of PAH DALYs rate (per 100,000 person‐years) and the corresponding 95% CIs. (B) Period effects show the relative risk of PAH DALYs rate of each period compared with the reference (period 1992–1996) adjusted for age and nonlinear cohort effects and the corresponding 95% CIs. (C) Cohort effects show the relative risk of PAH DALYs rate of each cohort compared with the reference (cohort 1921–1929) adjusted for age and nonlinear period effects and the corresponding 95% CIs. *Abbreviations*: PAH, pulmonary arterial hypertension; DALYs, disability‐adjusted life years; SDI, socio‐demographic index; CIs, confidence intervals.

The APC model was employed to reveal the actual burden of PAH, independent of the specific impacts of age, period, and birth cohort. Figure 4A–C presents APC model‐derived estimates of PAH DALYs by SDI quantiles. Globally and across all SDI quantiles, DALYs in the 0–5 years age group showed a significant decline. As age increased, DALYs gradually rose, particularly among individuals over 50 years old, with middle SDI countries experiencing the most pronounced increase. In high SDI countries, a gender disparity in DALYs was evident, with female DALYs levels exceeding those of males. However, as age progressed, the DALYs for male patients rose sharply, approaching those of female patients and, in some cases, surpassing those of female patients, particularly in middle SDI countries. Period effects revealed a decreasing trend in DALYs rates among both males and females globally and across all five SDI quintiles. Notably, the decline in DALYs was more pronounced in high‐middle SDI countries, and across all regions, the reduction in DALYs over the past 30 years was greater for male PAH patients than for females. Cohort effects indicated that patients born between 1912 and 1932 experienced an increase in DALYs, which was more pronounced in males than in females. In contrast, DALYs rates improved significantly for those born after 1932, particularly in high SDI countries. Moreover, the APC results for death show a similar trend to those observed for DALYs (Figure S5).

PAH prevalence rates increased with age, except in the 80–84 years age group. Females consistently showed higher prevalence rates than males across all age groups. Period effects showed an increasing prevalence rate globally in males but not in females. Moreover, period effects varied among the SDI quintiles, with a continuously increasing prevalence rate in the past 30 years in low‐middle and middle SDI quintiles. Cohort effects were less pronounced globally, with trends varying across SDI regions as indicated by improvements in low‐SDI regions and deterioration in low‐middle, middle, and high‐middle‐SDI regions (Figure S6).

### 2.7 Age, Period, and Cohort Effects on PAH Burden in Exemplary Countries

We illustrated the unfavorable trends of the disease in exemplary countries across the SDI quintiles to further reveal the changes of PAH during the past three decades, which provided more reliable support for policy formulation and implementation in regions and countries.

**FIGURE 5:**
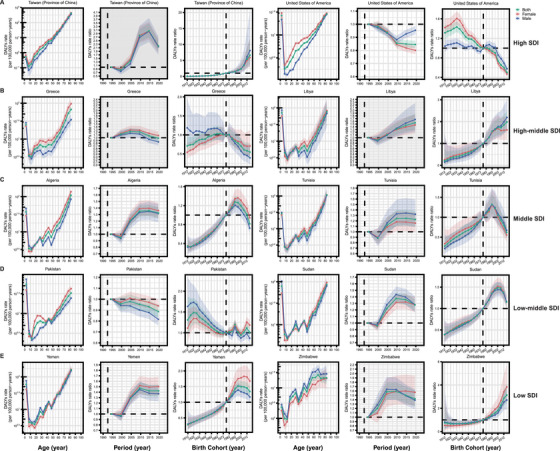
Exemplar countries across different regions and SDI quantiles showing unfavorable age‐period‐cohort effects in PAH DALYs. Ten countries or regions including (A) two high‐SDI regions [Taiwan (Province of China) and the United States of America], (B) two high‐middle‐SDI countries (Greece and Libya), (C) two middle‐SDI countries (Algeria and Tunisia), (D) two low‐middle‐SDI countries (Pakistan and Sudan), and (E) two low‐SDI countries (Yemen and Zimbabwe) were selected, which presented the unsatisfactory APC effects in DALYs. Overall trends in the DALYs rate from 1990 to 2021. The fitted longitudinal age curves display the DALYs rate of PAH per 100,000 person‐years, along with the corresponding 95% CIs. Period effects illustrate the relative risk of PAH DALYs for each period, compared with the reference period of 1992–1996, adjusted for age and nonlinear cohort effects, and include the corresponding 95% CIs. Cohort effects show the relative risk of PAH DALYs for each cohort, using the 1921–1929 cohort as the reference, adjusted for age and nonlinear period effects, with the corresponding 95% CIs provided. *Abbreviations*: PAH, pulmonary arterial hypertension; DALYs, disability‐adjusted life years; SDI, socio‐demographic index; CIs, confidence intervals.

Despite a global downward trend in DALYs and deaths, some countries have yet to witness notable improvements, and in some cases, the situation has actually worsened. Ten countries or regions including two high‐SDI regions [Taiwan (Province of China) and the United States of America], two high‐middle‐SDI countries (Greece and Libya), two middle‐SDI countries (Algeria and Tunisia), two low‐middle‐SDI countries (Pakistan and Sudan), and two low‐SDI countries (Yemen and Zimbabwe) were selected, which presented the unsatisfactory APC effects in DALYs (Figure 5A–E). Similarly, across these representative nations, there was a marked reduction in DALYs among children under 10 years of age, which then rose significantly with advancing age. In addition, gender disparities have been noted in certain countries. Period effects have shown an increasing trend in DALYs rates over time in Taiwan (Province of China), Greece, Libya, Algeria, Tunisia, Sudan, Yemen, and Zimbabwe. On the other hand, despite a slight downward trend in the United States and Pakistan, it was not significant, and even exhibited a contrasting pattern in some instances. Cohort effects suggested that, in most of the exemplary countries including Taiwan (Province of China), Libya, Algeria, Tunisia, Sudan, Yemen, and Zimbabwe, patients born after 1997 even exhibited a trend of increasing DALYs. Although a few selected countries exhibited a declining trend in DALYs for later birth cohorts, the decrease was not very significant.

We also selected ten countries including two high‐SDI regions (Germany and the United States of America), two high‐middle‐SDI countries (Italy and Spain), two middle‐SDI countries (Algeria and Sri Lanka), two low‐middle‐SDI countries (Morocco and Sudan), and two low‐SDI countries (Afghanistan and Yemen) to exhibit the unsatisfactory APC effects in deaths (Figure S7A–E). Similar to DALYs, deaths showed a notable decline among children under 10 years old, followed by a significant increase with age. Moreover, gender differences were observed in some countries. Both period and cohort effects suggested that, in most representative countries, there had been no significant improvement in mortality over time or across cohorts, particularly in countries with SDI lower than high‐middle.

The APC effects of prevalence in exemplary countries are illustrated in Figure S8A–E. We analyzed ten countries representing various SDI levels: two high‐SDI countries (France and Germany), two high‐middle‐SDI countries (Italy and the Russian Federation), two middle‐SDI countries (China mainland and Türkiye), two low‐middle‐SDI countries (Nigeria and Bangladesh), and two low‐SDI countries (Chad and Afghanistan). In all cases, prevalence rose sharply with age. Period and cohort effects generally showed an upward trend across most countries. Moreover, notable sex differences were observed, especially emphasized by age effects.

### 2.8 PAH Burden Projections Through 2050 Across Different Sex Populations

Global projections were needed to properly evaluate the burdens of PAH and its long‐term outcomes. After demonstrating the historical trends of the PAH burden globally, we conducted a regression model to further forecast the cases and ASRs of DALYs, deaths, and prevalence through 2050 (Table S10).

A declining trend in DALYs was observed globally, primarily among the male population. The global DALY cases were projected to reach 519,398.8 (412,506.0–626,291.6), reflecting a 19.11% decrease compared with 2021. In contrast, the number of deaths was expected to gradually increase, with a significant rise among females. By 2050, global PAH‐related deaths were projected to reach 36,594.8 (32,487.7–40,701.9), marking an increase of nearly 70% from 2021. The prevalence cases were projected to grow rapidly over the next 30 years for both males and females, with females consistently representing the majority across all time points. By 2050, the number of cases was expected to reach 300,484.7 (240,568.8–360,400.6), nearly doubling the count from 2021 (Figure S9A).

The ASR of DALYs and deaths were projected to decline over the next 30 years, particularly among male patients. In contrast, the ASR of prevalence is expected to remain stable throughout the projected period, with a slight upward trend observed in the male population (Figure S9B).

## 3 Discussion

PAH was first included in the most updated GBD 2021 data, and we provide the comprehensive epidemiological characteristics of PAH at global, different SDI regions, and country levels. Key findings include the following: (i) Globally, DALYs numbers have decreased by 6.6%, and ASRs have dropped by 37.9% over the past 30 years. The middle‐SDI regions bear the highest burden of PAH, exhibiting the greatest numbers in DALYs, deaths, and prevalence. (ii) In 2021, China mainland and India had the highest numbers of DALYs, deaths, and prevalence, accounting for 38.3, 45.1, and 33.8% of the global total, respectively. (iii) Over the past few decades, the burden of PAH has shifted from younger to older age groups, with a particularly pronounced rise in those over 70 years in higher SDI countries, while lower SDI countries still experience a higher proportion of DALYs in younger populations, especially among males. (iv) The burden of pediatric PAH, marked by the highest DALYs and deaths in children under 12 months of age, has decreased globally, especially in lower SDI countries, but the prevalence and sex distribution of the disease show notable trends, with higher rates in older age groups and more cases in females, particularly in low‐SDI regions. (v) Higher SDI and UHCI are associated with lower ASRs of DALYs and deaths but higher ASRs of PAH prevalence. (vi) The APC model reveals that while DALYs and deaths for PAH have decreased globally, particularly in high‐SDI countries, prevalence increased with age, especially among females, with varying trends across SDI regions and birth cohorts.

PAH is a severe and terrifying disease that diminishes the quality of life and can lead to death, imposing a considerable burden on families, society, and countries [11, 12, 13]. Currently, the complexities of PAH treatment remain unsolved. The pathogenesis involves numerous factors, such as gene mutation, metabolic reprogramming [14], congenital heart diseases (CHDs) [15], connective tissue diseases [16], and even the dysfunction of the gut microbiota [17]. Early identification and understanding of epidemiological characteristics are crucial, as accurate estimates of PAH DALYs, deaths, and prevalence remain elusive. Over the past 30 years, significant declines in ASRs of DALYs and death were observed globally, suggesting an effective PAH control, driven by improvements in disease awareness, early diagnosis, and advancements in treatment options. In the late 19th century, Ernst von Romberg, a famous German physician and scientist, observed the abnormalities in the pulmonary blood vessels during autopsy and was the first to designate the condition as “pulmonary vascular sclerosis” [18], which opened the gradual understanding of pulmonary hypertension. Unlike other cardiovascular diseases, PAH has only been recognized for a little over a century. Due to its rapid progression and the limited understanding of the condition, patients often faced extremely high rates of disability and mortality [19]. In the 1990s, the emergence of the first targeted PAH treatment, prostacyclin, followed by other targeted therapies such as endothelin receptor antagonists and phosphodiesterase inhibitors, significantly improved the quality of life and prognosis for patients [1, 20]. The reduction in ASRs of DALYs and deaths reflected the success of healthcare systems while a variation across different SDI regions should be noted. Middle, low‐middle and low‐SDI regions, while seeing a decline in DALYs and deaths, still carry the highest rates due to limited access to quality healthcare, lower awareness, and inadequate management strategies, highlighting the need for region‐specific health interventions and policies.

While PAHs predominantly affect the young‐to‐middle‐aged population globally, older age groups should not be neglected, especially in high‐SDI countries. The aging global population has led to a marked change in the age distribution of patients with PAH [21]. Evidence from the United States of America and Europe showed that PAH was frequently diagnosed in older patients who often presented with cardiovascular comorbidities [22]. Over the past 30 years, one of the most notable changes in PAH epidemiology has been the increasing diagnosis of idiopathic PAH in older populations, which has become the most common subtype in the Western world [23, 24, 25]. Further exploration is needed to understand the underlying reasons for this age shift. From 1990 to 2021, early recognition, advanced disease management, and extended life expectancy have contributed to the diagnosis of more elderly patients with PAH in countries with high disease awareness and response. However, it remains unclear whether the age shift reflects an intrinsic change in the PAH phenotype or improved detection in older adults [22]. Nevertheless, the elderly population with PAH, who are often burdened with comorbidities, face more severe conditions and poorer prognoses, necessitating greater attention and intervention. In contrast, low‐SDI countries may not show a noticeable age shift in PAH demographics, likely due to a larger proportion of patients with PAH linked to CHD and challenges in community awareness and disease management. Nevertheless, further investigation is needed to determine the most appropriate treatment strategy and the cost‐effectiveness for elderly patients. Over the past three decades, PAH has remained the predominant disease affecting females, while the proportion of older males in high‐SDI countries has increased. Male populations with PAH, though less common, face a greater risk of poor prognosis than women, a phenomenon known as the “estrogen paradox” in PAH [26, 27]. This highlights the need for increased interventions and higher healthcare expenditures for male patients with PAH, especially in countries with higher SDI.

Pediatric PAH, a rare disease affecting neonates, infants, and children, leads to significant morbidity and mortality and imposes a substantial disease burden. Managing pediatric PAH is challenging due to insufficient data on its natural history, disease mechanisms, and treatment responses [9, 28]. The burden of pediatric PAH remains undefined, and we outlined the epidemiological landscape of PAH among individuals under 19 years of age globally and across various SDI countries. The significantly decreased trends among children under 12 months over the past three decades are likely due to improvements in the early diagnosis and management of CHDs, a major contributor to PAH in infants and young children [9, 10, 29]. Advances in neonatal care, early surgical interventions for CHDs, and the development of specialized pediatric PAH therapies have likely played a crucial role in reducing morbidity and mortality. In contrast, unimproved DALYs and deaths observed in children aged 10–19 years may reflect the challenges in managing PAH in this age group, where the diagnosis may be delayed, the transition from pediatric to adult care may be suboptimal, and the availability of age‐appropriate treatments may be limited. The clinical phenotypes of pediatric PAH are varied and frequently overlap. And the characteristics of the disease evolve throughout childhood [30, 31], which underscore the importance of continued improvements in neonatal care and CHD management, as well as the need for comprehensive care strategies for older children and adolescents with PAH. Despite similarities in PAH pathobiology between adults and children [9, 32], it is essential to recognize that children are not simply small adults. Considering the complexity of pediatric PAH, urgent research is needed to develop pediatric‐specific clinical evidence, improve patients’ prognoses, and address disparities among different SDI countries.

As China mainland and India account for approximately one‐third of the global PAH population, their efforts in disease management are pivotal for reducing the global PAH burden. Over the past three decades, the APC results show consistent trends in DALYs, deaths, and prevalence in both China mainland and India. Period and cohort effects indicate a significant decline in DALYs and deaths over time, with more pronounced improvements in China mainland. Both countries have seen an upward trend in prevalence, suggesting a gradual increase in the number of PAH cases (Figure S10). With its large population, China mainland has seen an increased focus on PAH management, resulting in standardized approaches and improved disease recognition. The government has made substantial strides in enhancing healthcare access, implementing national screening programs, and expanding access to targeted therapies. These initiatives have led to early disease detection, optimized treatment timing, and improved patient outcomes. The establishment and promotion of professional platforms [33] such as the China mainland Specialty Alliance for PH have driven rapid advancements in the diagnosis and treatment of pulmonary vascular diseases. In India, improvements in healthcare infrastructure, coupled with rising awareness among healthcare providers and patients, have contributed to better management and outcomes in recent years. However, limited training in PAH for physicians, the unavailability of targeted drugs, and socioeconomic barriers in the country significantly hindered further advancement of PAH management [34]. Owing to the density of the Chinese and Indian population and improved disease recognition, the number of patients with PAH is anticipated to continue increasing, necessitating sustained efforts to address the significant burden of the disease.

Prevalence estimates rely heavily on PAH recognition and complete data availability. “The more you look, the more you find” phenomenon [3] suggests that the true burden of PAH, particularly in low‐SDI regions, is likely underestimated due to inadequate disease recognition. Collaborative efforts are urgently needed to establish comprehensive diagnostic paradigms for PAH and to improve healthcare facilities. As awareness of the disease grows and right heart catheterization techniques become more accessible, more cases of PAH are increasingly being identified [35]. PAH is a multifactorial disease linked to comorbidities that include CHD [10, 36], connective tissue disease [37, 38], and endemic infections such as schistosomiasis [39] and human immunodeficiency virus [40], and it is prevalent in low‐SDI countries. This poses significant challenges for cost‐effective care delivery in developing countries. Preventive measures such as improvements in CHD surgery and global control of schistosomiasis could reduce the incidence of PAH in developing regions.

To our knowledge, this study represents the first comprehensive effort to describe the global and regional burdens of PAH. We provide evidence of sex and age differences during the past decades, depicting the epidemic characteristics of PAH. Most importantly, we also exhibit unfavorable effects in PAH burden among countries such as France, Germany, and China mainland, suggesting the focus on populations in specific periods and birth cohorts is urgently needed. In addition, the positive associations of SDI and UHCI with ASR of prevalence have been demonstrated for the first time, verifying the statement of “the more you look, the more you find” in the disease. The projections indicate a challenging future for PAH, with a significant decrease in DALYs cases over the next 30 years, particularly among males. However, the anticipated rise in PAH‐related deaths, especially among females, suggests that while medical advancements may reduce disability, mortality rates may continue to increase due to an aging population and improved diagnosis. The escalating prevalence of PAH, projected to nearly double by 2050, underscores the urgent need for enhanced disease management strategies and healthcare infrastructure to address this rising burden, particularly in high‐risk populations. Moreover, higher SDI and UHCI are associated with lower ASRs of DALYs and deaths but higher ASRs of PAH prevalence, emphasizing the importance of economic development and healthcare investments to urgently address the challenges posed by PAH.

Nevertheless, this study has several limitations. First, the limited availability of raw PAH data in less‐developed countries or regions has compromised the reliability of the APC model results, potentially leading to an underestimation of the disease burden. Future efforts should focus on strengthening PAH data collection in these countries. Second, the GBD presents data only at the national level, which has hindered the analysis of subnational differences essential for personalized regional policy formulation and implementation. Data from registry cohorts may be affected by study design, logistics, and the scope of clinical practice, introducing potential biases that impact reported epidemiological information. Third, detailed information on PAH subgroups, including idiopathic PAH and PAH associated with CHD or connective tissue disease is not provided. Furthermore, the absence of standardized global reporting systems for PAH risk factors, combined with the disease's rare nature, poses significant challenges in collecting comprehensive risk factor data at a global scale. Therefore, we suggest establishing international registries with standardized risk factors reporting protocols for future global burden analyses of PAH. Nonetheless, our research presents detailed insights into the global burden of PAH across different SDI regions and countries and has constructed APC and predictive models to further reveal the disease epidemiology, offering valuable evidence to inform policy guidance and improve disease management strategies.

In conclusion, the burden of PAH is differentiated, with notable disparities across regions and countries. This underscores the critical need for tailored healthcare policies in different countries to effectively address the challenges. Gap elimination and in‐depth exploration of PAH are urgently needed to improve the awareness, prevention, recognition, and management of PAH, particularly in resource‐limited countries. Despite the increased understanding of PAH in recent decades, considerable uncertainties persist, highlighting the importance of sustained global collaboration to develop preventive strategies and standardized treatments for more effective disease control.

## 4 Methods

### 4.1 Overview

An available dataset, the GBD, provides data compiled by the WHO and collaborating countries, accessible via the Global Health Data Exchange query tool (http://ghdx.healthdata.org/gbd‐results‐tool) for data extraction. Data collection is iterative and continuous to demonstrate dynamic disease changes. The GBD contains epidemiological data on 371 diseases and injuries across 204 countries and territories [41]. Compared with GBD 2019, the updated GBD 2021 includes statistics up to 2021 and introduces estimates for PAH. The GBD study adhered to ethical principles in data collection and study design, as detailed in previous publications [42, 43]. In this study, the DALYs, prevalence, and mortality of PAH were comprehensively analyzed based on the GBD 2021 dataset.

Briefly, our work is designed as an observational study to reveal the burden of PAH at global, regional, and national levels, thereby raising awareness of this fatal disease.

### 4.2 Data Source

PAH, classified as a Group 1 PH, is identified by ICD codes with diagnostic confirmation primarily based on right heart catheterization findings, and excluding other forms of PH. The GBD 2021 retrieved PAH data through a systematic review of the Global Index Medicus and PubMed databases from January 1, 1980 to May 2, 2021 [44]. Of the 7106 records screened, 65 were included for subsequent data analysis. The meta‐regression Bayesian, regularized and trimmed method was used to disaggregate both sex data points for prevalence and condition‐specific mortality into sex‐specific estimates. The DisMod‐MR 2.1 model was utilized to split data points where the age range exceeded 25 years. The following formula was used to calculate the condition‐specific mortality rate:

$$\mathrm{mtwith}=-\ln\left(1-\mathrm{cfr}\right)/\mathrm{time}\left(\mathrm{years}\right)$$

DisMod‐MR 2.1 was employed to estimate the prevalence of PAH utilizing covariates such as the healthcare access and quality index, the natural log of age‐standardized schistosomiasis prevalence, and an age‐standardized summary exposure value scalar for HIV prevalence.

Disability weights were applied to each patient with PAH to calculate unadjusted years lived with disability (YLDs). Through the comorbidity correction model, initial estimates were adjusted for comorbid conditions, resulting in comorbidity‐adjusted YLDs. The sum of the adjusted YLDs with the years of life lost (YLLs) due to premature mortality yielded estimates of DALYs.

### 4.3 DALYs, Deaths, and Prevalence

DALYs represent a comprehensive measure for assessing the total disease burden. They are calculated as the sum of YLDs, which quantify the loss of one full healthy year due to disability or illness, and YLLs, which measure the impact of premature mortality.

Prevalence signifies the total number of existing cases by the end of a year, reflecting the ubiquity of a disease. The definitions used to estimate the prevalence of PAH in GBD studies were based on the World Health Organization classification of the group 1 PH.

The DALYs, deaths, and prevalence attributable to PAH were estimated across different levels of geographical stratification, disaggregated by sex and spanning 25 years age groups (from 0 to 95 years and older) for each year from 1990 to 2021.

All reported measurements included numbers, age‐standardized rates (ASRs) per 100,000 people, and 95% UIs for all estimates. The 95% UIs were derived from the 2.5th and 97.5th ordinals of 500 draws from the posterior distribution [45].

### 4.4 Global Regions

Our analysis included 204 countries and territories [46]. This geographical division aids in understanding and researching the global distribution of the disease burden, facilitating detailed analyses and comparisons across various countries to inform targeted public health interventions and policies.

### 4.5 SDI and UHCI

The SDI is a composite metric derived from three key factors: per capita income, average education level among individuals aged 15 years and older, and total fertility rate among females under 25 years of age. This index reflects social and economic conditions and serves as a comprehensive indicator of the developmental status of a country or region. In the 2021 GBD study, all countries and territories were categorized into five SDI levels: low (<0.46), low‐middle (0.46–0.62), middle (0.62–0.71), high‐middle (0.71–0.81), and high (>0.81); detailed information is provided in Table S1 [47]. This study also included an analysis of the disease burden across these SDI categories.

The UHCI is a composite measure used to evaluate individuals’ access to essential health services without suffering financial hardship. It typically includes indicators that assess service coverage and financial protection. The index aims to reflect the availability, accessibility, and quality of health services, as well as the equity and financial risk protection provided by a healthcare system. The goal of the UHCI is to ensure that everyone can obtain the health services they need without being pushed into poverty by healthcare costs [48] (https://www.who.int/data/gho/indicator‐metadata‐registry/imr‐details/4834).

The associations of SDI and UHCI with DALYs, deaths, and prevalence were explored.

### 4.6 Statistical Analysis

Our analysis included diverse age groups and both sexes to comprehensively illustrate the global burden of PAH from 1990 to 2021, as measured by DALYs, deaths, and prevalence. The EAPC, calculated based on linear regression models and natural logarithm fitting data, was used to quantify the changes over a specific time interval. EAPC was calculated using the formula 100 × (eβ − 1), and more specific information was provided in a previous study [49]. In this study, we used the EAPC to estimate the trends in ASR from 1990 to 2021.

To explore the correlations of PAH DALYs, deaths, and prevalence with the SDI and UHCI, we performed a Pearson correlation analysis and fitted the linear trend model globally. The UHCI data were obtained from the World Health Organization website (https://www.who.int/data/gho/data/indicators/indicator‐details/GHO/uhc‐index‐of‐service‐coverage).

The APC model examined the effects of age, period, and birth cohort on trends in PAH burden, addressing collinearity among age, period, and cohort, offering a distinct advantage over conventional epidemiological analyses [10]. The detailed methodology has been reported in previous studies [50, 51]. Specifically, the prevalence and population numbers of each country or region were set as the input data. Because of the scarce number of PAH cases above 85 years (Figure S1), this analysis mainly included populations aged 0–84 years. The study period, from 1992–1996 to 2017–2021, was segmented into six 5‐year periods. Age was categorized into 17 groups, ranging from 0–4 years and up to 80–84 years at 5‐year intervals. The birth cohorts were divided into 22 groups from 1908–1916 to 2013–2021 at 5‐year intervals (Table S2). The APC output included net and local drifts, age, period, and cohort effects, as defined in our previous studies [10, 52, 53, 54].

We generated forecasts for global PAH burden using the number and ASRs of DALYs, deaths, and prevalence. Detailed information about the forecast methods was mentioned in the previous study [55]. Briefly, for prevalence and mortality projections, we used data from 1990, 1995, 2000, 2005, 2010, 2015, and 2019 as input for our regression models. For DALYs, considering the distinct temporal pattern (initial decline followed by an increase before 2012, and a continuous decrease thereafter), we utilized data points from 2012, 2014, 2016, 2018, 2020, and 2021 to better capture the current declining trend to improve forecasting accuracy. Our regression models included year and age as predictors, with a cubic spline term for age to account for nonlinear age effects. Sex‐specific models were run 1000 times, and the resulting coefficients were used to predict rates for 2030, 2040, and 2050. The predicted rates were then multiplied by forecasted global population estimates to obtain the absolute numbers of cases, deaths, and DALYs. Additionally, we calculated ASRs using the WHO World Standard Population.

All analyses were performed using R software (version 4.3.2). Significance was determined using two‐tailed *p* values, with statistical significance set at *p* < 0.05.

## Author Contributions

Yicheng Yang: Writing—original draft, project administration, investigation, funding acquisition, formal analysis, conceptualization; Zhiwei Zeng: Writing—original draft, formal analysis, and conceptualization; Qiaoxi Yang: Writing—original draft, validation, methodology; Huan Wang: Formal analysis and validation; Hanwen Zhang, Wenjie Yan, Peizhi Wang, Chuangshi Wang, and Zhanhao Su: Validation and methodology; Pugazhenthan Thangaraju and Sher Zaman Safi: Writing—review and editing and validation; Beilan Yang and Yaoyao Wang: Visualization; Jingjing Zhou: Funding acquisition and writing—review; Yuan Huang: Funding acquisition, supervision, and writing—review; Zhiyong Zou: Writing—review and editing, supervision, and conceptualization; Songren Shu and Changming Xiong: Writing—review and editing, funding acquisition, data curation, and conceptualization. All authors have read and approved the final manuscript.

## Ethics Statement

The GBD study adhered to ethical principles in data collection and study design.

## Conflicts of Interest

The authors declare no conflicts of interest.

## Supporting information



Supporting Information

## Data Availability

The data underlying this article were derived from sources in the public domain, namely, the Institute for Health Metrics and Evaluation (IHME), at https://vizhub.healthdata.org/gbd‐results.
