# Supplementary material for: The Challenge in Burden of Pulmonary Arterial Hypertension: A Perspective From the Global Burden of Disease Study
Source: MedComm (2020). 2025 Apr 24;6(5):e70175. doi: 10.1002/mco2.70175 (PMC12019876; doi:10.1002/mco2.70175)
Supplement: Supplementary file 1 — Supporting Information [file MCO2-6-e70175-s001.pdf]

Supplementary Materials

The Challenge in Burden of Pulmonary Arterial Hypertension: A Pperspective From the Global Burden of Disease Study

Yicheng Yang, MD[1, 2, 3#], Zhiwei Zeng, MD[1#], Qiaoxi Yang, MD[1#], Huan Wang, PhD[4#], Hanwen Zhang, MD[1, 2], Wenjie Yan, MD [1, 2], Peizhi Wang, MD[1, 5], Chuangshi Wang, PhD[1, 6], Zhanhao Su, MD[7], Pugazhenthan Thangaraju, PhD[8], Sher Zaman Safi, MSc, MPhil, PhD[9], Beilan Yang[1, 2], Yaoyao Wang, MD[1], Jingjing Zhou, MD, PhD[10], Zhiyong Zou, PhD[4*], Yuan Huang, MD[1, 11*], Songren Shu, MD[1, 11*], Changming Xiong, MD, PhD[1, 2*]

[1] State Key Laboratory of Cardiovascular Disease, Fuwai Hospital, National Center for Cardiovascular Diseases, Chinese Academy of Medical Sciences and Peking Union Medical College, Beijing, China.

[2] Center of Respiratory and Pulmonary Vascular Disease, Fuwai Hospital, National Center for Cardiovascular Disease, Chinese Academy of Medical Sciences and Peking Union Medical College, Beijing, China.

[3] Department of Cardiology, Anzhen Hospital, Beijing, China.

[4] Institute of Child and Adolescent Health, School of Public Health, National Health Commission Key Laboratory of Reproductive Health, Peking University, No. 38 Xueyuan Rd, Haidian District, Beijing, 100191, China.

[5] Center for Molecular Cardiology, University of Zurich, Wagistrasse 12, CH 8952 Schlieren, Zurich, Switzerland.

[6] Medical Research and Biometrics Center, National Clinical Research Center for

Cardiovascular Diseases, Fuwai Hospital, National Center for Cardiovascular Diseases, Peking Union Medical College &Chinese Academy of Medical Sciences, Beijing, China.

[7] Department of Cardiovascular Surgery, Guangdong Cardiovascular Institute, Guangdong Provincial People's Hospital, Guangdong Academy of Medical Sciences, Guangzhou, China.

[8] All India Institute of Medical Sciences, Tatibandh, Raipur, India.

[9] Faculty of Medicine, Bioscience & Nursing, MAHSA University, Selangor, Malaysia.

[10] Echocardiography Medical Center, Beijing Anzhen Hospital, Capital Medical University, Beijing, China.

[11] Department of Cardiovascular Surgery, Fuwai Hospital, National Center for Cardiovascular Diseases, Chinese Academy of Medical Sciences and Peking Union Medical College, Beijing, China.

[#] These authors contributed equally.

[*]Correspondence

Changming Xiong, 167 Beilishi Road, Xicheng District, Beijing, 100037, China. xiongchangming@fuwai.com

Songren Shu, 167 Beilishi Road, Xicheng District, Beijing, 100037, China. shusongren@fuwai.com

Yuang Huang, 167 Beilishi Road, Xicheng District, Beijing, 100037, China. huangyuan@fuwai.com

Zhiyong Zou, No. 38 Xueyuan Road, Haidian District, Beijing, 100191, China. harveyzou2002@bjmu.edu.cn

Short title: Burden of Pulmonary Arterial Hypertension

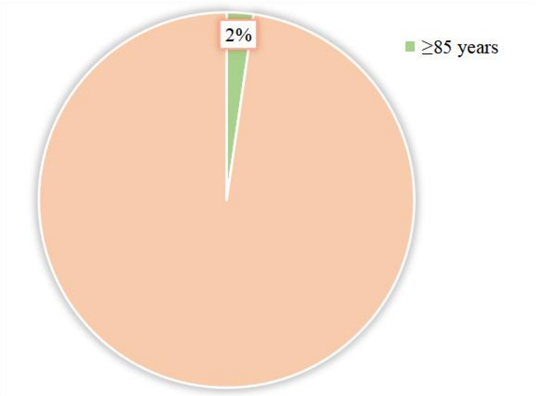

Figure S1. The proportion of PAH population with age more than 85 years old.

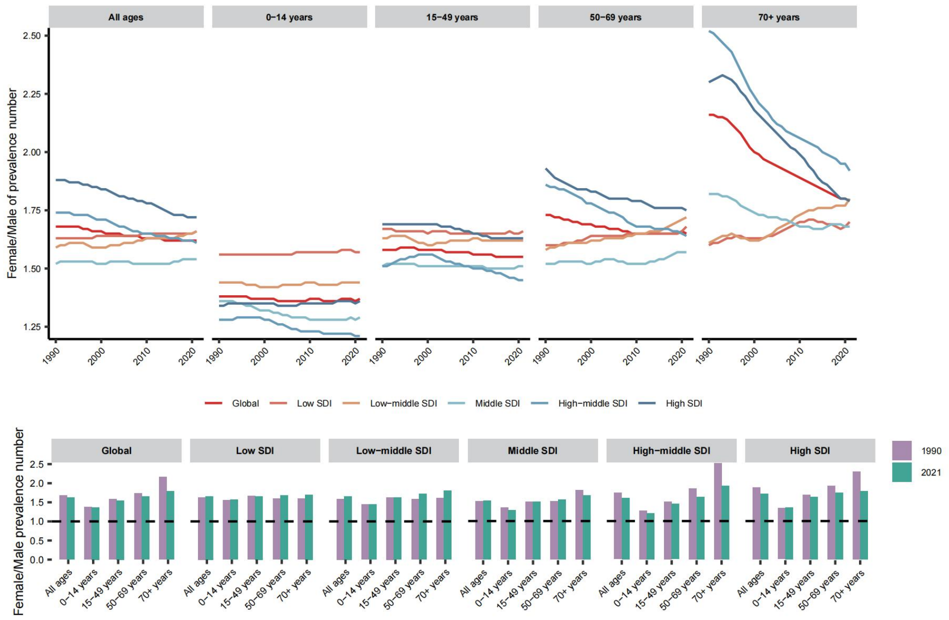

Figure S2. Prevalence shifts across age and sex difference. The female/male proportion of prevalence number among different age groups including 0-14 years, 15-49 years, 50-69 years, and more than 70 years groups from 1990 to 2021. SDI: Sociodemographic index.

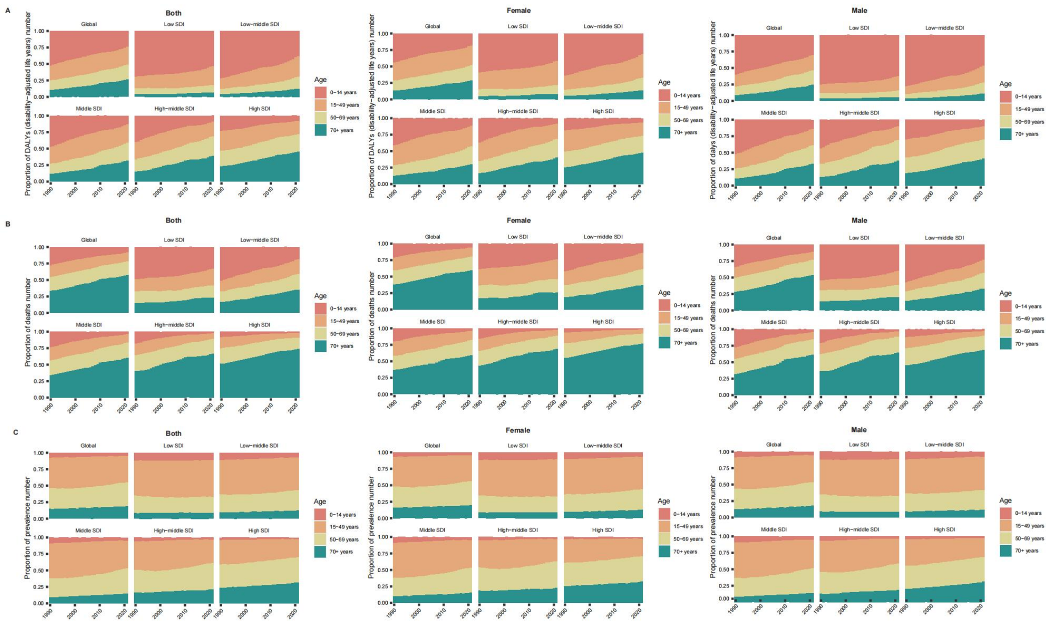

Figure S3. DALYs, death, and prevalence shifts across age and sex difference. (A) The DALYs proportion of different age groups including 0-14 years, 15-49 years, 50-69 years, and more than 70 years groups among total, female, and male populations from 1990 to 2021. (B) The death proportion of different age groups including 0-14 years, 15-49 years, 50-69 years, and more than 70 years groups among total, female, and male populations from 1990 to 2021. (C) The prevalence proportion of different age groups including 0-14 years, 15-49 years, 50-69 years, and more than 70 years groups among total, female, and male populations from 1990 to 2021. DALYs: Disability adjusted life years.

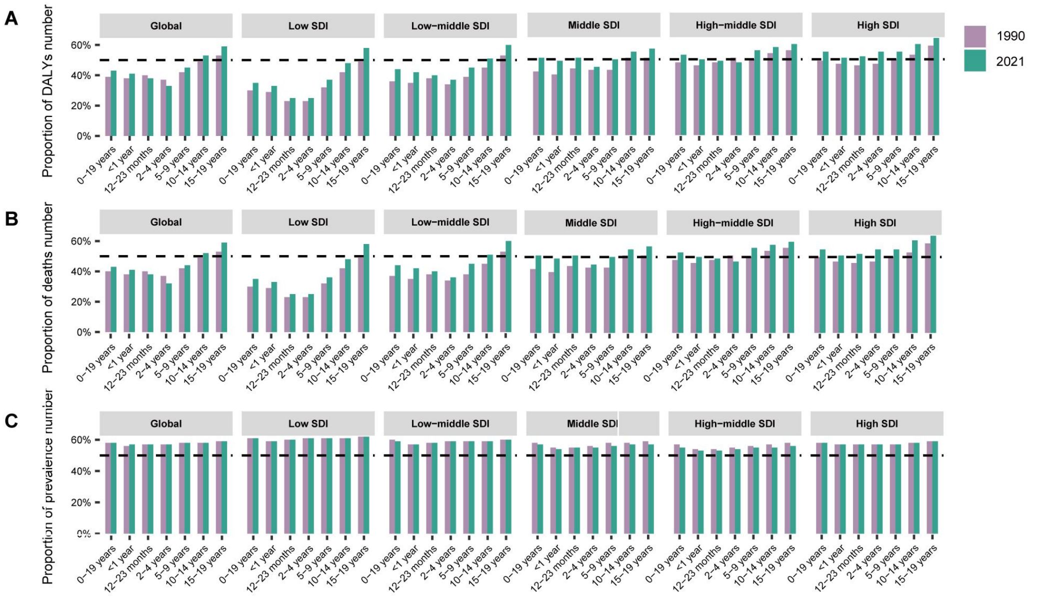

Figure S4. Female proportion of (A) DALYs, (B) deaths, and (C) prevalence in different age groups in 1990 and 2021. DALYs: Disability adjusted life years; SDI: Sociodemographic index.

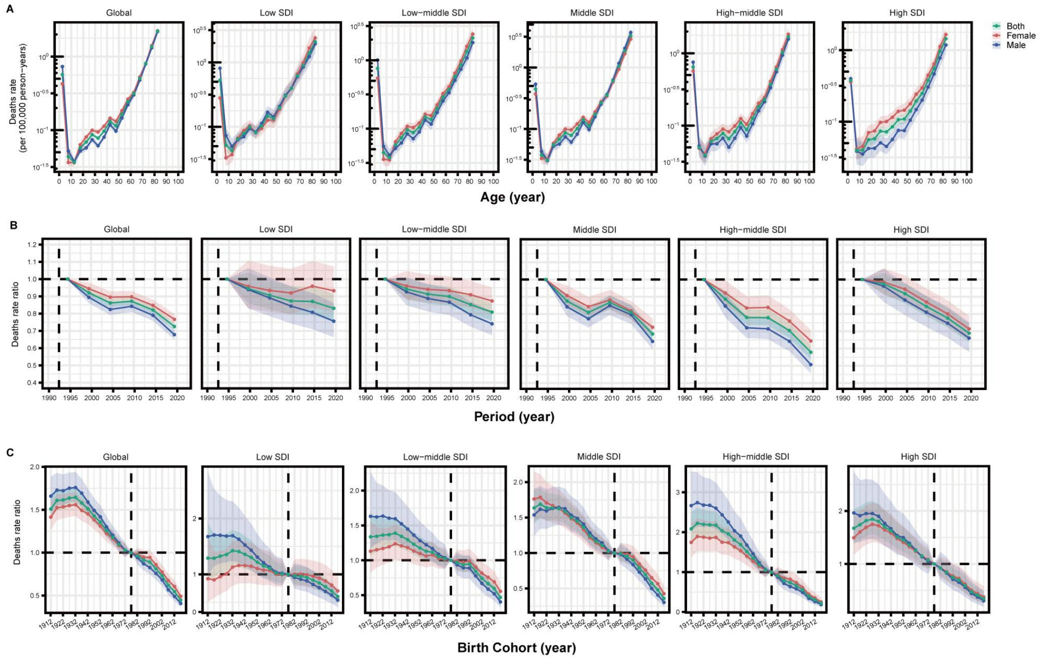

Figure S5. Age-period-cohort models of PAH death in global and five SDI quantiles. (A) Age effects show the fitted longitudinal age curves of PAH death rate (per 100,000 person-years) and the corresponding 95% CIs. (B) Period effects show the relative risk of PAH death rate of each period compared with the reference (period 1992–1996) adjusted for age and nonlinear cohort effects and the corresponding 95% CIs. (C) Cohort effects show the relative risk of PAH death rate of each cohort compared with the reference (cohort 1921–1929) adjusted for age and nonlinear period effects and the corresponding 95% CIs. PAH: Pulmonary arterial hypertension; SDI: Sociodemographic index; CIs: Confidence intervals.

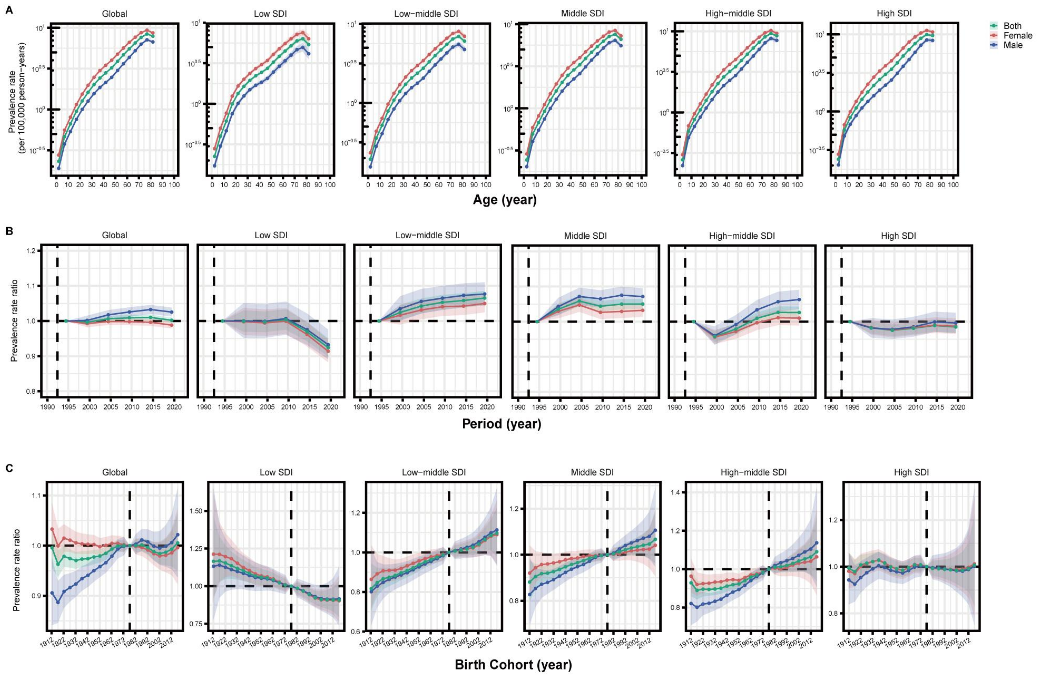

Figure S6. Age-period-cohort models of PAH prevalence in global and five SDI quantiles. (A) Age effects show the fitted longitudinal age curves of PAH prevalence rate (per 100,000 person-years) and the corresponding 95% CIs. (B) Period effects show the relative risk of PAH prevalence rate of each period compared with the reference (period 1992–1996) adjusted for age and nonlinear cohort effects and the corresponding 95% CIs. (C) Cohort effects show the relative risk of PAH prevalence rate of each cohort compared with the reference (cohort 1921–1929) adjusted for age and nonlinear period effects and the corresponding 95% CIs. PAH: Pulmonary arterial hypertension; SDI: Sociodemographic index; CIs: Confidence intervals.

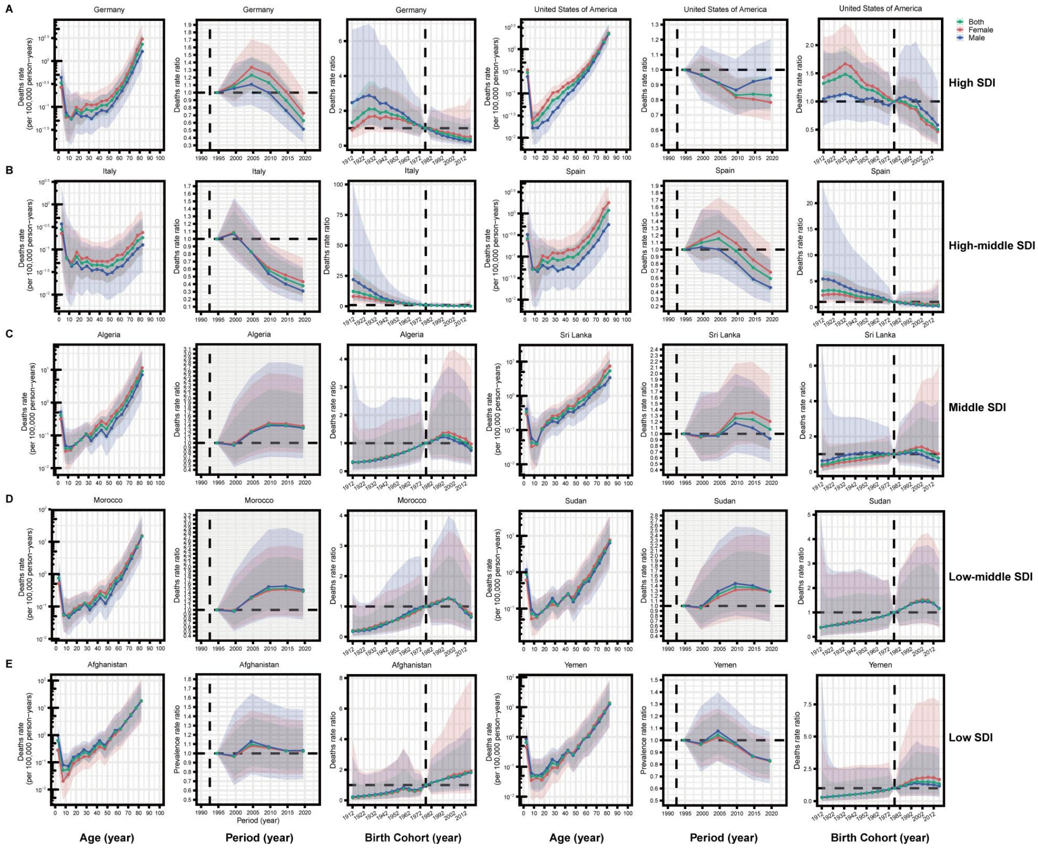

Figure S7. Exemplar countries across different regions and SDI quantiles showing unfavourable age-period-cohort effects in PAH deaths. Ten countries including (A) two high-SDI regions (Germany and United States of America), (B) two high-middle-SDI countries (Italy and Spain), (C) two middle-SDI countries (Algeria and Sri Lanka), (D) two low-middle-SDI countries (Morocco and Sudan), and (E) two low-SDI countries (Afghanistan and Yemen) were selected to exhibit the unsatisfactory APC effects in death. Overall trends in the deaths rate from 1990 to 2021. The fitted longitudinal age curves display the deaths rate of PAH per 100,000 person-years, along with the corresponding 95% CIs. Period effects illustrate the relative risk of PAH deaths for each period, compared to the reference period of

1992–1996, adjusted for age and nonlinear cohort effects, and include the corresponding 95% CIs. Cohort effects show the relative risk of PAH deaths for each cohort, using the 1921–1929 cohort as the reference, adjusted for age and nonlinear period effects, with the corresponding 95% CIs provided. PAH: Pulmonary arterial hypertension; SDI: Sociodemographic index; CIs: Confidence intervals.

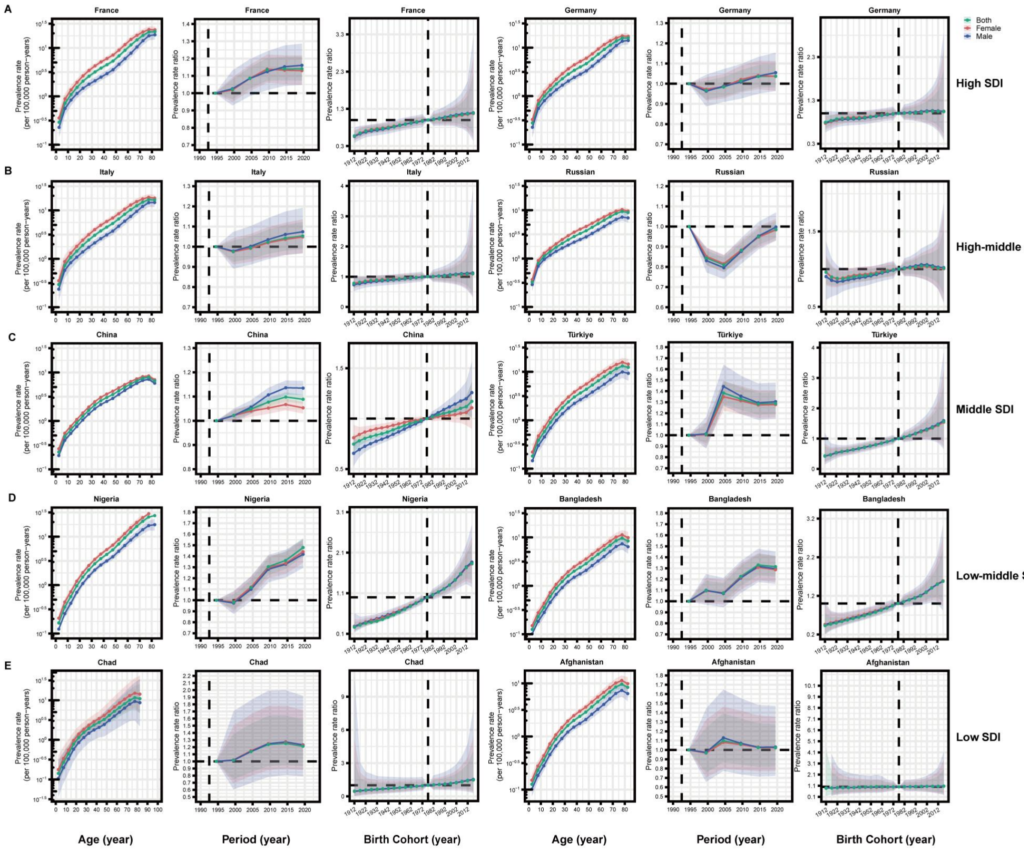

Figure S8. Exemplar countries across different regions and SDI quantiles showing unfavourable age-period-cohort effects in PAH prevalence. Ten countries including (A) two high-SDI countries (France and Germany), (B) two high-middle-SDI countries (Italy and the Russian Federation), (C) two middle-SDI countries (China mainland and Türkiye), (D) two low-middle-SDI countries (Nigeria and Bangladesh), and (E) two low-SDI countries (Chad and Afghanistan) were selected to exhibit the unsatisfactory APC effects in prevalence. Overall trends in the prevalence rate from 1990 to 2021. The fitted longitudinal age curves display the prevalence rate of PAH per 100,000 person-years, along with the corresponding 95% CIs. Period effects illustrate the relative risk of PAH prevalence for each period,

compared to the reference period of 1992–1996, adjusted for age and nonlinear cohort effects, and include the corresponding 95% CIs. Cohort effects show the relative risk of PAH prevalence for each cohort, using the 1921–1929 cohort as the reference, adjusted for age and nonlinear period effects, with the corresponding 95% CIs provided. PAH: Pulmonary arterial hypertension; SDI: Sociodemographic index; CIs: Confidence intervals.

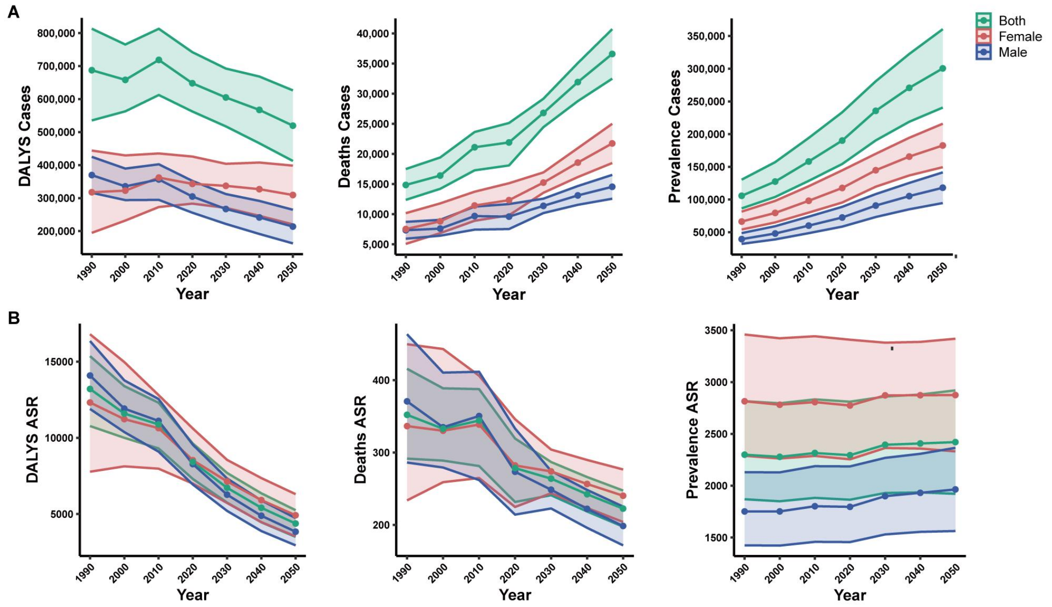

Figure S9. PAH burden projections through 2050 across different sex population.

(A) The projections in global cases of DALYs, deaths, and prevalence were presented; (B) The projections in global ASRs of DALYs, deaths, and prevalence were presented. PAH: Pulmonary arterial hypertension; DALYs: Disability-adjusted life year; ASRs: age-standardized rates.

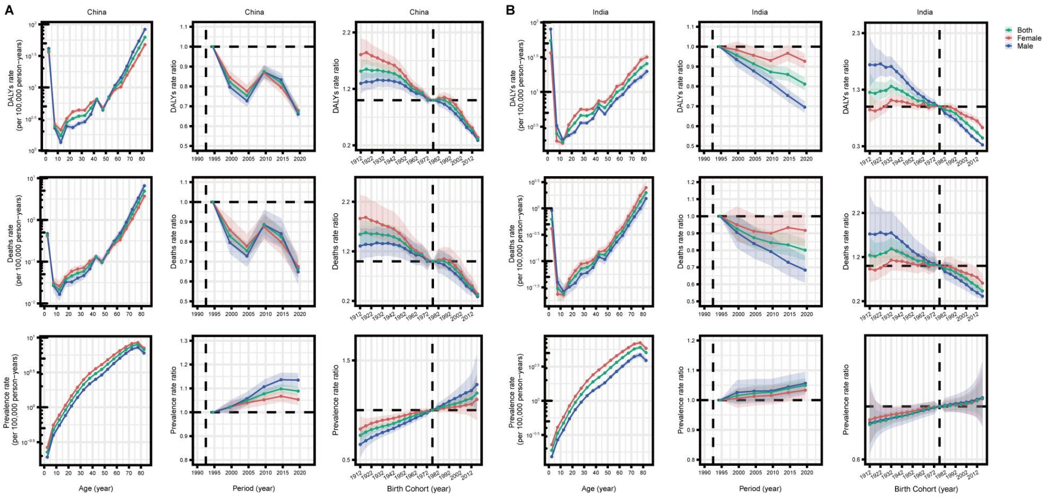

Figure S10. Age-period-cohort effects in China mainland (A) and India (B). Overall trends in the DALYs, death, and prevalence rate from 1990 to 2021. The fitted longitudinal age curves display the rates of PAH per 100,000 person-years, along with the corresponding 95% CIs. Period effects illustrate the relative risk of PAH DALYs, death, and prevalence for each period, compared to the reference period of 1992 – 1996, adjusted for age and nonlinear cohort effects, and include the corresponding 95% CIs. Cohort effects show the relative risk of PAH DALYs, death, and prevalence for each cohort, using the 1921 – 1929 cohort as the reference, adjusted for age and nonlinear period effects, with the corresponding 95% CIs provided. PAH: Pulmonary arterial hypertension; SDI: Sociodemographic index; CIs: Confidence intervals.

Supplemental Tables

Table S1. Detailed SDI information of countries and territories

Location name	Year	Mean	Lower	Upper
Global	2021	0.665820975	0.665820975	0.665820975
Southeast Asia, East Asia, and Oceania	2021	0.698849901	0.698849901	0.698849901
East Asia	2021	0.725704902	0.725704902	0.725704902
China mainland	2021	0.72162976	0.72162976	0.72162976
Democratic People's Republic of Korea	2021	0.569854634	0.569854634	0.569854634
Taiwan (Province of China mainland)	2021	0.874747053	0.874747053	0.874747053
Southeast Asia	2021	0.649777295	0.649777295	0.649777295
Cambodia	2021	0.473621491	0.473621491	0.473621491
Indonesia	2021	0.656868336	0.656868336	0.656868336
Lao People's Democratic Republic	2021	0.489136091	0.489136091	0.489136091
Malaysia	2021	0.742523828	0.742523828	0.742523828
Maldives	2021	0.650886627	0.650886627	0.650886627
Myanmar	2021	0.53390084	0.53390084	0.53390084
Philippines	2021	0.651219329	0.651219329	0.651219329
Sri Lanka	2021	0.701534935	0.701534935	0.701534935
Thailand	2021	0.682547933	0.682547933	0.682547933
Timor-Leste	2021	0.444667619	0.444667619	0.444667619
Viet Nam	2021	0.627933721	0.627933721	0.627933721
Oceania	2021	0.467445126	0.467445126	0.467445126
Fiji	2021	0.675051631	0.675051631	0.675051631
Kiribati	2021	0.527186583	0.527186583	0.527186583
Marshall Islands	2021	0.574091128	0.574091128	0.574091128
Micronesia (Federated States of)	2021	0.587534967	0.587534967	0.587534967
Papua New Guinea	2021	0.417797443	0.417797443	0.417797443
Samoa	2021	0.593392769	0.593392769	0.593392769
Solomon Islands	2021	0.429360316	0.429360316	0.429360316
Tonga	2021	0.626349936	0.626349936	0.626349936
Vanuatu	2021	0.473100706	0.473100706	0.473100706
Central Europe, Eastern Europe, and Central Asia	2021	0.769466346	0.769466346	0.769466346
Central Asia	2021	0.675163978	0.675163978	0.675163978
Armenia	2021	0.701833194	0.701833194	0.701833194
Azerbaijan	2021	0.694851274	0.694851274	0.694851274
Georgia	2021	0.732473604	0.732473604	0.732473604
Kazakhstan	2021	0.725144495	0.725144495	0.725144495
Kyrgyzstan	2021	0.603979328	0.603979328	0.603979328

Mongolia	2021	0.617621565	0.617621565	0.617621565
Tajikistan	2021	0.541511187	0.541511187	0.541511187
Turkmenistan	2021	0.682160776	0.682160776	0.682160776
Uzbekistan	2021	0.662621694	0.662621694	0.662621694
Central Europe	2021	0.796244448	0.796244448	0.796244448
Albania	2021	0.706849791	0.706849791	0.706849791
Bosnia and Herzegovina	2021	0.723077893	0.723077893	0.723077893
Bulgaria	2021	0.768150939	0.768150939	0.768150939
Croatia	2021	0.798341027	0.798341027	0.798341027
Czechia	2021	0.828450433	0.828450433	0.828450433
Hungary	2021	0.790754768	0.790754768	0.790754768
North Macedonia	2021	0.750629703	0.750629703	0.750629703
Montenegro	2021	0.795800584	0.795800584	0.795800584
Poland	2021	0.812042809	0.812042809	0.812042809
Romania	2021	0.768453864	0.768453864	0.768453864
Serbia	2021	0.792416294	0.792416294	0.792416294
Slovakia	2021	0.81061053	0.81061053	0.81061053
Slovenia	2021	0.842430731	0.842430731	0.842430731
Eastern Europe	2021	0.802851009	0.802851009	0.802851009
Belarus	2021	0.784484711	0.784484711	0.784484711
Estonia	2021	0.844917787	0.844917787	0.844917787
Latvia	2021	0.830663516	0.830663516	0.830663516
Lithuania	2021	0.856484049	0.856484049	0.856484049
Republic of Moldova	2021	0.732214875	0.732214875	0.732214875
Russian Federation	2021	0.808536005	0.808536005	0.808536005
Ukraine	2021	0.760773913	0.760773913	0.760773913
High-income	2021	0.85188481	0.85188481	0.85188481
High-income Asia Pacific	2021	0.876767	0.876767	0.876767
Brunei Darussalam	2021	0.810234367	0.810234367	0.810234367
Japan	2021	0.871241813	0.871241813	0.871241813
Republic of Korea	2021	0.886675267	0.886675267	0.886675267
Singapore	2021	0.856097766	0.856097766	0.856097766
Australasia	2021	0.845514063	0.845514063	0.845514063
Australia	2021	0.844252814	0.844252814	0.844252814
New Zealand	2021	0.849442499	0.849442499	0.849442499
Western Europe	2021	0.848726316	0.848726316	0.848726316
Andorra	2021	0.869444113	0.869444113	0.869444113
Austria	2021	0.853837004	0.853837004	0.853837004
Belgium	2021	0.853654016	0.853654016	0.853654016
Cyprus	2021	0.835630545	0.835630545	0.835630545
Denmark	2021	0.896424204	0.896424204	0.896424204
Finland	2021	0.859831368	0.859831368	0.859831368
France	2021	0.838364875	0.838364875	0.838364875
Germany	2021	0.902957091	0.902957091	0.902957091
Greece	2021	0.791854408	0.791854408	0.791854408
Iceland	2021	0.87636168	0.87636168	0.87636168
Ireland	2021	0.87375385	0.87375385	0.87375385

Israel	2021	0.809011652	0.809011652	0.809011652
Italy	2021	0.805773534	0.805773534	0.805773534
Luxembourg	2021	0.884428955	0.884428955	0.884428955
Malta	2021	0.801585034	0.801585034	0.801585034
Netherlands	2021	0.888464256	0.888464256	0.888464256
Norway	2021	0.91613281	0.91613281	0.91613281
Portugal	2021	0.744151851	0.744151851	0.744151851
Spain	2021	0.769283698	0.769283698	0.769283698
Sweden	2021	0.886880299	0.886880299	0.886880299
Switzerland	2021	0.933059111	0.933059111	0.933059111
United Kingdom	2021	0.859000182	0.859000182	0.859000182
Southern Latin America	2021	0.735984717	0.735984717	0.735984717
Argentina	2021	0.723122973	0.723122973	0.723122973
Chile	2021	0.771514716	0.771514716	0.771514716
Uruguay	2021	0.719283445	0.719283445	0.719283445
High-income North America	2021	0.863465474	0.863465474	0.863465474
Canada	2021	0.87317068	0.87317068	0.87317068
United States of America	2021	0.862448354	0.862448354	0.862448354
Latin America and Caribbean	2021	0.646541285	0.646541285	0.646541285
Caribbean	2021	0.642003055	0.642003055	0.642003055
Antigua and Barbuda	2021	0.749886887	0.749886887	0.749886887
Bahamas	2021	0.805020668	0.805020668	0.805020668
Barbados	2021	0.746748764	0.746748764	0.746748764
Belize	2021	0.610229002	0.610229002	0.610229002
Cuba	2021	0.668729864	0.668729864	0.668729864
Dominica	2021	0.746967185	0.746967185	0.746967185
Dominican Republic	2021	0.619388201	0.619388201	0.619388201
Grenada	2021	0.668993028	0.668993028	0.668993028
Guyana	2021	0.650812335	0.650812335	0.650812335
Haiti	2021	0.448278285	0.448278285	0.448278285
Jamaica	2021	0.683263064	0.683263064	0.683263064
Saint Lucia	2021	0.672509735	0.672509735	0.672509735
Saint Vincent and the Grenadines	2021	0.637195963	0.637195963	0.637195963
Suriname	2021	0.633665739	0.633665739	0.633665739
Trinidad and Tobago	2021	0.768763254	0.768763254	0.768763254
Andean Latin America	2021	0.651602456	0.651602456	0.651602456
Bolivia (Plurinational State of)	2021	0.599010799	0.599010799	0.599010799
Ecuador	2021	0.661017053	0.661017053	0.661017053
Peru	2021	0.662054037	0.662054037	0.662054037
Central Latin America	2021	0.6406851	0.6406851	0.6406851
Colombia	2021	0.655442913	0.655442913	0.655442913
Costa Rica	2021	0.700340477	0.700340477	0.700340477
El Salvador	2021	0.563775188	0.563775188	0.563775188
Guatemala	2021	0.539972424	0.539972424	0.539972424
Honduras	2021	0.513037248	0.513037248	0.513037248
Mexico	2021	0.664575304	0.664575304	0.664575304
Nicaragua	2021	0.523958472	0.523958472	0.523958472

Panama	2021	0.708864828	0.708864828	0.708864828
Venezuela (Bolivarian Republic of)	2021	0.596513059	0.596513059	0.596513059
Tropical Latin America	2021	0.652442394	0.652442394	0.652442394
Brazil	2021	0.653043887	0.653043887	0.653043887
Paraguay	2021	0.635718099	0.635718099	0.635718099
North Africa and Middle East	2021	0.658224715	0.658224715	0.658224715
North Africa and Middle East	2021	0.658224715	0.658224715	0.658224715
Algeria	2021	0.659500924	0.659500924	0.659500924
Bahrain	2021	0.753043204	0.753043204	0.753043204
Egypt	2021	0.606787094	0.606787094	0.606787094
Iran (Islamic Republic of)	2021	0.697207398	0.697207398	0.697207398
Iraq	2021	0.662626231	0.662626231	0.662626231
Jordan	2021	0.725307227	0.725307227	0.725307227
Kuwait	2021	0.846651055	0.846651055	0.846651055
Lebanon	2021	0.744746351	0.744746351	0.744746351
Libya	2021	0.725771399	0.725771399	0.725771399
Morocco	2021	0.562698301	0.562698301	0.562698301
Palestine	2021	0.631011665	0.631011665	0.631011665
Oman	2021	0.773391602	0.773391602	0.773391602
Qatar	2021	0.846860584	0.846860584	0.846860584
Saudi Arabia	2021	0.815143493	0.815143493	0.815143493
Syrian Arab Republic	2021	0.623004075	0.623004075	0.623004075
Tunisia	2021	0.682432216	0.682432216	0.682432216
Türkiye	2021	0.712692673	0.712692673	0.712692673
United Arab Emirates	2021	0.849317734	0.849317734	0.849317734
Yemen	2021	0.450376375	0.450376375	0.450376375
South Asia	2021	0.557864657	0.557864657	0.557864657
South Asia	2021	0.557864657	0.557864657	0.557864657
Afghanistan	2021	0.337199998	0.337199998	0.337199998
Bangladesh	2021	0.492420885	0.492420885	0.492420885
Bhutan	2021	0.473062378	0.473062378	0.473062378
India	2021	0.575401649	0.575401649	0.575401649
Nepal	2021	0.433174635	0.433174635	0.433174635
Pakistan	2021	0.504028689	0.504028689	0.504028689
Sub-Saharan Africa	2021	0.458587301	0.458587301	0.458587301
Central Sub-Saharan Africa	2021	0.472255651	0.472255651	0.472255651
Angola	2021	0.453721949	0.453721949	0.453721949
Central African Republic	2021	0.30916769	0.30916769	0.30916769
Congo	2021	0.583075236	0.583075236	0.583075236
Democratic Republic of the Congo	2021	0.383179849	0.383179849	0.383179849
Equatorial Guinea	2021	0.657857456	0.657857456	0.657857456
Gabon	2021	0.634691393	0.634691393	0.634691393
Eastern Sub-Saharan Africa	2021	0.409720983	0.409720983	0.409720983
Burundi	2021	0.289374365	0.289374365	0.289374365
Comoros	2021	0.475978688	0.475978688	0.475978688
Djibouti	2021	0.487958371	0.487958371	0.487958371
Eritrea	2021	0.403863943	0.403863943	0.403863943

Ethiopia	2021	0.358823295	0.358823295	0.358823295
Kenya	2021	0.523768077	0.523768077	0.523768077
Madagascar	2021	0.400246943	0.400246943	0.400246943
Malawi	2021	0.384553634	0.384553634	0.384553634
Mauritius	2021	0.718260446	0.718260446	0.718260446
Mozambique	2021	0.326462614	0.326462614	0.326462614
Rwanda	2021	0.435588706	0.435588706	0.435588706
Seychelles	2021	0.730150775	0.730150775	0.730150775
Somalia	2021	0.077688109	0.077688109	0.077688109
United Republic of Tanzania	2021	0.446568273	0.446568273	0.446568273
Uganda	2021	0.423261181	0.423261181	0.423261181
Zambia	2021	0.505948954	0.505948954	0.505948954
Southern Sub-Saharan Africa	2021	0.642200282	0.642200282	0.642200282
Botswana	2021	0.642721629	0.642721629	0.642721629
Lesotho	2021	0.510393066	0.510393066	0.510393066
Namibia	2021	0.617564872	0.617564872	0.617564872
South Africa	2021	0.679626598	0.679626598	0.679626598
Eswatini	2021	0.585459713	0.585459713	0.585459713
Zimbabwe	2021	0.473819486	0.473819486	0.473819486
Western Sub-Saharan Africa	2021	0.446022979	0.446022979	0.446022979
Benin	2021	0.373486574	0.373486574	0.373486574
Burkina Faso	2021	0.285118402	0.285118402	0.285118402
Cameroon	2021	0.479691223	0.479691223	0.479691223
Cabo Verde	2021	0.533534539	0.533534539	0.533534539
Chad	2021	0.240436019	0.240436019	0.240436019
Côte d'Ivoire	2021	0.425941883	0.425941883	0.425941883
Gambia	2021	0.40971416	0.40971416	0.40971416
Ghana	2021	0.56493039	0.56493039	0.56493039
Guinea	2021	0.336401293	0.336401293	0.336401293
Guinea-Bissau	2021	0.353109621	0.353109621	0.353109621
Liberia	2021	0.352442452	0.352442452	0.352442452
Mali	2021	0.268579941	0.268579941	0.268579941
Mauritania	2021	0.4989451	0.4989451	0.4989451
Niger	2021	0.168072774	0.168072774	0.168072774
Nigeria	2021	0.503390833	0.503390833	0.503390833
Sao Tome and Principe	2021	0.505413747	0.505413747	0.505413747
Senegal	2021	0.408054193	0.408054193	0.408054193
Sierra Leone	2021	0.358665881	0.358665881	0.358665881
Togo	2021	0.408533695	0.408533695	0.408533695
American Samoa	2021	0.723727533	0.723727533	0.723727533
Bermuda	2021	0.821365422	0.821365422	0.821365422
Cook Islands	2021	0.779109955	0.779109955	0.779109955
Greenland	2021	0.826210336	0.826210336	0.826210336
Guam	2021	0.803982203	0.803982203	0.803982203
Monaco	2021	0.908262831	0.908262831	0.908262831
Nauru	2021	0.625177834	0.625177834	0.625177834
Niue	2021	0.72622205	0.72622205	0.72622205

Northern Mariana Islands	2021	0.771535213	0.771535213	0.771535213
Palau	2021	0.754046931	0.754046931	0.754046931
Puerto Rico	2021	0.825525847	0.825525847	0.825525847
Saint Kitts and Nevis	2021	0.754987055	0.754987055	0.754987055
San Marino	2021	0.888005474	0.888005474	0.888005474
Tokelau	2021	0.686425621	0.686425621	0.686425621
Tuvalu	2021	0.576620529	0.576620529	0.576620529
United States Virgin Islands	2021	0.821830853	0.821830853	0.821830853
Northern Ireland	2021	0.838531942	0.838531942	0.838531942
Scotland	2021	0.852061416	0.852061416	0.852061416
South Sudan	2021	0.278371125	0.278371125	0.278371125
Eastern Cape	2021	0.617204696	0.617204696	0.617204696
Free State	2021	0.677908602	0.677908602	0.677908602
Gauteng	2021	0.735426628	0.735426628	0.735426628
KwaZulu-Natal	2021	0.660483699	0.660483699	0.660483699
Limpopo	2021	0.612287836	0.612287836	0.612287836
Mpumalanga	2021	0.646057473	0.646057473	0.646057473
North-West	2021	0.652476432	0.652476432	0.652476432
Northern Cape	2021	0.664140622	0.664140622	0.664140622
Western Cape	2021	0.718532896	0.718532896	0.718532896
Sudan	2021	0.541949735	0.541949735	0.541949735
Alabama	2021	0.825605142	0.825605142	0.825605142
Alaska	2021	0.857524599	0.857524599	0.857524599
Arizona	2021	0.846732871	0.846732871	0.846732871
Arkansas	2021	0.81770902	0.81770902	0.81770902
California	2021	0.87021505	0.87021505	0.87021505
Colorado	2021	0.876654771	0.876654771	0.876654771
Connecticut	2021	0.903107563	0.903107563	0.903107563
Delaware	2021	0.866366234	0.866366234	0.866366234
District of Columbia	2021	0.905964755	0.905964755	0.905964755
Florida	2021	0.861979282	0.861979282	0.861979282
Georgia	2021	0.84656427	0.84656427	0.84656427
Hawaii	2021	0.869546917	0.869546917	0.869546917
Idaho	2021	0.83651792	0.83651792	0.83651792
Illinois	2021	0.879187797	0.879187797	0.879187797
Indiana	2021	0.842859834	0.842859834	0.842859834
Iowa	2021	0.86405652	0.86405652	0.86405652
Kansas	2021	0.859383805	0.859383805	0.859383805
Kentucky	2021	0.821325377	0.821325377	0.821325377
Louisiana	2021	0.825346457	0.825346457	0.825346457
Maine	2021	0.865754199	0.865754199	0.865754199
Maryland	2021	0.889225569	0.889225569	0.889225569
Massachusetts	2021	0.906621364	0.906621364	0.906621364
Michigan	2021	0.863971128	0.863971128	0.863971128
Minnesota	2021	0.887013945	0.887013945	0.887013945
Mississippi	2021	0.810471681	0.810471681	0.810471681
Missouri	2021	0.848169136	0.848169136	0.848169136

Montana	2021	0.859107705	0.859107705	0.859107705
Nebraska	2021	0.865128654	0.865128654	0.865128654
Nevada	2021	0.847682783	0.847682783	0.847682783
New Hampshire	2021	0.898263681	0.898263681	0.898263681
New Jersey	2021	0.891922443	0.891922443	0.891922443
New Mexico	2021	0.831716579	0.831716579	0.831716579
New York	2021	0.885176868	0.885176868	0.885176868
North Carolina	2021	0.844445313	0.844445313	0.844445313
North Dakota	2021	0.873031775	0.873031775	0.873031775
Ohio	2021	0.850513434	0.850513434	0.850513434
Oklahoma	2021	0.828663242	0.828663242	0.828663242
Oregon	2021	0.870177689	0.870177689	0.870177689
Pennsylvania	2021	0.872416752	0.872416752	0.872416752
Rhode Island	2021	0.883893485	0.883893485	0.883893485
South Carolina	2021	0.83771865	0.83771865	0.83771865
South Dakota	2021	0.855720868	0.855720868	0.855720868
Tennessee	2021	0.833033239	0.833033239	0.833033239
Texas	2021	0.834783278	0.834783278	0.834783278
Utah	2021	0.854616788	0.854616788	0.854616788
Vermont	2021	0.8899999	0.8899999	0.8899999
Virginia	2021	0.880119901	0.880119901	0.880119901
Washington	2021	0.877584845	0.877584845	0.877584845
West Virginia	2021	0.817713511	0.817713511	0.817713511
Wisconsin	2021	0.871726096	0.871726096	0.871726096
Wyoming	2021	0.862148722	0.862148722	0.862148722
North East England	2021	0.827273029	0.827273029	0.827273029
North West England	2021	0.844443823	0.844443823	0.844443823
Yorkshire and the Humber	2021	0.837459877	0.837459877	0.837459877
East Midlands	2021	0.835856049	0.835856049	0.835856049
West Midlands	2021	0.835865141	0.835865141	0.835865141
East of England	2021	0.856407925	0.856407925	0.856407925
Greater London	2021	0.904864758	0.904864758	0.904864758
South East England	2021	0.876066596	0.876066596	0.876066596
South West England	2021	0.861467015	0.861467015	0.861467015
Wales	2021	0.834278536	0.834278536	0.834278536
Aguascalientes	2021	0.681159284	0.681159284	0.681159284
Baja California	2021	0.704081972	0.704081972	0.704081972
Baja California Sur	2021	0.709694281	0.709694281	0.709694281
Campeche	2021	0.665363431	0.665363431	0.665363431
Coahuila	2021	0.677562875	0.677562875	0.677562875
Colima	2021	0.699116771	0.699116771	0.699116771
Chiapas	2021	0.569940909	0.569940909	0.569940909
Chihuahua	2021	0.673386571	0.673386571	0.673386571
Mexico City	2021	0.759848414	0.759848414	0.759848414
Durango	2021	0.639324841	0.639324841	0.639324841
Guanajuato	2021	0.646374308	0.646374308	0.646374308
Guerrero	2021	0.584850472	0.584850472	0.584850472

Hidalgo	2021	0.633478453	0.633478453	0.633478453
Jalisco	2021	0.675998373	0.675998373	0.675998373
México	2021	0.681587605	0.681587605	0.681587605
Michoacán de Ocampo	2021	0.613082024	0.613082024	0.613082024
Morelos	2021	0.670074046	0.670074046	0.670074046
Nayarit	2021	0.658178913	0.658178913	0.658178913
Nuevo León	2021	0.710141817	0.710141817	0.710141817
Oaxaca	2021	0.589041897	0.589041897	0.589041897
Puebla	2021	0.622031827	0.622031827	0.622031827
Querétaro	2021	0.682558115	0.682558115	0.682558115
Quintana Roo	2021	0.681068671	0.681068671	0.681068671
San Luis Potosí	2021	0.646651514	0.646651514	0.646651514
Sinaloa	2021	0.677164972	0.677164972	0.677164972
Sonora	2021	0.710679605	0.710679605	0.710679605
Tabasco	2021	0.648728024	0.648728024	0.648728024
Tamaulipas	2021	0.682086111	0.682086111	0.682086111
Tlaxcala	2021	0.64782243	0.64782243	0.64782243
Veracruz de Ignacio de la Llave	2021	0.628136965	0.628136965	0.628136965
Yucatán	2021	0.653050809	0.653050809	0.653050809
Zacatecas	2021	0.634773655	0.634773655	0.634773655
Aceh	2021	0.669551641	0.669551641	0.669551641
North Sumatra	2021	0.667025647	0.667025647	0.667025647
West Sumatra	2021	0.665763664	0.665763664	0.665763664
Riau	2021	0.722784516	0.722784516	0.722784516
Jambi	2021	0.639563676	0.639563676	0.639563676
South Sumatra	2021	0.645536761	0.645536761	0.645536761
Bengkulu	2021	0.613670654	0.613670654	0.613670654
Lampung	2021	0.608871357	0.608871357	0.608871357
Bangka-Belitung Islands	2021	0.64425964	0.64425964	0.64425964
Riau Islands	2021	0.747754306	0.747754306	0.747754306
North Kalimantan	2021	0.752986755	0.752986755	0.752986755
Jakarta	2021	0.799409443	0.799409443	0.799409443
West Java	2021	0.643676633	0.643676633	0.643676633
Central Java	2021	0.613388815	0.613388815	0.613388815
Yogyakarta	2021	0.675429917	0.675429917	0.675429917
East Java	2021	0.645517381	0.645517381	0.645517381
Banten	2021	0.640712993	0.640712993	0.640712993
Bali	2021	0.649585885	0.649585885	0.649585885
West Nusa Tenggara	2021	0.587034621	0.587034621	0.587034621
East Nusa Tenggara	2021	0.54945119	0.54945119	0.54945119
West Kalimantan	2021	0.586121672	0.586121672	0.586121672
Central Kalimantan	2021	0.638993825	0.638993825	0.638993825
South Kalimantan	2021	0.623206076	0.623206076	0.623206076
East Kalimantan	2021	0.759293869	0.759293869	0.759293869
North Sulawesi	2021	0.650468306	0.650468306	0.650468306
Central Sulawesi	2021	0.616253843	0.616253843	0.616253843
South Sulawesi	2021	0.621135414	0.621135414	0.621135414

Southeast Sulawesi	2021	0.615325868	0.615325868	0.615325868
Gorontalo	2021	0.569990208	0.569990208	0.569990208
West Sulawesi	2021	0.57565685	0.57565685	0.57565685
Maluku	2021	0.580321875	0.580321875	0.580321875
North Maluku	2021	0.562910873	0.562910873	0.562910873
West Papua	2021	0.675125644	0.675125644	0.675125644
Papua	2021	0.645802883	0.645802883	0.645802883
England	2021	0.861437309	0.861437309	0.861437309
Acre	2021	0.565851526	0.565851526	0.565851526
Alagoas	2021	0.535659976	0.535659976	0.535659976
Amazonas	2021	0.609484469	0.609484469	0.609484469
Amapá	2021	0.632179635	0.632179635	0.632179635
Bahia	2021	0.577394253	0.577394253	0.577394253
Ceará	2021	0.568665698	0.568665698	0.568665698
Distrito Federal	2021	0.776016643	0.776016643	0.776016643
Espírito Santo	2021	0.670192595	0.670192595	0.670192595
Goiás	2021	0.640933873	0.640933873	0.640933873
Maranhão	2021	0.49892987	0.49892987	0.49892987
Minas Gerais	2021	0.652293253	0.652293253	0.652293253
Mato Grosso do Sul	2021	0.64753332	0.64753332	0.64753332
Mato Grosso	2021	0.651185759	0.651185759	0.651185759
Pará	2021	0.582134405	0.582134405	0.582134405
Paraíba	2021	0.563868152	0.563868152	0.563868152
Paraná	2021	0.674856534	0.674856534	0.674856534
Pernambuco	2021	0.586764224	0.586764224	0.586764224
Piaui	2021	0.525243118	0.525243118	0.525243118
Rio de Janeiro	2021	0.716093842	0.716093842	0.716093842
Rio Grande do Norte	2021	0.589854818	0.589854818	0.589854818
Rondônia	2021	0.624854665	0.624854665	0.624854665
Roraima	2021	0.621005567	0.621005567	0.621005567
Rio Grande do Sul	2021	0.693337528	0.693337528	0.693337528
Santa Catarina	2021	0.701340701	0.701340701	0.701340701
Sergipe	2021	0.595840177	0.595840177	0.595840177
São Paulo	2021	0.713727597	0.713727597	0.713727597
Tocantins	2021	0.608587676	0.608587676	0.608587676
Andhra Pradesh	2021	0.551126806	0.551126806	0.551126806
Arunachal Pradesh	2021	0.58462714	0.58462714	0.58462714
Assam	2021	0.570474568	0.570474568	0.570474568
Bihar	2021	0.458456353	0.458456353	0.458456353
Chhattisgarh	2021	0.535088588	0.535088588	0.535088588
Delhi	2021	0.727201153	0.727201153	0.727201153
Goa	2021	0.723316494	0.723316494	0.723316494
Gujarat	2021	0.621447314	0.621447314	0.621447314
Haryana	2021	0.628418767	0.628418767	0.628418767
Himachal Pradesh	2021	0.639516914	0.639516914	0.639516914
Jammu & Kashmir and Ladakh	2021	0.608516912	0.608516912	0.608516912
Jharkhand	2021	0.523351361	0.523351361	0.523351361

Karnataka	2021	0.579087835	0.579087835	0.579087835
Kerala	2021	0.664801491	0.664801491	0.664801491
Madhya Pradesh	2021	0.527210683	0.527210683	0.527210683
Maharashtra	2021	0.635042824	0.635042824	0.635042824
Manipur	2021	0.587153831	0.587153831	0.587153831
Meghalaya	2021	0.564436059	0.564436059	0.564436059
Mizoram	2021	0.623903237	0.623903237	0.623903237
Nagaland	2021	0.639447051	0.639447051	0.639447051
Odisha	2021	0.550222878	0.550222878	0.550222878
Punjab	2021	0.632136354	0.632136354	0.632136354
Rajasthan	2021	0.532137284	0.532137284	0.532137284
Sikkim	2021	0.626560039	0.626560039	0.626560039
Tamil Nadu	2021	0.630219304	0.630219304	0.630219304
Telangana	2021	0.567956581	0.567956581	0.567956581
Tripura	2021	0.559154965	0.559154965	0.559154965
Uttar Pradesh	2021	0.52507026	0.52507026	0.52507026
Uttarakhand	2021	0.659725583	0.659725583	0.659725583
West Bengal	2021	0.554094043	0.554094043	0.554094043
Oslo	2021	0.945691791	0.945691791	0.945691791
Rogaland	2021	0.915083733	0.915083733	0.915083733
Møre og Romsdal	2021	0.907426289	0.907426289	0.907426289
Nordland	2021	0.896942278	0.896942278	0.896942278
Sweden except Stockholm	2021	0.87515115	0.87515115	0.87515115
Stockholm	2021	0.916622714	0.916622714	0.916622714
Hokkaidō	2021	0.843743975	0.843743975	0.843743975
Aomori	2021	0.828344206	0.828344206	0.828344206
Iwate	2021	0.834979691	0.834979691	0.834979691
Miyagi	2021	0.859886569	0.859886569	0.859886569
Akita	2021	0.832249015	0.832249015	0.832249015
Yamagata	2021	0.837933155	0.837933155	0.837933155
Fukushima	2021	0.840941474	0.840941474	0.840941474
Ibaraki	2021	0.85980457	0.85980457	0.85980457
Tochigi	2021	0.861186498	0.861186498	0.861186498
Gunma	2021	0.861111359	0.861111359	0.861111359
Saitama	2021	0.85637105	0.85637105	0.85637105
Chiba	2021	0.861292639	0.861292639	0.861292639
Tōkyō	2021	0.928810109	0.928810109	0.928810109
Kanagawa	2021	0.882654391	0.882654391	0.882654391
Niigata	2021	0.845152095	0.845152095	0.845152095
Toyama	2021	0.865181131	0.865181131	0.865181131
Ishikawa	2021	0.859378938	0.859378938	0.859378938
Fukui	2021	0.856142577	0.856142577	0.856142577
Yamanashi	2021	0.857782852	0.857782852	0.857782852
Nagano	2021	0.858577839	0.858577839	0.858577839
Gifu	2021	0.85334719	0.85334719	0.85334719
Shizuoka	2021	0.865450452	0.865450452	0.865450452
Aichi	2021	0.883048835	0.883048835	0.883048835

Mie	2021	0.860561189	0.860561189	0.860561189
Shiga	2021	0.874323368	0.874323368	0.874323368
Kyōto	2021	0.87601835	0.87601835	0.87601835
Ōsaka	2021	0.87640925	0.87640925	0.87640925
Hyōgo	2021	0.868170156	0.868170156	0.868170156
Nara	2021	0.851652195	0.851652195	0.851652195
Wakayama	2021	0.847037481	0.847037481	0.847037481
Tottori	2021	0.835956957	0.835956957	0.835956957
Shimane	2021	0.838672262	0.838672262	0.838672262
Okayama	2021	0.862560219	0.862560219	0.862560219
Hiroshima	2021	0.869908602	0.869908602	0.869908602
Yamaguchi	2021	0.856255885	0.856255885	0.856255885
Tokushima	2021	0.85748626	0.85748626	0.85748626
Kagawa	2021	0.85781836	0.85781836	0.85781836
Ehime	2021	0.843501706	0.843501706	0.843501706
Kōchi	2021	0.834906164	0.834906164	0.834906164
Fukuoka	2021	0.858347709	0.858347709	0.858347709
Saga	2021	0.834925899	0.834925899	0.834925899
Nagasaki	2021	0.829213881	0.829213881	0.829213881
Kumamoto	2021	0.834536592	0.834536592	0.834536592
Ōita	2021	0.849282438	0.849282438	0.849282438
Miyazaki	2021	0.826589859	0.826589859	0.826589859
Kagoshima	2021	0.832047307	0.832047307	0.832047307
Okinawa	2021	0.821803051	0.821803051	0.821803051
Baringo	2021	0.514883215	0.514883215	0.514883215
Bomet	2021	0.529367852	0.529367852	0.529367852
Bungoma	2021	0.49672518	0.49672518	0.49672518
Busia	2021	0.477665024	0.477665024	0.477665024
Elgeyo-Marakwet	2021	0.526297038	0.526297038	0.526297038
Embu	2021	0.547555396	0.547555396	0.547555396
Garissa	2021	0.321068269	0.321068269	0.321068269
Homa Bay	2021	0.493871805	0.493871805	0.493871805
Isiolo	2021	0.433966834	0.433966834	0.433966834
Kajiado	2021	0.506969707	0.506969707	0.506969707
Kakamega	2021	0.509528998	0.509528998	0.509528998
Kericho	2021	0.523573355	0.523573355	0.523573355
Kiambu	2021	0.593110152	0.593110152	0.593110152
Kilifi	2021	0.486009157	0.486009157	0.486009157
Kirinyaga	2021	0.54301445	0.54301445	0.54301445
Kisii	2021	0.544604844	0.544604844	0.544604844
Kisumu	2021	0.544409324	0.544409324	0.544409324
Kitui	2021	0.474749457	0.474749457	0.474749457
Kwale	2021	0.475138205	0.475138205	0.475138205
Laikipia	2021	0.575711773	0.575711773	0.575711773
Lamu	2021	0.503786492	0.503786492	0.503786492
Machakos	2021	0.548035791	0.548035791	0.548035791
Makueni	2021	0.512568943	0.512568943	0.512568943

Mandera	2021	0.238263833	0.238263833	0.238263833
Marsabit	2021	0.40018158	0.40018158	0.40018158
Meru	2021	0.508216146	0.508216146	0.508216146
Migori	2021	0.472803008	0.472803008	0.472803008
Mombasa	2021	0.596387778	0.596387778	0.596387778
Murang'a	2021	0.550869493	0.550869493	0.550869493
Nairobi	2021	0.683722442	0.683722442	0.683722442
Nakuru	2021	0.570045398	0.570045398	0.570045398
Nandi	2021	0.516279712	0.516279712	0.516279712
Narok	2021	0.459103072	0.459103072	0.459103072
Nyamira	2021	0.589267078	0.589267078	0.589267078
Nyandarua	2021	0.576109863	0.576109863	0.576109863
Nyeri	2021	0.578787484	0.578787484	0.578787484
Samburu	2021	0.378312395	0.378312395	0.378312395
Siaya	2021	0.476524395	0.476524395	0.476524395
Taita Taveta	2021	0.540980982	0.540980982	0.540980982
Tana River	2021	0.382845346	0.382845346	0.382845346
Tharaka Nithi	2021	0.529680112	0.529680112	0.529680112
Trans Nzoia	2021	0.54850455	0.54850455	0.54850455
Turkana	2021	0.373434245	0.373434245	0.373434245
Uasin Gishu	2021	0.567345589	0.567345589	0.567345589
Vihiga	2021	0.523089788	0.523089788	0.523089788
Wajir	2021	0.252050106	0.252050106	0.252050106
West Pokot	2021	0.444120899	0.444120899	0.444120899
Other Union Territories	2021	0.673439501	0.673439501	0.673439501
Darlington	2021	0.837204088	0.837204088	0.837204088
Northumberland	2021	0.823317013	0.823317013	0.823317013
Stockton-on-Tees	2021	0.83098489	0.83098489	0.83098489
Newcastle upon Tyne	2021	0.872520663	0.872520663	0.872520663
North Tyneside	2021	0.836400602	0.836400602	0.836400602
Redcar and Cleveland	2021	0.798369621	0.798369621	0.798369621
County Durham	2021	0.812276525	0.812276525	0.812276525
Gateshead	2021	0.830244183	0.830244183	0.830244183
Middlesbrough	2021	0.801470999	0.801470999	0.801470999
South Tyneside	2021	0.801073026	0.801073026	0.801073026
Sunderland	2021	0.819164125	0.819164125	0.819164125
Hartlepool	2021	0.798877773	0.798877773	0.798877773
Cheshire East	2021	0.884754634	0.884754634	0.884754634
Stockport	2021	0.861719713	0.861719713	0.861719713
Trafford	2021	0.896753779	0.896753779	0.896753779
Cheshire West and Chester	2021	0.871177933	0.871177933	0.871177933
Sefton	2021	0.825841895	0.825841895	0.825841895
Lancashire	2021	0.841125535	0.841125535	0.841125535
Cumbria	2021	0.843466901	0.843466901	0.843466901
Bolton	2021	0.814170764	0.814170764	0.814170764
Wirral	2021	0.819316291	0.819316291	0.819316291
Bury	2021	0.830089162	0.830089162	0.830089162

St Helens	2021	0.8122146	0.8122146	0.8122146
Warrington	2021	0.878756212	0.878756212	0.878756212
Oldham	2021	0.798408293	0.798408293	0.798408293
Rochdale	2021	0.802069322	0.802069322	0.802069322
Wigan	2021	0.80685214	0.80685214	0.80685214
Halton	2021	0.836181349	0.836181349	0.836181349
Liverpool	2021	0.848437635	0.848437635	0.848437635
Tameside	2021	0.800546694	0.800546694	0.800546694
Salford	2021	0.838699172	0.838699172	0.838699172
Blackburn with Darwen	2021	0.811503741	0.811503741	0.811503741
Knowsley	2021	0.813671711	0.813671711	0.813671711
Blackpool	2021	0.789925893	0.789925893	0.789925893
Manchester	2021	0.881429144	0.881429144	0.881429144
North Yorkshire	2021	0.85619466	0.85619466	0.85619466
East Riding of Yorkshire	2021	0.835731941	0.835731941	0.835731941
York	2021	0.888738932	0.888738932	0.888738932
North East Lincolnshire	2021	0.804837847	0.804837847	0.804837847
Calderdale	2021	0.837863928	0.837863928	0.837863928
North Lincolnshire	2021	0.825904867	0.825904867	0.825904867
Bradford	2021	0.815985897	0.815985897	0.815985897
Kirklees	2021	0.824207653	0.824207653	0.824207653
Leeds	2021	0.868761251	0.868761251	0.868761251
Sheffield	2021	0.854890971	0.854890971	0.854890971
Wakefield	2021	0.806811491	0.806811491	0.806811491
Rotherham	2021	0.804228811	0.804228811	0.804228811
Doncaster	2021	0.795614015	0.795614015	0.795614015
Kingston upon Hull, City of	2021	0.79993173	0.79993173	0.79993173
Barnsley	2021	0.789261821	0.789261821	0.789261821
Northamptonshire	2021	0.839635363	0.839635363	0.839635363
Leicestershire	2021	0.851888947	0.851888947	0.851888947
Lincolnshire	2021	0.821363757	0.821363757	0.821363757
Rutland	2021	0.852733752	0.852733752	0.852733752
Derby	2021	0.846010683	0.846010683	0.846010683
Derbyshire	2021	0.824238096	0.824238096	0.824238096
Nottinghamshire	2021	0.823007864	0.823007864	0.823007864
Nottingham	2021	0.85887136	0.85887136	0.85887136
Leicester	2021	0.8290671	0.8290671	0.8290671
Warwickshire	2021	0.866672661	0.866672661	0.866672661
Herefordshire, County of	2021	0.846310474	0.846310474	0.846310474
Solihull	2021	0.872079235	0.872079235	0.872079235
Shropshire	2021	0.843218762	0.843218762	0.843218762
Worcestershire	2021	0.843611304	0.843611304	0.843611304
Staffordshire	2021	0.829320238	0.829320238	0.829320238
Dudley	2021	0.804164522	0.804164522	0.804164522
Coventry	2021	0.848618069	0.848618069	0.848618069
Telford and Wrekin	2021	0.827341561	0.827341561	0.827341561
Stoke-on-Trent	2021	0.798242518	0.798242518	0.798242518

Walsall	2021	0.792529439	0.792529439	0.792529439
Wolverhampton	2021	0.812389843	0.812389843	0.812389843
Birmingham	2021	0.838066899	0.838066899	0.838066899
Sandwell	2021	0.795722297	0.795722297	0.795722297
Bedford	2021	0.858093661	0.858093661	0.858093661
Central Bedfordshire	2021	0.852254516	0.852254516	0.852254516
Suffolk	2021	0.841151028	0.841151028	0.841151028
Hertfordshire	2021	0.887510721	0.887510721	0.887510721
Essex	2021	0.845491449	0.845491449	0.845491449
Cambridgeshire	2021	0.888027582	0.888027582	0.888027582
Thurrock	2021	0.819597724	0.819597724	0.819597724
Norfolk	2021	0.837309678	0.837309678	0.837309678
Southend-on-Sea	2021	0.825801394	0.825801394	0.825801394
Peterborough	2021	0.837874469	0.837874469	0.837874469
Luton	2021	0.839717125	0.839717125	0.839717125
Richmond upon Thames	2021	0.932132548	0.932132548	0.932132548
Kensington and Chelsea	2021	0.946476699	0.946476699	0.946476699
Barnet	2021	0.88597606	0.88597606	0.88597606
Westminster	2021	0.937198627	0.937198627	0.937198627
Bromley	2021	0.869545327	0.869545327	0.869545327
Bexley	2021	0.844547626	0.844547626	0.844547626
Redbridge	2021	0.850265079	0.850265079	0.850265079
Merton	2021	0.887733957	0.887733957	0.887733957
Brent	2021	0.860164338	0.860164338	0.860164338
Hillingdon	2021	0.893138131	0.893138131	0.893138131
Havering	2021	0.834643869	0.834643869	0.834643869
Kingston upon Thames	2021	0.908698276	0.908698276	0.908698276
Sutton	2021	0.858421577	0.858421577	0.858421577
Harrow	2021	0.860015269	0.860015269	0.860015269
Enfield	2021	0.846461002	0.846461002	0.846461002
Croydon	2021	0.852066336	0.852066336	0.852066336
Hammersmith and Fulham	2021	0.935153171	0.935153171	0.935153171
Ealing	2021	0.88272888	0.88272888	0.88272888
Greenwich	2021	0.846080185	0.846080185	0.846080185
Wandsworth	2021	0.924241493	0.924241493	0.924241493
Waltham Forest	2021	0.840509602	0.840509602	0.840509602
Camden	2021	0.936474834	0.936474834	0.936474834
Lambeth	2021	0.916380817	0.916380817	0.916380817
Lewisham	2021	0.857308935	0.857308935	0.857308935
Hounslow	2021	0.896994582	0.896994582	0.896994582
Southwark	2021	0.919509828	0.919509828	0.919509828
Newham	2021	0.841688128	0.841688128	0.841688128
Barking and Dagenham	2021	0.807324954	0.807324954	0.807324954
Haringey	2021	0.872084552	0.872084552	0.872084552
Hackney	2021	0.892946278	0.892946278	0.892946278
Islington	2021	0.924740074	0.924740074	0.924740074
Tower Hamlets	2021	0.904488141	0.904488141	0.904488141

Wokingham	2021	0.911658755	0.911658755	0.911658755
Buckinghamshire	2021	0.888785243	0.888785243	0.888785243
Surrey	2021	0.904859403	0.904859403	0.904859403
Windsor and Maidenhead	2021	0.91581805	0.91581805	0.91581805
West Berkshire	2021	0.897757011	0.897757011	0.897757011
Hampshire	2021	0.87285	0.87285	0.87285
Bracknell Forest	2021	0.891002655	0.891002655	0.891002655
West Sussex	2021	0.864792147	0.864792147	0.864792147
Oxfordshire	2021	0.899475931	0.899475931	0.899475931
Reading	2021	0.905873593	0.905873593	0.905873593
Kent	2021	0.845345393	0.845345393	0.845345393
Brighton and Hove	2021	0.898257203	0.898257203	0.898257203
Medway	2021	0.820667842	0.820667842	0.820667842
East Sussex	2021	0.839594794	0.839594794	0.839594794
Portsmouth	2021	0.865875927	0.865875927	0.865875927
Isle of Wight	2021	0.826399628	0.826399628	0.826399628
Milton Keynes	2021	0.88717934	0.88717934	0.88717934
Southampton	2021	0.861349084	0.861349084	0.861349084
Slough	2021	0.878557248	0.878557248	0.878557248
South Gloucestershire	2021	0.884984764	0.884984764	0.884984764
Dorset	2021	0.851615144	0.851615144	0.851615144
Wiltshire	2021	0.859466242	0.859466242	0.859466242
North Somerset	2021	0.859017669	0.859017669	0.859017669
Devon	2021	0.855376103	0.855376103	0.855376103
Poole	2021	0.863494302	0.863494302	0.863494302
Bath and North East Somerset	2021	0.895435359	0.895435359	0.895435359
Gloucestershire	2021	0.871806302	0.871806302	0.871806302
Somerset	2021	0.843404311	0.843404311	0.843404311
Swindon	2021	0.867545906	0.867545906	0.867545906
Torbay	2021	0.813038477	0.813038477	0.813038477
Bristol, City of	2021	0.897041874	0.897041874	0.897041874
Bournemouth	2021	0.870464766	0.870464766	0.870464766
Cornwall	2021	0.839768559	0.839768559	0.839768559
Plymouth	2021	0.843307541	0.843307541	0.843307541
Tigray	2021	0.382183506	0.382183506	0.382183506
Afar	2021	0.28877341	0.28877341	0.28877341
Amhara	2021	0.320453189	0.320453189	0.320453189
Oromia	2021	0.335815016	0.335815016	0.335815016
Somali	2021	0.268253815	0.268253815	0.268253815
Benishangul-Gumuz	2021	0.320228483	0.320228483	0.320228483
Southern Nations, Nationalities, and Peoples with Sidama and South West Regions	2021	0.355521531	0.355521531	0.355521531
Harari	2021	0.538168759	0.538168759	0.538168759
Gambella	2021	0.458220084	0.458220084	0.458220084
Addis Ababa	2021	0.694729596	0.694729596	0.694729596
Dire Dawa	2021	0.540668646	0.540668646	0.540668646
Alborz	2021	0.748302022	0.748302022	0.748302022

Ardebil	2021	0.658639771	0.658639771	0.658639771
East Azarbayejan	2021	0.668193235	0.668193235	0.668193235
West Azarbayejan	2021	0.627112463	0.627112463	0.627112463
Bushehr	2021	0.708418708	0.708418708	0.708418708
Chahar Mahaal and Bakhtiari	2021	0.677988602	0.677988602	0.677988602
Fars	2021	0.714830769	0.714830769	0.714830769
Gilan	2021	0.712319447	0.712319447	0.712319447
Golestan	2021	0.65607753	0.65607753	0.65607753
Hamadan	2021	0.66673482	0.66673482	0.66673482
Hormozgan	2021	0.670331366	0.670331366	0.670331366
Ilam	2021	0.706194927	0.706194927	0.706194927
Isfahan	2021	0.709716845	0.709716845	0.709716845
Kerman	2021	0.668439265	0.668439265	0.668439265
Kermanshah	2021	0.674466468	0.674466468	0.674466468
North Khorasan	2021	0.651610197	0.651610197	0.651610197
Khorasan-e-Razavi	2021	0.669826641	0.669826641	0.669826641
South Khorasan	2021	0.653125383	0.653125383	0.653125383
Khuzestan	2021	0.669587913	0.669587913	0.669587913
Kohgiluyeh and Boyer-Ahmad	2021	0.693706198	0.693706198	0.693706198
Kurdistan	2021	0.642261692	0.642261692	0.642261692
Lorestan	2021	0.669118289	0.669118289	0.669118289
Markazi	2021	0.682843822	0.682843822	0.682843822
Mazandaran	2021	0.729929903	0.729929903	0.729929903
Qazvin	2021	0.6876536	0.6876536	0.6876536
Qom	2021	0.694075907	0.694075907	0.694075907
Semnan	2021	0.723926385	0.723926385	0.723926385
Sistan and Baluchistan	2021	0.551305977	0.551305977	0.551305977
Tehran	2021	0.776032454	0.776032454	0.776032454
Yazd	2021	0.713344284	0.713344284	0.713344284
Zanjan	2021	0.661729132	0.661729132	0.661729132
Trøndelag	2021	0.915977587	0.915977587	0.915977587
Azad Jammu & Kashmir	2021	0.54211877	0.54211877	0.54211877
Balochistan	2021	0.417971505	0.417971505	0.417971505
Gilgit-Baltistan	2021	0.397549987	0.397549987	0.397549987
Islamabad Capital Territory	2021	0.696195374	0.696195374	0.696195374
Khyber Pakhtunkhwa	2021	0.450581146	0.450581146	0.450581146
Punjab	2021	0.520200488	0.520200488	0.520200488
Sindh	2021	0.512574957	0.512574957	0.512574957
Vestland	2021	0.917596286	0.917596286	0.917596286
Agder	2021	0.907489539	0.907489539	0.907489539
Vestfold og Telemark	2021	0.907307419	0.907307419	0.907307419
Innlandet	2021	0.899754056	0.899754056	0.899754056
Viken	2021	0.914692607	0.914692607	0.914692607
Troms og Finnmark	2021	0.905163204	0.905163204	0.905163204

Table S2. Rate The lexis diagram of GBD data for the APC model

Period (median)	Age groups																	Birth cohort (median)
	0-4	5-9	10-14	15-19	20-24	25-29	30-34	35-39	40-44	45-49	50-54	55-59	60-64	65-69	70-74	75-79	80-84	
																	X	1908-1916 (1912)
																X	X	1913-1921 (1917)
															X	X	X	1918-1926 (1922)
														X	X	X	X	1923-1931 (1927)
													X	X	X	X	X	1928-1936 (1932)
												X	X	X	X	X	X	1933-1941 (1937)
											X	X	X	X	X	X		1938-1946 (1942)
										X	X	X	X	X	X			1943-1951 (1947)
									X	X	X	X	X	X				1948-1956 (1952)
								X	X	X	X	X	X					1953-1961 (1957)
							X	X	X	X	X	X						1958-1966 (1962)
						X	X	X	X	X	X							1963-1971 (1967)
					X	X	X	X	X	X								1968-1976 (1972)
				X	X	X	X	X	X									1973-1981 (1977)
			X	X	X	X	X	X										1978-1986 (1982)
		X	X	X	X	X	X											1983-1991 (1987)
	**X**	X	X	X	X	X												1988-1996 (1992)
1992-1996 (1994)	X	X	X	X	X													1993-2001 (1997)
1997-2001 (1999)	X	X	X	X														1998-2006 (2002)
2002-2006 (2004)	X	X	X															2003-2011 (2007)
2007-2011 (2009)	X	X																2008-2016 (2012)
2012-2016 (2014)	X																	2013-2021 (2017)
2017-2021 (2019)																		

GBD: global burden of diseases; APC: age-period-cohort

Table S3. DALYs cases and age-standardized rate for pulmonary arterial hypertension in 1990 and 2021, and estimated percentage change from 1990 to 2021, by 204 countries and 21 regions

Location	Number			Age-standardized Rate		
	1990	2021	Percent change	1990	2021	Percent change
Global	687419.3<br>(535240.8, 813086.3)	642104.3<br>(552272.7, 728993.2)	-0.07<br>(-0.27, 0.18)	13.2<br>(10.8, 15.4)	8.2<br>(7.1, 9.4)	-0.38<br>(-0.48, -0.25)
Andean Latin America	5049.0<br>(2977.3, 7421.9)	3543.8<br>(2795.1, 4490.0)	-0.30<br>(-0.51, 0.16)	11.9<br>(7.8, 16.4)	5.7<br>(4.5, 7.3)	-0.52<br>(-0.65, -0.26)
Bolivia (Plurinational State of)	1590.9<br>(717.7, 2702.4)	979.7<br>(707.6, 1392.2)	-0.38<br>(-0.61, 0.13)	19.5<br>(10.2, 31.5)	9.0<br>(6.6, 12.8)	-0.54<br>(-0.69, -0.23)
Ecuador	954.1<br>(801.8, 1233.6)	957.1<br>(811.9, 1115.6)	0.00<br>(-0.26, 0.29)	9.6<br>(7.8, 12.0)	5.8<br>(4.9, 6.7)	-0.39<br>(-0.54, -0.19)
Peru	2504.1<br>(1458.2, 3768.6)	1607.0<br>(1197.3, 2183.8)	-0.36<br>(-0.60, 0.18)	10.6<br>(6.9, 14.8)	4.6<br>(3.5, 6.3)	-0.56<br>(-0.71, -0.27)
Australasia	1514.2<br>(1337.1, 1856.3)	1433.5<br>(1305.4, 1562.9)	-0.05<br>(-0.25, 0.10)	7.4<br>(6.5, 9.1)	3.7<br>(3.4, 4.0)	-0.50<br>(-0.61, -0.42)
Australia	1322.3<br>(1150.7, 1671.8)	1207.1<br>(1087.4, 1333.5)	-0.09<br>(-0.30, 0.09)	7.8<br>(6.8, 9.9)	3.7<br>(3.4, 4.0)	-0.53<br>(-0.63, -0.44)
New Zealand	192.0<br>(177.7, 205.6)	226.4<br>(206.9, 249.3)	0.18<br>(0.07, 0.31)	5.3<br>(4.9, 5.7)	3.6<br>(3.3, 3.9)	-0.33<br>(-0.40, -0.26)
Caribbean	7398.3<br>(4169.8, 11145.3)	5071.2<br>(2987.6, 7877.3)	-0.31<br>(-0.48, -0.09)	19.7<br>(11.8, 28.8)	11.7<br>(6.5, 18.7)	-0.41<br>(-0.56, -0.22)
Antigua and Barbuda	2.5<br>(2.3, 2.9)	1.4<br>(1.3, 1.6)	-0.44<br>(-0.52, -0.35)	4.4<br>(3.9, 5.0)	1.5<br>(1.4, 1.6)	-0.66<br>(-0.71, -0.61)
Bahamas	105.0<br>(93.3, 118.7)	59.0<br>(46.7, 75)	-0.44<br>(-0.56, -0.27)	44.3<br>(39.2, 49.7)	14.8<br>(11.7, 18.6)	-0.67<br>(-0.74, -0.57)
Barbados	97.5<br>(84.1, 109.8)	43.1<br>(34.3, 54.0)	-0.56<br>(-0.66, -0.44)	38.0<br>(32.9, 42.5)	11.9<br>(9.3, 15.1)	-0.69<br>(-0.76, -0.60)
Belize	51.0<br>(45.2, 58.1)	22.3<br>(19.5, 25.1)	-0.56<br>(-0.64, -0.48)	23.4<br>(21.3, 26.3)	5.7<br>(5.0, 6.5)	-0.76<br>(-0.79, -0.72)

Bermuda	33.8<br>(29.5, 39.5)	15.6<br>(13.2, 18.9)	-0.54<br>(-0.64, -0.43)	55.5<br>(48.5, 64.7)	15.7<br>(13.1, 19.1)	-0.72<br>(-0.78, -0.65)
Cuba	483.7<br>(445.5, 534.3)	265.0<br>(227.5, 300.9)	-0.45<br>(-0.54, -0.36)	4.7<br>(4.3, 5.2)	1.9<br>(1.6, 2.1)	-0.59<br>(-0.66, -0.53)
Dominica	4.2<br>(2.6, 5.5)	2.3<br>(1.6, 3.8)	-0.46<br>(-0.64, -0.10)	6.1<br>(3.9, 8.2)	3.5<br>(2.5, 5.7)	-0.43<br>(-0.62, -0.06)
Dominican Republic	841.7<br>(527.6, 1156.4)	456.3<br>(329.6, 832.0)	-0.46<br>(-0.66, 0.19)	9.7<br>(7.0, 13.1)	4.3<br>(3.1, 7.9)	-0.56<br>(-0.71, -0.18)
Grenada	28.1<br>(24.1, 33.0)	9.3<br>(8.0, 10.7)	-0.67<br>(-0.74, -0.60)	32.9<br>(28.3, 38.7)	8.8<br>(7.7, 10.0)	-0.73<br>(-0.78, -0.68)
Guyana	78.7<br>(68.5, 91.8)	119.1<br>(90.6, 157.2)	0.51<br>(0.11, 1.00)	8.7<br>(7.7, 9.8)	16.1<br>(12.3, 21.1)	0.85<br>(0.38, 1.42)
Haiti	3922.9<br>(1303.4, 7297.1)	3454.1<br>(1446.5, 6073.1)	-0.12<br>(-0.41, 0.50)	45.5<br>(17.7, 78.7)	25.2<br>(11.9, 42.3)	-0.45<br>(-0.61, -0.19)
Jamaica	230.3<br>(199.6, 265.4)	78.1<br>(60.3, 100.4)	-0.66<br>(-0.75, -0.56)	9.7<br>(8.4, 11.0)	2.8<br>(2.2, 3.6)	-0.71<br>(-0.79, -0.62)
Puerto Rico	832.5<br>(772.6, 898.7)	158.5<br>(132.5, 185.0)	-0.81<br>(-0.84, -0.77)	23.7<br>(22.0, 25.6)	4.0<br>(3.3, 4.7)	-0.83<br>(-0.86, -0.80)
Saint Kitts and Nevis	3.2<br>(2.8, 4.5)	1.4<br>(1.1, 1.7)	-0.57<br>(-0.72, -0.43)	8.1<br>(7.1, 11.4)	2.3<br>(1.9, 2.8)	-0.71<br>(-0.81, -0.62)
Saint Lucia	34.5<br>(30.3, 38.9)	16.1<br>(13.2, 19.5)	-0.53<br>(-0.63, -0.43)	28.5<br>(25.2, 31.9)	8.4<br>(6.8, 10.2)	-0.71<br>(-0.77, -0.64)
Saint Vincent and the Grenadines	4.6<br>(4.0, 5.4)	2.8<br>(2.4, 3.3)	-0.39<br>(-0.50, -0.25)	4.3<br>(3.8, 4.9)	2.4<br>(2.1, 2.9)	-0.43<br>(-0.53, -0.30)
Suriname	142.5<br>(78.1, 178.3)	78.7<br>(58.5, 109.4)	-0.45<br>(-0.61, 0.22)	36.8<br>(21.4, 45.2)	14.3<br>(10.6, 19.6)	-0.61<br>(-0.73, -0.22)
Trinidad and Tobago	233.4<br>(209.4, 259.6)	110.4<br>(84.9, 141.8)	-0.53<br>(-0.65, -0.38)	20.9<br>(18.9, 23.3)	7.7<br>(5.9, 9.9)	-0.63<br>(-0.73, -0.51)
United States Virgin Islands	17.6<br>(10.2, 22.3)	6.1<br>(4.3, 8.6)	-0.66<br>(-0.77, -0.44)	17.0<br>(9.8, 21.3)	6.4<br>(4.5, 8.8)	-0.62<br>(-0.76, -0.35)
Central Asia	9071.2<br>(7195.1, 10645.6)	11618.5<br>(9513.9, 14201.8)	0.28<br>(0.02, 0.60)	14.1<br>(11.3, 16.5)	12.9<br>(10.6, 15.6)	-0.09<br>(-0.25, 0.13)

Armenia	255.9<br>(222.3, 297.7)	80.7<br>(67.2, 94.2)	-0.68<br>(-0.76, -0.61)	8.1<br>(7.0, 9.6)	2.2<br>(1.8, 2.5)	-0.73<br>(-0.79, -0.67)
Azerbaijan	1503.3<br>(949.2, 2126.4)	1871.0<br>(1077.8, 2999.1)	0.24<br>(-0.31, 1.21)	22.6<br>(14.6, 31.5)	17.6<br>(10.7, 27.7)	-0.22<br>(-0.52, 0.29)
Georgia	755.7<br>(585.3, 945.8)	1317.7<br>(1039.2, 1665.1)	0.74<br>(0.24, 1.47)	13.2<br>(10.3, 16.6)	27.8<br>(22.2, 34.8)	1.11<br>(0.51, 1.98)
Kazakhstan	263.9<br>(217.1, 310.5)	268.0<br>(220.2, 316.0)	0.02<br>(-0.22, 0.35)	1.7<br>(1.4, 2.1)	1.5<br>(1.2, 1.7)	-0.15<br>(-0.34, 0.14)
Kyrgyzstan	137.8<br>(115.7, 164.2)	105.3<br>(86.1, 130.9)	-0.24<br>(-0.43, 0.02)	3.5<br>(2.9, 4.2)	1.8<br>(1.5, 2.2)	-0.49<br>(-0.62, -0.33)
Mongolia	801.2<br>(407.1, 1219.5)	1230.3<br>(721.5, 1613.7)	0.54<br>(0.01, 1.37)	50.7<br>(26.3, 79.6)	43.9<br>(25.6, 56.5)	-0.13<br>(-0.43, 0.37)
Tajikistan	1915.9<br>(1192.8, 2736.0)	2491.5<br>(1593.1, 3452.3)	0.30<br>(-0.09, 1.00)	39.7<br>(24.2, 53.5)	27.4<br>(17.7, 37.4)	-0.31<br>(-0.52, 0.02)
Turkmenistan	196.4<br>(163.0, 245.1)	379.7<br>(289.5, 499.3)	0.93<br>(0.42, 1.69)	6.3<br>(5.0, 7.8)	7.9<br>(6.0, 10.3)	0.25<br>(-0.10, 0.78)
Uzbekistan	3241.1<br>(2715.8, 3827.0)	3874.2<br>(3132.7, 4698.7)	0.20<br>(-0.08, 0.56)	17.1<br>(14.1, 20.6)	12.2<br>(9.8, 14.8)	-0.29<br>(-0.46, -0.07)
Central Europe	11026.3<br>(9783.7, 12154.1)	10423.5<br>(9511.8, 11459.4)	-0.05<br>(-0.18, 0.10)	8.2<br>(7.3, 8.9)	6.1<br>(5.5, 6.7)	-0.26<br>(-0.35, -0.14)
Albania	458.2<br>(290.8, 580.7)	286.5<br>(173.0, 512.1)	-0.37<br>(-0.62, 0.31)	16.1<br>(10.6, 20.3)	8.4<br>(5.3, 14.2)	-0.48<br>(-0.67, -0.04)
Bosnia and Herzegovina	343.6<br>(244.5, 458.7)	295.1<br>(212.4, 381.1)	-0.14<br>(-0.40, 0.27)	7.9<br>(5.7, 10.6)	5.9<br>(4.2, 7.7)	-0.25<br>(-0.48, 0.10)
Bulgaria	485.2<br>(412.1, 564.9)	286.0<br>(216.8, 375.6)	-0.41<br>(-0.58, -0.18)	4.9<br>(4.1, 5.7)	2.9<br>(2.2, 3.7)	-0.41<br>(-0.58, -0.18)
Croatia	191.2<br>(168.8, 218.5)	92.3<br>(78.2, 108.4)	-0.52<br>(-0.59, -0.42)	3.5<br>(3.1, 3.9)	1.3<br>(1.1, 1.5)	-0.62<br>(-0.68, -0.55)
Czechia	1445.0<br>(1215.5, 1725.1)	1898.9<br>(1608.4, 2169.6)	0.31<br>(0.05, 0.60)	12.0<br>(10.0, 14.4)	11.1<br>(9.3, 12.9)	-0.08<br>(-0.27, 0.14)
Hungary	1268.4<br>(1112.8, 1450.5)	725.0<br>(590.1, 908.0)	-0.43<br>(-0.57, -0.29)	10.1<br>(8.9, 11.6)	4.7<br>(3.8, 5.8)	-0.54<br>(-0.65, -0.42)

Montenegro	9.6<br>(5.4, 40.6)	8.4<br>(4.9, 38.2)	-0.12<br>(-0.38, 0.24)	1.5<br>(0.9, 6.8)	1.1<br>(0.6, 4.8)	-0.32<br>(-0.52, -0.05)
North Macedonia	89.4<br>(64.5, 133.3)	89.8<br>(46.7, 153.4)	0.00<br>(-0.43, 0.49)	4.7<br>(3.4, 7.3)	3.2<br>(1.7, 5.8)	-0.33<br>(-0.62, -0.02)
Poland	2434.7<br>(2143.5, 2770.4)	2111.9<br>(1911.6, 2297.0)	-0.13<br>(-0.27, 0.02)	6.0<br>(5.3, 6.9)	3.9<br>(3.6, 4.3)	-0.35<br>(-0.45, -0.23)
Romania	3535.9<br>(2924.5, 4195.8)	3948.6<br>(3383.7, 4490.6)	0.12<br>(-0.10, 0.43)	14.2<br>(11.8, 16.7)	13.9<br>(12.0, 15.8)	-0.02<br>(-0.20, 0.24)
Serbia	368.8<br>(261.8, 789.0)	337.0<br>(214.5, 618.1)	-0.09<br>(-0.35, 0.32)	3.8<br>(2.6, 8.7)	2.4<br>(1.6, 4.6)	-0.35<br>(-0.54, -0.06)
Slovakia	190.0<br>(129.4, 326.0)	154.0<br>(99.9, 267.8)	-0.19<br>(-0.42, 0.19)	3.4<br>(2.3, 6.1)	2.0<br>(1.3, 3.6)	-0.40<br>(-0.57, -0.12)
Slovenia	29.8<br>(26.0, 34.1)	38.2<br>(32.8, 44.4)	0.28<br>(0.08, 0.51)	1.3<br>(1.1, 1.5)	1.0<br>(0.9, 1.2)	-0.20<br>(-0.33, -0.06)
Central Latin America	9966.2<br>(8843.6, 11854.8)	7245.5<br>(6390.8, 8406.7)	-0.27<br>(-0.42, -0.14)	6.1<br>(5.4, 7.2)	3.0<br>(2.6, 3.5)	-0.51<br>(-0.61, -0.41)
Colombia	1787.0<br>(1488, 2250.0)	1644.9<br>(1380.2, 1948.8)	-0.08<br>(-0.33, 0.21)	5.8<br>(4.8, 7.3)	3.3<br>(2.7, 3.9)	-0.43<br>(-0.60, -0.25)
Costa Rica	311.1<br>(289.4, 335.2)	163.6<br>(145.0, 182.9)	-0.47<br>(-0.54, -0.40)	12.4<br>(11.5, 13.3)	3.3<br>(2.9, 3.7)	-0.73<br>(-0.77, -0.69)
El Salvador	677.4<br>(421.8, 882.1)	262.8<br>(190.2, 440.5)	-0.61<br>(-0.76, -0.17)	11.9<br>(8.0, 15.0)	4.2<br>(3.0, 7.0)	-0.65<br>(-0.77, -0.32)
Guatemala	863.2<br>(757.4, 1064.1)	324.3<br>(275.5, 385.5)	-0.62<br>(-0.72, -0.53)	9.4<br>(8.3, 11.1)	2.4<br>(2.0, 2.8)	-0.75<br>(-0.81, -0.69)
Honduras	338.4<br>(214.7, 467.3)	408.6<br>(282.6, 617.0)	0.21<br>(-0.14, 0.92)	8.2<br>(5.2, 12.0)	5.3<br>(3.7, 8.0)	-0.35<br>(-0.55, -0.09)
Mexico	4819.1<br>(4094.7, 5870.8)	3758.6<br>(3349.3, 4225.8)	-0.22<br>(-0.39, -0.07)	5.6<br>(5.0, 6.7)	3.1<br>(2.8, 3.5)	-0.44<br>(-0.55, -0.35)
Nicaragua	278.7<br>(143.1, 397.2)	102.5<br>(68.9, 301.8)	-0.63<br>(-0.8, 0.09)	6.1<br>(3.4, 9.4)	1.8<br>(1.2, 5.3)	-0.71<br>(-0.83, -0.31)
Panama	132.4<br>(114.2, 149.7)	88.7<br>(73.6, 104.2)	-0.33<br>(-0.47, -0.17)	6.3<br>(5.4, 7.0)	2.1<br>(1.7, 2.5)	-0.67<br>(-0.74, -0.59)

Venezuela (Bolivarian Republic of)	758.8<br>(656.8, 866.1)	491.6<br>(391.4, 609.3)	-0.35<br>(-0.50, -0.17)	4.2<br>(3.7, 4.8)	1.8<br>(1.5, 2.3)	-0.57<br>(-0.67, -0.46)
Central Sub-Saharan Africa	5367.3<br>(3258.0, 10524.7)	6524.0<br>(3586.2, 11025.2)	0.22<br>(-0.31, 1.04)	9.1<br>(5.6, 17.1)	6.2<br>(2.9, 11.1)	-0.32<br>(-0.55, -0.07)
Angola	1120.0<br>(596.5, 2654.9)	1628.1<br>(975.7, 2923.8)	0.45<br>(-0.19, 1.74)	10.1<br>(6.1, 21.3)	6.2<br>(3.3, 11.6)	-0.39<br>(-0.61, -0.03)
Central African Republic	324.1<br>(176.8, 698.3)	429.1<br>(231.8, 780.1)	0.32<br>(-0.18, 1.12)	11.4<br>(6.3, 21.8)	8.9<br>(4.3, 15.9)	-0.22<br>(-0.44, 0.09)
Congo	173.0<br>(114.0, 371.9)	205.5<br>(130.7, 368.6)	0.19<br>(-0.21, 0.89)	7.9<br>(5.3, 16.2)	4.8<br>(2.9, 8.8)	-0.39<br>(-0.55, -0.16)
Democratic Republic of the Congo	3648.2<br>(2260.0, 6621.2)	4158.0<br>(2013.5, 7024.2)	0.14<br>(-0.44, 0.97)	8.8<br>(5.3, 15.9)	6.1<br>(2.7, 11.3)	-0.30<br>(-0.58, -0.01)
Equatorial Guinea	41.3<br>(22.5, 82.5)	43.2<br>(24.3, 74.7)	0.05<br>(-0.39, 1.22)	9.5<br>(5.5, 17.8)	3.8<br>(2.1, 6.7)	-0.60<br>(-0.75, -0.28)
Gabon	60.7<br>(38.8, 107.6)	60.1<br>(34.0, 104.2)	-0.01<br>(-0.36, 0.57)	7.0<br>(4.1, 13.0)	4.1<br>(2.3, 7.1)	-0.41<br>(-0.58, -0.11)
East Asia	151596.3<br>(117393.6, 205773.2)	154740.4<br>(102939, 190399.5)	0.02<br>(-0.37, 0.47)	15.8<br>(12.3, 21.2)	8.8<br>(6.0, 11.0)	-0.44<br>(-0.65, -0.20)
China mainland	149699.4<br>(115904.3, 202545.9)	150940.7<br>(99583.3, 186503.4)	0.01<br>(-0.38, 0.46)	16.2<br>(12.6, 21.6)	8.9<br>(6.0, 11.1)	-0.45<br>(-0.66, -0.21)
Democratic People's Republic of Korea	1491.8<br>(1013.6, 2585.8)	2285.0<br>(1613.4, 3818.2)	0.53<br>(0.07, 1.23)	8.5<br>(5.8, 14.6)	8.0<br>(5.7, 13.4)	-0.05<br>(-0.34, 0.36)
Taiwan (Province of China mainland)	405.1<br>(382.3, 431.6)	1514.8<br>(1353.0, 1649)	2.74<br>(2.33, 3.13)	2.4<br>(2.3, 2.5)	4.2<br>(3.8, 4.5)	0.74<br>(0.58, 0.9)
Eastern Europe	20628.2<br>(18780.6, 23859.5)	8356.5<br>(7760.4, 8996.0)	-0.59<br>(-0.66, -0.54)	9.2<br>(8.5, 10.5)	3.3<br>(3.1, 3.6)	-0.64<br>(-0.69, -0.59)
Belarus	221.8<br>(185.5, 282.1)	185.5<br>(153.8, 219.5)	-0.16<br>(-0.37, 0.03)	2.2<br>(1.9, 2.9)	1.6<br>(1.4, 1.9)	-0.27<br>(-0.47, -0.09)
Estonia	18.9<br>(16.3, 22.3)	24.4<br>(20.9, 28.4)	0.29<br>(0.10, 0.49)	1.2<br>(1.0, 1.4)	1.1<br>(1.0, 1.3)	-0.04<br>(-0.16, 0.09)
Latvia	32.9<br>(28.5, 38.7)	89.1<br>(76.7, 101.8)	1.71<br>(1.2, 2.31)	1.2<br>(1.0, 1.4)	2.9<br>(2.5, 3.3)	1.47<br>(1.05, 1.99)

Lithuania	63.1<br>(55.2, 72.2)	99.6<br>(87.3, 113.5)	0.58<br>(0.34, 0.87)	1.7<br>(1.5, 1.9)	2.3<br>(2.0, 2.6)	0.34<br>(0.15, 0.57)
Republic of Moldova	17.3<br>(12.8, 23.2)	22.2<br>(17.5, 28.7)	0.29<br>(0.17, 0.47)	0.4<br>(0.3, 0.5)	0.5<br>(0.4, 0.7)	0.39<br>(0.23, 0.63)
Russian Federation	18577.2<br>(16746.9, 21770.6)	6389.1<br>(5934.4, 6897.3)	-0.66<br>(-0.71, -0.60)	12.7<br>(11.5, 14.6)	3.6<br>(3.4, 3.9)	-0.71<br>(-0.76, -0.67)
Ukraine	1697.2<br>(1533.5, 1901.4)	1546.7<br>(1172.4, 1956.1)	-0.09<br>(-0.32, 0.18)	3.0<br>(2.7, 3.4)	3.1<br>(2.4, 3.8)	0.02<br>(-0.24, 0.30)
Eastern Sub-Saharan Africa	23147.7<br>(14694.4, 45765.3)	26604.8<br>(14265.1, 49615.5)	0.15<br>(-0.25, 0.69)	11.1<br>(6.2, 21.2)	6.9<br>(3.3, 12.6)	-0.38<br>(-0.53, -0.19)
Burundi	981.1<br>(591.8, 1970.9)	916.2<br>(413.9, 1813.5)	-0.07<br>(-0.52, 0.60)	15.6<br>(8.3, 32.7)	7.6<br>(3.1, 15.1)	-0.51<br>(-0.74, -0.20)
Comoros	62.2<br>(35.3, 107.9)	41.3<br>(22.7, 80.7)	-0.34<br>(-0.59, 0.10)	11.7<br>(6.6, 19.7)	5.9<br>(3.2, 11.4)	-0.50<br>(-0.65, -0.23)
Djibouti	39.0<br>(19.5, 78.4)	62.6<br>(30.5, 119.1)	0.61<br>(-0.12, 2.03)	9.3<br>(5.1, 18.3)	5.7<br>(2.7, 10.8)	-0.39<br>(-0.61, 0.01)
Eritrea	459.7<br>(255.6, 966.8)	469.8<br>(239.3, 930.6)	0.02<br>(-0.35, 0.64)	13.1<br>(7.1, 27.0)	8.0<br>(3.8, 15.2)	-0.39<br>(-0.57, -0.09)
Ethiopia	5663.0<br>(3177.0, 11303.8)	5398.5<br>(2500.4, 11568.9)	-0.05<br>(-0.43, 0.59)	10.8<br>(5.3, 20.8)	5.5<br>(2.3, 11.2)	-0.49<br>(-0.65, -0.23)
Kenya	1163.9<br>(747.7, 2247.6)	1654.8<br>(819.9, 3203.8)	0.42<br>(-0.02, 1.10)	5.2<br>(2.6, 10.7)	4.1<br>(1.9, 7.8)	-0.22<br>(-0.38, 0.09)
Madagascar	2391.3<br>(1395.4, 5242.2)	3453.9<br>(1961.4, 6744.7)	0.44<br>(-0.04, 1.11)	18.0<br>(10.8, 35.4)	12.8<br>(6.9, 24.5)	-0.29<br>(-0.48, -0.06)
Malawi	1164.5<br>(640.3, 2318.9)	1045.5<br>(546.0, 1827.2)	-0.10<br>(-0.50, 0.55)	9.5<br>(5.7, 17.0)	6.2<br>(3.1, 10.6)	-0.35<br>(-0.56, -0.06)
Mozambique	1573.1<br>(837.0, 2789.0)	2156.9<br>(1087.1, 3608.3)	0.37<br>(-0.21, 1.40)	10.8<br>(5.9, 20.2)	8.6<br>(3.8, 15.2)	-0.20<br>(-0.45, 0.20)
Rwanda	1150.1<br>(686, 2621.7)	725.4<br>(355.6, 1528.5)	-0.37<br>(-0.67, 0.19)	14.9<br>(8.5, 30.8)	6.2<br>(2.9, 13.1)	-0.59<br>(-0.76, -0.31)
Somalia	1328.4<br>(604.4, 2411.9)	2163.2<br>(667.2, 4512.5)	0.63<br>(-0.17, 1.52)	15.2<br>(6.0, 29.7)	10.6<br>(2.8, 23.0)	-0.31<br>(-0.61, 0.02)

South Sudan	973.5<br>(440.0, 1977.3)	1105.8<br>(627.7, 2079.5)	0.14<br>(-0.29, 1.07)	13.9<br>(7.6, 26.6)	10.3<br>(5.3, 18.6)	-0.25<br>(-0.51, 0.21)
Uganda	2518.0<br>(1508.9, 4482.5)	2530.9<br>(1529.5, 4433.2)	0.01<br>(-0.31, 0.56)	11.7<br>(5.8, 22.8)	6.0<br>(3.2, 10.4)	-0.49<br>(-0.67, -0.20)
United Republic of Tanzania	2891.9<br>(1921.5, 5623.6)	3514.3<br>(2011.8, 6433.4)	0.22<br>(-0.22, 0.90)	10.2<br>(5.6, 18.8)	6.3<br>(3.3, 11.4)	-0.39<br>(-0.56, -0.14)
Zambia	771.3<br>(449.8, 1776.9)	1342.6<br>(566.7, 2580.9)	0.74<br>(-0.29, 2.58)	9.2<br>(5.5, 18.2)	8.6<br>(3.2, 17.2)	-0.06<br>(-0.58, 0.65)
High-income Asia Pacific	18474.3<br>(17689.1, 19467.1)	19988.4<br>(17441.9, 21997.2)	0.08<br>(-0.05, 0.20)	12.0<br>(11.4, 12.8)	8.3<br>(7.7, 8.9)	-0.31<br>(-0.37, -0.25)
Brunei Darussalam	53.9<br>(28.2, 72.2)	45.4<br>(26.8, 58.3)	-0.16<br>(-0.38, 0.31)	20.6<br>(11.3, 27.6)	11.4<br>(7.2, 14.9)	-0.45<br>(-0.59, -0.18)
Japan	15633.9<br>(15119.3, 16189.6)	18490.0<br>(16064.3, 20369.6)	0.18<br>(0.05, 0.29)	14.1<br>(13.7, 14.6)	10.7<br>(10.0, 11.5)	-0.24<br>(-0.28, -0.19)
Republic of Korea	2475.3<br>(1691.9, 3462.4)	1245.7<br>(841.8, 2906.4)	-0.50<br>(-0.71, -0.04)	6.5<br>(4.5, 9.5)	2.4<br>(1.7, 4.7)	-0.63<br>(-0.79, -0.36)
Singapore	311.2<br>(289.1, 335.1)	207.2<br>(187.2, 227.8)	-0.33<br>(-0.41, -0.25)	11.6<br>(10.8, 12.5)	3.8<br>(3.4, 4.3)	-0.67<br>(-0.71, -0.63)
High-income North America	30206.1<br>(27736.1, 32714.7)	38372.6<br>(35059.6, 40844.4)	0.27<br>(0.15, 0.38)	10.1<br>(9.4, 11)	7.7<br>(7.2, 8.2)	-0.24<br>(-0.31, -0.18)
Canada	3066.0<br>(2872.1, 3264.3)	2170.6<br>(1981.6, 2336.9)	-0.29<br>(-0.36, -0.22)	11.4<br>(10.6, 12.1)	4.6<br>(4.2, 5.0)	-0.59<br>(-0.63, -0.55)
Greenland	16.0<br>(6.5, 23)	3.8<br>(2.8, 5.6)	-0.76<br>(-0.87, -0.25)	31.6<br>(13.6, 44.1)	7.1<br>(5.1, 10.2)	-0.78<br>(-0.88, -0.35)
United States of America	27123.4<br>(24675.1, 29590.1)	36197.6<br>(33003.8, 38647.8)	0.33<br>(0.20, 0.46)	10.0<br>(9.2, 10.9)	8.1<br>(7.5, 8.6)	-0.19<br>(-0.28, -0.12)
North Africa and Middle East	145728.1<br>(74878.6, 204070.2)	80752.7<br>(58085.8, 98810.3)	-0.45<br>(-0.67, -0.10)	35.8<br>(21.2, 46.1)	14.8<br>(10.8, 18.0)	-0.59<br>(-0.73, -0.40)
Afghanistan	2360.2<br>(1185.7, 3637.1)	8110.1<br>(3767.6, 12130.9)	2.44<br>(0.49, 4.63)	18.2<br>(9.2, 29.2)	25.3<br>(11.0, 39.0)	0.39<br>(-0.43, 1.26)
Algeria	3349.5<br>(2116.1, 5772.9)	4335.0<br>(2024.2, 6496.2)	0.29<br>(-0.58, 1.23)	12.3<br>(8.0, 22.0)	11.1<br>(4.9, 16.6)	-0.10<br>(-0.68, 0.50)

Bahrain	28.8<br>(20.9, 56.2)	48.2<br>(24.2, 83.0)	0.67<br>(-0.19, 1.55)	6.6<br>(4.9, 14.2)	4.5<br>(2.3, 8.0)	-0.32<br>(-0.65, 0.02)
Egypt	58614.9<br>(23940.4, 86262.2)	17675.6<br>(13635.2, 23380.9)	-0.70<br>(-0.81, -0.31)	74.2<br>(37.6, 105.4)	16.5<br>(12.8, 21.9)	-0.78<br>(-0.86, -0.54)
Iran (Islamic Republic of)	28134.5<br>(17154.6, 38911.2)	12800.8<br>(9989.3, 14671.9)	-0.55<br>(-0.70, -0.27)	50.9<br>(32.6, 63.6)	16.3<br>(12.9, 18.7)	-0.68<br>(-0.76, -0.51)
Iraq	2475.6<br>(1290.6, 3561.6)	1822.0<br>(992.5, 3157.5)	-0.26<br>(-0.52, 0.27)	11.1<br>(6.2, 16.5)	5.2<br>(2.8, 10.1)	-0.53<br>(-0.69, -0.25)
Jordan	122.2<br>(67.0, 443.7)	253.3<br>(124.4, 686.3)	1.07<br>(-0.21, 2.87)	2.8<br>(1.5, 10.9)	2.5<br>(1.2, 6.9)	-0.11<br>(-0.64, 0.65)
Kuwait	68.5<br>(60.8, 77.5)	206.9<br>(174.1, 243.4)	2.02<br>(1.43, 2.70)	4.2<br>(3.7, 4.7)	6.5<br>(5.4, 7.7)	0.56<br>(0.27, 0.91)
Lebanon	947.7<br>(528.7, 1451.3)	617.4<br>(485.4, 814.0)	-0.35<br>(-0.60, 0.28)	32.0<br>(19.0, 48.2)	11.3<br>(8.9, 14.5)	-0.65<br>(-0.78, -0.37)
Libya	1145.6<br>(646.7, 2165.3)	1003.6<br>(437.2, 1781.9)	-0.12<br>(-0.63, 0.50)	20.3<br>(12.0, 37.9)	20.4<br>(8.7, 36.6)	0.01<br>(-0.57, 0.78)
Morocco	4665.2<br>(2537.1, 7926.9)	4558.9<br>(2226.5, 6953.3)	-0.02<br>(-0.68, 0.71)	15.6<br>(8.7, 26.7)	13.5<br>(6.5, 20.5)	-0.14<br>(-0.69, 0.45)
Oman	86.5<br>(46.1, 226.4)	133.7<br>(51.1, 241.3)	0.55<br>(-0.55, 2.41)	4.2<br>(2.2, 12.1)	3.9<br>(1.5, 7.2)	-0.07<br>(-0.70, 1.08)
Palestine	293.4<br>(130.9, 482.4)	212.2<br>(91.0, 499.8)	-0.28<br>(-0.57, 0.29)	10.2<br>(4.9, 19.4)	4.7<br>(2.0, 11.9)	-0.54<br>(-0.70, -0.29)
Qatar	18.6<br>(12.7, 36.6)	68.7<br>(37.3, 102.1)	2.69<br>(0.38, 5.21)	5.3<br>(3.7, 10.7)	3.7<br>(2.0, 5.6)	-0.30<br>(-0.73, 0.16)
Saudi Arabia	952.4<br>(572.9, 1680.7)	871.8<br>(464.9, 1517.9)	-0.08<br>(-0.50, 0.62)	5.7<br>(3.6, 11.8)	2.6<br>(1.4, 5.4)	-0.54<br>(-0.74, -0.22)
Sudan	5213.5<br>(2386.8, 10407.3)	7551.2<br>(3740.1, 11346.4)	0.45<br>(-0.46, 1.90)	18.2<br>(9.4, 32.7)	17.3<br>(8.6, 25.6)	-0.05<br>(-0.64, 0.71)
Syrian Arab Republic	731.5<br>(373.2, 2389.9)	283.9<br>(132.9, 1389.6)	-0.61<br>(-0.83, -0.31)	4.5<br>(2.5, 16.6)	2.3<br>(1.1, 11.6)	-0.49<br>(-0.76, -0.14)
Tunisia	901.2<br>(574.3, 1529.3)	1063.5<br>(438.1, 1720.9)	0.18<br>(-0.64, 1.33)	10.6<br>(6.8, 18.9)	9.2<br>(3.9, 14.8)	-0.13<br>(-0.70, 0.68)

Türkiye	31948.7<br>(16439.4, 49529.9)	12652.4<br>(9855.7, 15875.3)	-0.60<br>(-0.75, -0.16)	53.9<br>(30.7, 77.5)	16.6<br>(12.9, 20.6)	-0.69<br>(-0.79, -0.43)
United Arab Emirates	418.4<br>(231.8, 631.7)	433.7<br>(265.8, 540.8)	0.04<br>(-0.31, 0.67)	26.8<br>(14.5, 38.9)	10.3<br>(5.6, 13.0)	-0.62<br>(-0.73, -0.39)
Yemen	3171.4<br>(1562.8, 5312.9)	5974.8<br>(2916.8, 8721.0)	0.88<br>(-0.26, 2.11)	16.7<br>(8.2, 28.0)	19.3<br>(9.1, 28.9)	0.15<br>(-0.52, 0.82)
Oceania	704.5<br>(448.5, 1090.1)	1454.6<br>(988.2, 2410.5)	1.06<br>(0.54, 1.79)	10.8<br>(7.0, 17.6)	10.1<br>(6.9, 17.1)	-0.07<br>(-0.27, 0.20)
American Samoa	1.9<br>(1.4, 3.4)	1.9<br>(1.1, 3.7)	0.00<br>(-0.33, 0.40)	5.1<br>(3.6, 10.0)	4.2<br>(2.4, 8.1)	-0.19<br>(-0.44, 0.10)
Cook Islands	1.7<br>(1.3, 3.0)	0.9<br>(0.6, 2.0)	-0.45<br>(-0.67, -0.14)	9.8<br>(7.3, 17.6)	5.3<br>(3.1, 10.9)	-0.46<br>(-0.68, -0.13)
Fiji	43.6<br>(27.8, 78.1)	43.8<br>(24.6, 83.1)	0.00<br>(-0.31, 0.39)	7.0<br>(4.5, 13.5)	5.2<br>(2.9, 10.0)	-0.26<br>(-0.48, 0.02)
Guam	5.2<br>(3.8, 9.7)	4.2<br>(2.3, 8.2)	-0.19<br>(-0.46, 0.11)	4.5<br>(3.3, 8.9)	2.5<br>(1.4, 4.9)	-0.44<br>(-0.61, -0.24)
Kiribati	6.6<br>(3.6, 13.4)	8.3<br>(4.9, 16.1)	0.26<br>(-0.05, 0.76)	9.8<br>(5.5, 19.6)	8.1<br>(4.9, 15.8)	-0.18<br>(-0.38, 0.12)
Marshall Islands	3.4<br>(2.1, 5.8)	3.9<br>(2.3, 7.5)	0.17<br>(-0.25, 0.71)	10.4<br>(6.5, 19.5)	8.0<br>(4.7, 15.8)	-0.23<br>(-0.47, 0.08)
Micronesia (Federated States of)	8.7<br>(5.4, 15.7)	6.6<br>(4.0, 12.5)	-0.24<br>(-0.46, 0.09)	10.9<br>(6.8, 19.9)	7.5<br>(4.7, 15.0)	-0.31<br>(-0.51, -0.02)
Nauru	0.9<br>(0.6, 1.8)	0.9<br>(0.5, 1.8)	-0.03<br>(-0.30, 0.35)	10.7<br>(6.9, 21.0)	10.3<br>(6.3, 22.7)	-0.04<br>(-0.32, 0.48)
Niue	0.2<br>(0.1, 0.4)	0.2<br>(0.1, 0.3)	-0.10<br>(-0.37, 0.24)	8.3<br>(5.4, 16.7)	11.1<br>(7.0, 23.3)	0.34<br>(-0.07, 0.87)
Northern Mariana Islands	1.4<br>(0.8, 2.6)	1.7<br>(1.0, 3.4)	0.23<br>(-0.13, 0.74)	3.9<br>(2.4, 7.7)	3.4<br>(2.1, 7.0)	-0.14<br>(-0.37, 0.16)
Palau	0.8<br>(0.5, 1.6)	0.8<br>(0.4, 1.6)	-0.07<br>(-0.34, 0.30)	6.3<br>(3.6, 12.7)	4.4<br>(2.2, 9.2)	-0.31<br>(-0.50, -0.06)
Papua New Guinea	533.2<br>(313.0, 851.9)	1243.2<br>(832.1, 2019.8)	1.33<br>(0.66, 2.34)	12.3<br>(7.6, 19.9)	11.3<br>(7.4, 19.5)	-0.08<br>(-0.31, 0.25)

Samoa	12.0<br>(8.1, 20.4)	12.4<br>(8.3, 23.3)	0.03<br>(-0.19, 0.32)	8.6<br>(5.8, 15.5)	6.6<br>(4.4, 12.9)	-0.24<br>(-0.41, -0.03)
Solomon Islands	20.9<br>(12.8, 31.8)	33.6<br>(22.5, 58.8)	0.61<br>(0.09, 1.27)	7.6<br>(4.6, 12.9)	6.3<br>(4.2, 11.9)	-0.17<br>(-0.41, 0.21)
Tokelau	0.1<br>(0.1, 0.3)	0.2<br>(0.1, 0.4)	0.23<br>(-0.19, 0.96)	9.3<br>(5.8, 17.7)	13.3<br>(8.1, 31.6)	0.43<br>(-0.09, 1.28)
Tonga	5.1<br>(3.7, 9.3)	4.4<br>(2.9, 9.0)	-0.15<br>(-0.43, 0.24)	6.1<br>(4.4, 11.6)	4.5<br>(3.0, 9.3)	-0.27<br>(-0.50, 0.02)
Tuvalu	1.2<br>(0.7, 2.3)	0.8<br>(0.5, 1.6)	-0.30<br>(-0.51, 0.07)	12.5<br>(7.3, 23.7)	7.3<br>(4.8, 14.4)	-0.42<br>(-0.58, -0.13)
Vanuatu	12.7<br>(7.7, 21.4)	21.5<br>(14.2, 41.2)	0.70<br>(0.18, 1.36)	9.4<br>(5.9, 16.8)	7.8<br>(5.1, 15.2)	-0.17<br>(-0.40, 0.15)
South Asia	136086.5<br>(77808.1, 184381.0)	136562.7<br>(97809.0, 189353.3)	0.00<br>(-0.23, 0.46)	12.2<br>(7.8, 17.1)	8.5<br>(6.0, 12.5)	-0.30<br>(-0.44, -0.10)
Bangladesh	13289.0<br>(7185.4, 19200.5)	12634.2<br>(7166.9, 19513.8)	-0.05<br>(-0.49, 0.57)	13.3<br>(6.4, 21.9)	8.7<br>(4.8, 13.7)	-0.35<br>(-0.54, -0.02)
Bhutan	91.9<br>(45.0, 137.4)	61.1<br>(33.9, 96.8)	-0.33<br>(-0.64, 0.41)	14.3<br>(7.8, 22.4)	9.3<br>(5.1, 14.2)	-0.35<br>(-0.59, 0.07)
India	99575.4<br>(57534.0, 134279.2)	94736.7<br>(69016.9, 131902.1)	-0.05<br>(-0.29, 0.41)	11.7<br>(7.5, 16.3)	7.7<br>(5.5, 11.4)	-0.34<br>(-0.48, -0.10)
Nepal	3424.0<br>(1746.1, 5124.9)	2556.8<br>(1465.0, 3967.2)	-0.25<br>(-0.58, 0.34)	14.7<br>(8.4, 23.0)	9.4<br>(5.2, 15.0)	-0.36<br>(-0.59, -0.06)
Pakistan	19706.1<br>(10808.8, 28216.3)	26574.0<br>(16921.3, 38115.3)	0.35<br>(-0.03, 0.91)	14.4<br>(8.9, 20.6)	12.0<br>(7.9, 18.3)	-0.17<br>(-0.38, 0.12)
Southeast Asia	27785.8<br>(18241.5, 51942.8)	31111.8<br>(22912.5, 58708.4)	0.12<br>(-0.20, 0.57)	6.3<br>(4.3, 12.7)	4.7<br>(3.4, 9.2)	-0.26<br>(-0.43, 0.00)
Cambodia	722.4<br>(350.9, 1695.1)	776.6<br>(499.9, 1512.9)	0.08<br>(-0.42, 0.97)	6.7<br>(3.5, 14.1)	4.9<br>(3.1, 10.1)	-0.26<br>(-0.54, 0.22)
Indonesia	10340.6<br>(5841.2, 24421.7)	11946.7<br>(7868.4, 24908.0)	0.16<br>(-0.20, 0.73)	6.0<br>(3.4, 13.4)	4.6<br>(3.0, 10.4)	-0.23<br>(-0.43, 0.10)
Lao People's Democratic Republic	478.7<br>(153.7, 1319.5)	479.4<br>(256.0, 980.5)	0.00<br>(-0.39, 0.97)	10.0<br>(3.8, 24.6)	6.7<br>(3.7, 14.0)	-0.33<br>(-0.56, 0.14)

Malaysia	510.7<br>(367.3, 1189.2)	757.2<br>(518.6, 1870.7)	0.48<br>(-0.01, 1.04)	3.2<br>(2.3, 8.4)	2.5<br>(1.7, 6.6)	-0.23<br>(-0.46, 0.04)
Maldives	55.1<br>(33.2, 78.9)	33.7<br>(26.6, 49.8)	-0.39<br>(-0.61, 0.51)	22.1<br>(14.9, 29.9)	7.5<br>(6.0, 10.3)	-0.66<br>(-0.78, -0.42)
Mauritius	109.8<br>(101.7, 119.3)	398.2<br>(351.1, 433.7)	2.63<br>(2.14, 3.09)	10.9<br>(10.1, 11.8)	29.0<br>(25.6, 31.6)	1.65<br>(1.29, 1.99)
Myanmar	4138.5<br>(1916.3, 10101.6)	3636.3<br>(2377.3, 7103.6)	-0.12<br>(-0.44, 0.56)	10.1<br>(5.2, 22.8)	6.8<br>(4.4, 13.8)	-0.33<br>(-0.54, 0.09)
Philippines	3227.3<br>(2172.9, 5818.8)	3530.7<br>(2583.7, 7427.4)	0.09<br>(-0.14, 0.44)	4.9<br>(3.3, 10.3)	3.3<br>(2.4, 7.5)	-0.34<br>(-0.47, -0.14)
Seychelles	2.9<br>(1.9, 6.1)	3.1<br>(1.9, 6.7)	0.07<br>(-0.23, 0.46)	4.3<br>(2.9, 9.4)	2.9<br>(1.8, 6.4)	-0.33<br>(-0.52, -0.09)
Sri Lanka	2711.4<br>(1852.6, 4475.5)	3523.4<br>(2180.3, 5135.3)	0.30<br>(-0.34, 1.23)	17.2<br>(12.2, 28.8)	15.1<br>(9.5, 21.9)	-0.12<br>(-0.54, 0.49)
Thailand	2104.6<br>(1431.4, 4590.3)	2148.6<br>(1424.9, 6094.4)	0.02<br>(-0.31, 0.50)	4.1<br>(2.7, 10.6)	3.1<br>(2.1, 7.5)	-0.25<br>(-0.47, 0.07)
Timor-Leste	75.8<br>(33.1, 193.5)	87.8<br>(51.9, 174.0)	0.16<br>(-0.31, 0.98)	8.0<br>(4.1, 17.1)	6.3<br>(3.8, 13.1)	-0.22<br>(-0.45, 0.15)
Viet Nam	3267.8<br>(2110.5, 6753.6)	3746.8<br>(2123.1, 8856.4)	0.15<br>(-0.27, 0.88)	5.4<br>(3.3, 12.3)	3.8<br>(2.1, 9.6)	-0.29<br>(-0.54, 0.10)
Southern Latin America	7983.4<br>(7181.5, 8803.3)	4739.3<br>(4439.0, 5091.5)	-0.41<br>(-0.48, -0.32)	16.3<br>(14.7, 17.9)	6.5<br>(6.1, 7.1)	-0.60<br>(-0.65, -0.54)
Uruguay	305.4<br>(273.4, 337.1)	218.2<br>(200.0, 238.4)	-0.29<br>(-0.37, -0.17)	9.7<br>(8.7, 10.7)	5.4<br>(5.0, 6.0)	-0.44<br>(-0.51, -0.34)
Argentina	6519.0<br>(5775.2, 7311.8)	3409.0<br>(3182.6, 3695.2)	-0.48<br>(-0.55, -0.39)	19.9<br>(17.6, 22.3)	7.1<br>(6.6, 7.8)	-0.64<br>(-0.69, -0.58)
Chile	1158.7<br>(1084.3, 1237.9)	1111.9<br>(1027.6, 1201.1)	-0.04<br>(-0.14, 0.06)	9.0<br>(8.5, 9.6)	5.4<br>(5.0, 5.9)	-0.40<br>(-0.46, -0.33)
Southern Sub-Saharan Africa	2187.8<br>(1666.9, 2874.6)	3211.9<br>(2359.2, 3891.4)	0.47<br>(0.19, 0.84)	4.7<br>(3.6, 6.4)	4.3<br>(3.2, 5.2)	-0.08<br>(-0.28, 0.14)
Botswana	68.7<br>(46.3, 112.8)	85.7<br>(59.6, 114.4)	0.25<br>(-0.17, 0.83)	6.6<br>(4.3, 10.6)	4.0<br>(2.9, 5.3)	-0.39<br>(-0.60, -0.08)

Eswatini	43.9<br>(30.7, 67.8)	52.9<br>(35.4, 73.3)	0.20<br>(-0.25, 0.88)	6.8<br>(4.7, 10.5)	5.3<br>(3.6, 7.3)	-0.22<br>(-0.52, 0.22)
Lesotho	62.3<br>(38.8, 105.2)	87.0<br>(58.8, 115.7)	0.40<br>(-0.16, 1.18)	4.9<br>(3.1, 8.2)	5.4<br>(3.7, 7.2)	0.10<br>(-0.37, 0.76)
Namibia	66.2<br>(46.8, 97.8)	99.2<br>(67.2, 140.6)	0.50<br>(0.00, 1.23)	5.9<br>(4.2, 8.5)	4.9<br>(3.4, 6.8)	-0.17<br>(-0.44, 0.21)
South Africa	1545<br>(1219.9, 1880.6)	1931.6<br>(1491.0, 2297.2)	0.25<br>(0.02, 0.55)	4.5<br>(3.7, 5.7)	3.6<br>(2.8, 4.2)	-0.21<br>(-0.35, -0.03)
Zimbabwe	401.5<br>(206.8, 677.9)	955.4<br>(595.2, 1431.6)	1.38<br>(0.50, 2.83)	5.2<br>(2.6, 8.9)	7.2<br>(4.7, 10.4)	0.37<br>(-0.12, 1.23)
Tropical Latin America	19064.9<br>(17856.0, 20357.1)	24234.9<br>(23002.0, 25403.3)	0.27<br>(0.17, 0.38)	14.2<br>(13.4, 15.0)	10.2<br>(9.7, 10.8)	-0.28<br>(-0.34, -0.22)
Brazil	18942.6<br>(17688.9, 20254.0)	24078.2<br>(22849.8, 25249.2)	0.27<br>(0.17, 0.38)	14.5<br>(13.6, 15.4)	10.4<br>(9.9, 11.0)	-0.28<br>(-0.34, -0.22)
Paraguay	122.4<br>(87.0, 253.7)	156.7<br>(102.9, 384.2)	0.28<br>(-0.11, 0.82)	3.2<br>(2.2, 7.3)	2.4<br>(1.6, 6.1)	-0.24<br>(-0.46, 0.05)
Western Europe	34599.0<br>(31743.0, 38280.9)	34042.5<br>(31024.4, 36440.0)	-0.02<br>(-0.13, 0.09)	8.3<br>(7.7, 9.2)	5.1<br>(4.8, 5.3)	-0.39<br>(-0.46, -0.34)
Andorra	4.4<br>(3.1, 6.0)	4.1<br>(2.8, 5.9)	-0.06<br>(-0.41, 0.49)	9.2<br>(6.5, 12.6)	3.6<br>(2.5, 4.9)	-0.62<br>(-0.76, -0.38)
Austria	508.6<br>(474.8, 544.9)	494.9<br>(449.8, 541.7)	-0.03<br>(-0.12, 0.06)	5.5<br>(5.1, 5.8)	3.5<br>(3.2, 3.8)	-0.36<br>(-0.42, -0.31)
Belgium	1111.8<br>(990.6, 1388.5)	900.3<br>(801.4, 980.9)	-0.19<br>(-0.37, -0.08)	9.5<br>(8.4, 11.9)	5.2<br>(4.7, 5.6)	-0.45<br>(-0.58, -0.38)
Cyprus	376.9<br>(205.1, 487.6)	304.6<br>(165.5, 379.3)	-0.19<br>(-0.38, 0.18)	52.3<br>(28.9, 66.4)	17.4<br>(9.6, 21.8)	-0.67<br>(-0.74, -0.51)
Denmark	474.0<br>(438.5, 512.5)	425.9<br>(384.4, 462.4)	-0.10<br>(-0.19, 0.00)	8.6<br>(8.0, 9.4)	4.8<br>(4.4, 5.2)	-0.44<br>(-0.50, -0.38)
Finland	173.4<br>(151.6, 195.0)	178.0<br>(159.6, 198.5)	0.03<br>(-0.11, 0.19)	3.1<br>(2.7, 3.5)	2.1<br>(2.0, 2.3)	-0.32<br>(-0.40, -0.21)
France	5948.8<br>(4979.2, 7307.5)	6113.5<br>(5521.0, 6689.4)	0.03<br>(-0.19, 0.25)	9.0<br>(7.7, 11.2)	6.1<br>(5.6, 6.5)	-0.33<br>(-0.47, -0.21)

Germany	8412.2	9441.0	0.12	9.5	6.4	-0.32
	(7108.2, 9615.9)	(8306.1, 10352.5)	(-0.05, 0.36)	(8.0, 10.8)	(5.9, 6.9)	(-0.41, -0.18)
Greece	859.9	1495.1	0.74	7.9	8.3	0.06
	(809.1, 911.7)	(1339.8, 1644.8)	(0.57, 0.93)	(7.4, 8.3)	(7.7, 9.1)	(-0.03, 0.17)
Iceland	14.4	17.0	0.18	5.6	3.8	-0.32
	(13.4, 15.5)	(14.9, 19.1)	(0.03, 0.34)	(5.2, 6.0)	(3.4, 4.3)	(-0.40, -0.23)
Ireland	168.1	189.7	0.13	4.6	3.0	-0.34
	(158.4, 180.0)	(171.6, 208.9)	(0.01, 0.26)	(4.3, 4.9)	(2.7, 3.3)	(-0.41, -0.27)
Israel	797.4	795.6	0.00	16.2	7.2	-0.56
	(705.8, 1033.7)	(718.7, 869.3)	(-0.26, 0.17)	(14.4, 21.0)	(6.5, 7.8)	(-0.67, -0.48)
Italy	4731.4	2779.5	-0.41	7.2	2.8	-0.61
	(4493.0, 4967.9)	(2503.9, 3032.7)	(-0.46, -0.37)	(6.8, 7.5)	(2.6, 3.0)	(-0.64, -0.59)
Luxembourg	35.5	45.5	0.28	8.3	5.1	-0.39
	(33.1, 37.6)	(40.5, 50.8)	(0.14, 0.44)	(7.7, 8.8)	(4.5, 5.6)	(-0.46, -0.32)
Malta	11.9	18.1	0.53	3.2	2.9	-0.09
	(10.9, 12.9)	(16.2, 20.2)	(0.36, 0.71)	(2.9, 3.5)	(2.5, 3.3)	(-0.20, 0.02)
Monaco	2.5	2.8	0.11	5.5	4.5	-0.19
	(1.9, 3.4)	(1.9, 3.8)	(-0.22, 0.58)	(4.2, 7.6)	(3.1, 6.1)	(-0.46, 0.19)
Netherlands	873.0	1004.2	0.15	5.4	3.9	-0.27
	(819.4, 937.6)	(915.1, 1106.0)	(0.05, 0.26)	(5.1, 5.7)	(3.6, 4.3)	(-0.33, -0.21)
Norway	286.8	132.8	-0.54	7.2	1.9	-0.73
	(273.6, 300.5)	(121.4, 148.3)	(-0.57, -0.50)	(6.9, 7.5)	(1.8, 2.1)	(-0.75, -0.71)
Portugal	885.6	1021.6	0.15	9.0	5.8	-0.35
	(833.5, 941.4)	(916.6, 1117.2)	(0.03, 0.27)	(8.5, 9.6)	(5.4, 6.3)	(-0.40, -0.30)
San Marino	0.9	0.9	0.04	3.2	1.6	-0.50
	(0.7, 1.6)	(0.6, 1.9)	(-0.28, 0.49)	(2.5, 5.9)	(1.1, 3.1)	(-0.66, -0.29)
Spain	3742.6	4291.6	0.15	9.2	6.0	-0.35
	(3525.6, 3948.7)	(3844.8, 4655.5)	(0.04, 0.26)	(8.7, 9.7)	(5.5, 6.4)	(-0.40, -0.29)
Sweden	577.9	603.8	0.04	6.1	4.1	-0.32
	(541.5, 616.9)	(539.8, 678.2)	(-0.05, 0.15)	(5.7, 6.5)	(3.7, 4.6)	(-0.38, -0.25)
Switzerland	926.7	715.0	-0.23	12.1	5.1	-0.58
	(811.0, 1136.4)	(633.6, 793.8)	(-0.39, -0.09)	(10.3, 14.9)	(4.6, 5.6)	(-0.67, -0.50)

United Kingdom	3645.9<br>(3233.6, 4483.2)	3037.0<br>(2871.9, 3181.1)	-0.17<br>(-0.33, -0.06)	6.4<br>(5.6, 7.9)	3.7<br>(3.5, 3.9)	-0.42<br>(-0.53, -0.33)
Western Sub-Saharan Africa	19834.3<br>(13552.6, 38853.0)	32071.0<br>(22084.1, 47221.9)	0.62<br>(0.15, 1.22)	9.3<br>(5.2, 16.9)	6.5<br>(3.8, 9.5)	-0.30<br>(-0.48, -0.08)
Benin	441.9<br>(271.3, 851.1)	779.9<br>(486.8, 1192.3)	0.76<br>(0.14, 1.97)	8.0<br>(4.2, 14.0)	5.7<br>(3.0, 9.0)	-0.28<br>(-0.49, 0.06)
Burkina Faso	1141.7<br>(636.9, 1962.5)	1991.6<br>(1161.8, 3180.6)	0.74<br>(0.18, 1.66)	11.1<br>(4.8, 21.4)	9.0<br>(4.2, 14.9)	-0.19<br>(-0.41, 0.13)
Cabo Verde	33.5<br>(16.0, 64.2)	22.4<br>(8.8, 41.0)	-0.33<br>(-0.56, 0.13)	8.6<br>(3.5, 18.3)	4.4<br>(1.8, 8.3)	-0.49<br>(-0.64, -0.17)
Cameroon	846.0<br>(539.0, 1528.2)	1814.2<br>(1238.0, 2862.2)	1.14<br>(0.49, 2.19)	8.4<br>(4.2, 15.3)	6.3<br>(3.8, 10.6)	-0.25<br>(-0.47, 0.12)
Chad	664.4<br>(309.8, 1316)	1766.4<br>(980.1, 3155.6)	1.66<br>(0.86, 3.14)	9.5<br>(4.1, 20.4)	9.2<br>(4.5, 16.4)	-0.03<br>(-0.31, 0.40)
Côte d'Ivoire	1113.8<br>(728.8, 1989.2)	1818.3<br>(1113.9, 2742.3)	0.63<br>(0.11, 1.63)	9.0<br>(4.8, 15.0)	6.8<br>(3.6, 11.0)	-0.24<br>(-0.46, 0.12)
Gambia	79.1<br>(46.2, 145.3)	134.1<br>(70.0, 241.3)	0.70<br>(0.14, 1.43)	8.4<br>(3.7, 15.3)	6.5<br>(3.1, 11.7)	-0.22<br>(-0.44, 0.10)
Ghana	1066.0<br>(720.4, 2056.3)	1256.2<br>(806.2, 2061.6)	0.18<br>(-0.16, 0.78)	8.0<br>(4.5, 14.8)	4.5<br>(2.6, 7.4)	-0.44<br>(-0.62, -0.16)
Guinea	773.1<br>(420.9, 1603.5)	1089.6<br>(641.5, 1802.9)	0.41<br>(-0.14, 1.64)	10.3<br>(5.4, 21.1)	8.0<br>(4.0, 13.8)	-0.22<br>(-0.49, 0.27)
Guinea-Bissau	126.1<br>(74.1, 251.8)	152.9<br>(85.0, 260.8)	0.21<br>(-0.24, 0.99)	12.2<br>(6.7, 22.9)	8.5<br>(4.2, 15.0)	-0.30<br>(-0.50, -0.03)
Liberia	353.5<br>(202.4, 711.0)	339.2<br>(172.7, 574.7)	-0.04<br>(-0.53, 0.95)	11.5<br>(6.8, 20.2)	6.9<br>(3.1, 12.3)	-0.39<br>(-0.65, 0.01)
Mali	879.1<br>(465.3, 1796.8)	1667.1<br>(917.8, 2937.6)	0.90<br>(0.25, 1.88)	9.0<br>(4.5, 19.2)	7.0<br>(3.1, 13.9)	-0.23<br>(-0.46, 0.08)
Mauritania	135.7<br>(85.6, 267.9)	176.8<br>(101.3, 300.1)	0.30<br>(-0.14, 1.04)	6.9<br>(3.8, 14.0)	4.7<br>(2.4, 8.5)	-0.32<br>(-0.50, 0.00)
Niger	954.6<br>(461.4, 1825.1)	1835.1<br>(768.5, 3526.1)	0.92<br>(-0.01, 2.31)	10.4<br>(4.5, 21.0)	7.7<br>(2.5, 15.7)	-0.26<br>(-0.57, 0.10)

Nigeria	9447.1<br>(6711.9, 19378.9)	15080.7<br>(10536.5, 21578.0)	0.60<br>(0.03, 1.35)	9.4<br>(5.3, 16.7)	6.0<br>(4.1, 8.8)	-0.35<br>(-0.56, -0.06)
Sao Tome and Principe	8.7<br>(5.3, 17.6)	7.7<br>(3.3, 15.5)	-0.11<br>(-0.56, 0.61)	6.6<br>(3.7, 12.3)	4.4<br>(1.8, 8.4)	-0.34<br>(-0.58, 0.05)
Senegal	820.4<br>(535.4, 1575.0)	904.4<br>(433.4, 1527.5)	0.10<br>(-0.38, 0.88)	9.8<br>(5.1, 17.6)	6.4<br>(2.7, 11.2)	-0.35<br>(-0.56, -0.03)
Sierra Leone	644.3<br>(352.7, 1346.1)	774.4<br>(456.7, 1267.8)	0.20<br>(-0.29, 1.22)	11.6<br>(6.9, 19.9)	8.2<br>(4.5, 13.6)	-0.29<br>(-0.53, 0.13)
Togo	304.7<br>(194.9, 569.8)	459.6<br>(236.9, 734.4)	0.51<br>(-0.09, 1.57)	8.4<br>(4.4, 15.5)	6.4<br>(3.0, 10.4)	-0.24<br>(-0.47, 0.09)

DALYs: Disability adjusted life years.

Table S4. Death cases and age-standardized rate for pulmonary arterial hypertension in 1990 and 2021, and estimated percentage change from 1990 to 2021, by 204 countries and 21 regions

Location	Number			Age-standardized Rate		
	1990	2021	Percent change	1990	2021	Percent change
Global	14842.5 (12369.9, 17484.9)	22020.5 (18239.2, 25351.6)	0.48 (0.21, 0.78)	0.4 (0.3, 0.4)	0.3 (0.2, 0.3)	-0.22 (-0.35, -0.08)
Andean Latin America	83.5 (56.0, 111.9)	91.3 (71.9, 119.4)	0.09 (-0.19, 0.60)	0.3 (0.2, 0.3)	0.2 (0.1, 0.2)	-0.45 (-0.57, -0.26)
Bolivia (Plurinational State of)	23.1 (12.2, 37.1)	21.2 (14.8, 30.4)	-0.09 (-0.39, 0.50)	0.4 (0.3, 0.6)	0.2 (0.2, 0.3)	-0.43 (-0.58, -0.16)
Ecuador	18.1 (14.2, 22.4)	27.3 (22.9, 32.3)	0.51 (0.14, 1.08)	0.3 (0.2, 0.3)	0.2 (0.2, 0.2)	-0.34 (-0.52, -0.03)
Peru	42.4 (29.2, 57.2)	42.8 (30.8, 62.5)	0.01 (-0.31, 0.53)	0.2 (0.2, 0.3)	0.1 (0.1, 0.2)	-0.49 (-0.63, -0.28)
Australasia	45.5 (39.1, 57.2)	57.7 (49.1, 64.9)	0.27 (-0.02, 0.50)	0.2 (0.2, 0.3)	0.1 (0.1, 0.1)	-0.45 (-0.58, -0.36)
Australia	40.3 (34.0, 51.8)	50.1 (42.1, 56.9)	0.24 (-0.07, 0.50)	0.2 (0.2, 0.3)	0.1 (0.1, 0.1)	-0.48 (-0.61, -0.38)
New Zealand	5.2 (4.8, 5.6)	7.6 (6.7, 8.5)	0.46 (0.28, 0.64)	0.1 (0.1, 0.2)	0.1 (0.1, 0.1)	-0.28 (-0.36, -0.18)
Caribbean	124.2 (83.4, 168.9)	95.4 (65.6, 129.7)	-0.23 (-0.40, 0.01)	0.4 (0.3, 0.5)	0.2 (0.1, 0.3)	-0.47 (-0.59, -0.32)
Antigua and Barbuda	0.1 (0.1, 0.1)	0.0 (0.0, 0.0)	-0.38 (-0.47, -0.29)	0.1 (0.1, 0.1)	0.0 (0.0, 0.0)	-0.64 (-0.69, -0.58)
Bahamas	2.1 (1.9, 2.4)	1.6 (1.3, 2.0)	-0.26 (-0.42, -0.05)	1.1 (0.9, 1.2)	0.4 (0.3, 0.5)	-0.62 (-0.70, -0.52)
Barbados	3.0 (2.5, 3.4)	1.6 (1.3, 2.0)	-0.45 (-0.57, -0.29)	1.1 (0.9, 1.2)	0.4 (0.3, 0.4)	-0.66 (-0.73, -0.56)
Belize	0.8 (0.7, 0.9)	0.5 (0.4, 0.6)	-0.36 (-0.46, -0.26)	0.5 (0.4, 0.5)	0.1 (0.1, 0.2)	-0.68 (-0.74, -0.63)

Bermuda	1.0<br>(0.9, 1.2)	0.7<br>(0.6, 0.9)	-0.28<br>(-0.43, -0.10)	1.7<br>(1.5, 2.0)	0.6<br>(0.5, 0.7)	-0.67<br>(-0.74, -0.58)
Cuba	10.8<br>(9.9, 11.8)	8.6<br>(7.5, 9.9)	-0.20<br>(-0.33, -0.06)	0.1<br>(0.1, 0.1)	0.0<br>(0.0, 0.1)	-0.53<br>(-0.61, -0.44)
Dominica	0.1<br>(0.1, 0.2)	0.1<br>(0.0, 0.2)	-0.38<br>(-0.60, 0.03)	0.2<br>(0.1, 0.3)	0.1<br>(0.1, 0.2)	-0.47<br>(-0.65, -0.12)
Dominican Republic	11.6<br>(8.5, 16.3)	8.8<br>(6.0, 23.3)	-0.25<br>(-0.54, 0.58)	0.2<br>(0.1, 0.3)	0.1<br>(0.1, 0.2)	-0.50<br>(-0.66, -0.19)
Grenada	0.6<br>(0.6, 0.8)	0.3<br>(0.2, 0.3)	-0.58<br>(-0.66, -0.48)	0.8<br>(0.7, 1.0)	0.3<br>(0.2, 0.3)	-0.68<br>(-0.75, -0.60)
Guyana	1.2<br>(1.0, 1.3)	2.7<br>(2.0, 3.5)	1.28<br>(0.70, 1.96)	0.2<br>(0.2, 0.2)	0.4<br>(0.3, 0.5)	1.32<br>(0.74, 2.02)
Haiti	55.1<br>(21.1, 95.2)	53.3<br>(26.6, 86.9)	-0.03<br>(-0.34, 0.52)	0.9<br>(0.4, 1.3)	0.5<br>(0.3, 0.7)	-0.44<br>(-0.60, -0.20)
Jamaica	4.4<br>(3.8, 4.9)	2.1<br>(1.7, 2.7)	-0.52<br>(-0.65, -0.37)	0.2<br>(0.2, 0.2)	0.1<br>(0.1, 0.1)	-0.66<br>(-0.75, -0.55)
Puerto Rico	20.8<br>(19.2, 22.6)	6.1<br>(5.0, 7.2)	-0.71<br>(-0.76, -0.65)	0.6<br>(0.5, 0.6)	0.1<br>(0.1, 0.1)	-0.83<br>(-0.86, -0.79)
Saint Kitts and Nevis	0.1<br>(0.1, 0.1)	0.0<br>(0.0, 0.0)	-0.47<br>(-0.64, -0.32)	0.2<br>(0.2, 0.3)	0.1<br>(0.1, 0.1)	-0.65<br>(-0.76, -0.56)
Saint Lucia	0.8<br>(0.7, 0.9)	0.5<br>(0.4, 0.6)	-0.33<br>(-0.46, -0.18)	0.8<br>(0.7, 0.9)	0.2<br>(0.2, 0.3)	-0.70<br>(-0.76, -0.63)
Saint Vincent and the Grenadines	0.1<br>(0.1, 0.1)	0.1<br>(0.1, 0.1)	-0.04<br>(-0.19, 0.16)	0.1<br>(0.1, 0.1)	0.1<br>(0.1, 0.1)	-0.30<br>(-0.40, -0.17)
Suriname	2.5<br>(1.5, 3.0)	1.9<br>(1.4, 2.8)	-0.24<br>(-0.47, 0.53)	0.7<br>(0.5, 0.9)	0.3<br>(0.2, 0.5)	-0.56<br>(-0.69, -0.21)
Trinidad and Tobago	4.6<br>(4.2, 5.0)	3.0<br>(2.3, 3.9)	-0.34<br>(-0.49, -0.14)	0.5<br>(0.4, 0.5)	0.2<br>(0.1, 0.2)	-0.61<br>(-0.71, -0.49)
United States Virgin Islands	0.4<br>(0.2, 0.5)	0.2<br>(0.1, 0.4)	-0.46<br>(-0.65, -0.04)	0.4<br>(0.2, 0.5)	0.2<br>(0.1, 0.3)	-0.62<br>(-0.74, -0.42)
Central Asia	208.2<br>(162.6, 239.8)	318.5<br>(260.9, 381.6)	0.53<br>(0.28, 0.87)	0.4<br>(0.3, 0.5)	0.4<br>(0.3, 0.5)	0.04<br>(-0.13, 0.26)

Armenia	6.8<br>(5.7, 8.2)	3.2<br>(2.6, 3.8)	-0.53<br>(-0.65, -0.39)	0.3<br>(0.2, 0.3)	0.1<br>(0.1, 0.1)	-0.69<br>(-0.77, -0.59)
Azerbaijan	37.0<br>(24.7, 52.0)	53.7<br>(32.5, 82.5)	0.45<br>(-0.09, 1.30)	0.7<br>(0.5, 1.0)	0.6<br>(0.4, 0.8)	-0.18<br>(-0.45, 0.22)
Georgia	21.1<br>(16.3, 26.8)	58<br>(45.5, 72.6)	1.75<br>(0.95, 2.83)	0.4<br>(0.3, 0.5)	1.0<br>(0.8, 1.3)	1.72<br>(0.94, 2.81)
Kazakhstan	6.0<br>(4.6, 7.4)	7.5<br>(6.1, 9.0)	0.26<br>(-0.09, 0.81)	0.0<br>(0.0, 0.1)	0.0<br>(0.0, 0.1)	0.06<br>(-0.25, 0.57)
Kyrgyzstan	3.1<br>(2.6, 3.7)	2.4<br>(2.0, 3.0)	-0.22<br>(-0.41, 0.03)	0.1<br>(0.1, 0.1)	0.1<br>(0.0, 0.1)	-0.47<br>(-0.59, -0.32)
Mongolia	20.1<br>(11.2, 32.2)	34.2<br>(19.9, 44.0)	0.7<br>(0.11, 1.66)	1.7<br>(1.0, 2.9)	1.6<br>(0.9, 2.0)	-0.09<br>(-0.41, 0.47)
Tajikistan	41.5<br>(25.2, 54.3)	53.2<br>(34.3, 72.5)	0.28<br>(-0.13, 0.86)	1.2<br>(0.7, 1.7)	0.8<br>(0.5, 1.1)	-0.34<br>(-0.56, -0.06)
Turkmenistan	4.2<br>(3.2, 5.3)	9.5<br>(7.1, 12.4)	1.27<br>(0.63, 2.23)	0.2<br>(0.1, 0.2)	0.2<br>(0.2, 0.3)	0.27<br>(-0.09, 0.87)
Uzbekistan	68.4<br>(54.4, 84.6)	96.8<br>(77.1, 119.0)	0.42<br>(0.03, 0.94)	0.5<br>(0.3, 0.6)	0.4<br>(0.3, 0.5)	-0.19<br>(-0.44, 0.16)
Central Europe	355.0<br>(309.1, 393.9)	437.6<br>(397.8, 478.9)	0.23<br>(0.07, 0.44)	0.3<br>(0.2, 0.3)	0.2<br>(0.2, 0.2)	-0.18<br>(-0.28, -0.04)
Albania	11.4<br>(7.6, 14.8)	11.0<br>(7.1, 19.3)	-0.04<br>(-0.38, 0.74)	0.5<br>(0.4, 0.7)	0.3<br>(0.2, 0.5)	-0.46<br>(-0.65, -0.08)
Bosnia and Herzegovina	10.5<br>(7.2, 14.2)	12.3<br>(8.9, 16.0)	0.17<br>(-0.22, 0.73)	0.3<br>(0.2, 0.4)	0.2<br>(0.2, 0.3)	-0.22<br>(-0.49, 0.15)
Bulgaria	15.6<br>(13.2, 18.3)	11.4<br>(8.5, 15.0)	-0.27<br>(-0.48, 0.03)	0.2<br>(0.1, 0.2)	0.1<br>(0.1, 0.1)	-0.41<br>(-0.59, -0.18)
Croatia	6.4<br>(5.5, 7.5)	4.0<br>(3.3, 4.8)	-0.37<br>(-0.48, -0.24)	0.1<br>(0.1, 0.1)	0.0<br>(0.0, 0.1)	-0.61<br>(-0.67, -0.53)
Czechia	50.8<br>(42.6, 59.9)	88.5<br>(75.7, 101.1)	0.74<br>(0.41, 1.08)	0.4<br>(0.3, 0.5)	0.4<br>(0.4, 0.5)	0.09<br>(-0.12, 0.32)
Hungary	43.3<br>(37.9, 49.4)	30.8<br>(25.1, 38.3)	-0.29<br>(-0.45, -0.11)	0.3<br>(0.3, 0.4)	0.2<br>(0.1, 0.2)	-0.49<br>(-0.61, -0.36)

Montenegro	0.3<br>(0.1, 1.4)	0.3<br>(0.1, 1.7)	0.11<br>(-0.33, 0.64)	0.0<br>(0.0, 0.2)	0.0<br>(0.0, 0.2)	-0.22<br>(-0.54, 0.15)
North Macedonia	2.6<br>(1.7, 4.2)	3.4<br>(1.8, 6.2)	0.29<br>(-0.23, 0.87)	0.2<br>(0.1, 0.3)	0.1<br>(0.1, 0.2)	-0.19<br>(-0.49, 0.17)
Poland	69.0<br>(59.6, 79.2)	77.2<br>(69.4, 84.1)	0.12<br>(-0.06, 0.34)	0.2<br>(0.1, 0.2)	0.1<br>(0.1, 0.1)	-0.27<br>(-0.39, -0.13)
Romania	119.7<br>(96.2, 144.1)	170.1<br>(145.8, 194.9)	0.42<br>(0.12, 0.88)	0.5<br>(0.4, 0.6)	0.5<br>(0.4, 0.6)	0.02<br>(-0.19, 0.33)
Serbia	12.8<br>(8.0, 32.0)	14.8<br>(9.0, 30.2)	0.16<br>(-0.20, 0.77)	0.1<br>(0.1, 0.4)	0.1<br>(0.1, 0.2)	-0.36<br>(-0.57, 0.00)
Slovakia	6.0<br>(3.7, 12.3)	5.6<br>(3.5, 11.4)	-0.06<br>(-0.35, 0.41)	0.1<br>(0.1, 0.2)	0.1<br>(0.0, 0.1)	-0.39<br>(-0.57, -0.07)
Slovenia	0.9<br>(0.8, 1.0)	1.8<br>(1.5, 2.1)	0.98<br>(0.61, 1.40)	0.0<br>(0.0, 0.0)	0.0<br>(0.0, 0.0)	-0.01<br>(-0.20, 0.21)
Central Latin America	179.8<br>(157.3, 209.5)	201.3<br>(176.9, 230.3)	0.12<br>(-0.09, 0.33)	0.2<br>(0.1, 0.2)	0.1<br>(0.1, 0.1)	-0.48<br>(-0.57, -0.37)
Colombia	34.2<br>(28.2, 42.9)	51.9<br>(43.0, 62.5)	0.52<br>(0.10, 1.03)	0.2<br>(0.1, 0.2)	0.1<br>(0.1, 0.1)	-0.38<br>(-0.54, -0.17)
Costa Rica	8.4<br>(7.6, 9.2)	5.6<br>(4.8, 6.3)	-0.33<br>(-0.43, -0.23)	0.4<br>(0.4, 0.5)	0.1<br>(0.1, 0.1)	-0.76<br>(-0.79, -0.72)
El Salvador	12.2<br>(8.6, 15.6)	7.7<br>(5.3, 15.9)	-0.37<br>(-0.61, 0.25)	0.3<br>(0.2, 0.4)	0.1<br>(0.1, 0.2)	-0.59<br>(-0.75, -0.29)
Guatemala	13.7<br>(12.2, 16.4)	6.7<br>(5.7, 7.8)	-0.51<br>(-0.63, -0.40)	0.3<br>(0.2, 0.3)	0.1<br>(0.0, 0.1)	-0.78<br>(-0.82, -0.72)
Honduras	6.7<br>(4.2, 10.0)	11.7<br>(7.7, 18.5)	0.75<br>(0.19, 1.45)	0.3<br>(0.1, 0.4)	0.2<br>(0.1, 0.3)	-0.24<br>(-0.49, 0.13)
Mexico	83.5<br>(75.2, 99.6)	100.7<br>(88.6, 112.3)	0.21<br>(-0.04, 0.43)	0.1<br>(0.1, 0.2)	0.1<br>(0.1, 0.1)	-0.42<br>(-0.53, -0.29)
Nicaragua	4.1<br>(2.2, 6.5)	2.3<br>(1.4, 8.8)	-0.44<br>(-0.70, 0.52)	0.1<br>(0.1, 0.3)	0.0<br>(0.0, 0.2)	-0.67<br>(-0.80, -0.29)
Panama	3.2<br>(2.7, 3.5)	2.6<br>(2.0, 3.1)	-0.19<br>(-0.38, 0.05)	0.2<br>(0.2, 0.2)	0.1<br>(0.0, 0.1)	-0.70<br>(-0.77, -0.61)

Venezuela (Bolivarian Republic of)	13.9<br>(12.1, 15.7)	12.0<br>(9.2, 15.3)	-0.14<br>(-0.36, 0.16)	0.1<br>(0.1, 0.1)	0.0<br>(0.0, 0.1)	-0.60<br>(-0.70, -0.46)
Central Sub-Saharan Africa	86.7<br>(54.9, 162.6)	131.2<br>(61.8, 237.4)	0.51<br>(-0.10, 1.23)	0.2<br>(0.1, 0.5)	0.2<br>(0.1, 0.4)	-0.21<br>(-0.40, 0.05)
Angola	17.8<br>(10.6, 39.1)	30.5<br>(16.4, 57.1)	0.71<br>(0.02, 1.91)	0.3<br>(0.1, 0.6)	0.2<br>(0.1, 0.4)	-0.30<br>(-0.48, 0.06)
Central African Republic	5.4<br>(3.1, 10.7)	7.7<br>(3.8, 13.8)	0.43<br>(-0.05, 1.08)	0.3<br>(0.1, 0.7)	0.2<br>(0.1, 0.4)	-0.18<br>(-0.38, 0.10)
Congo	3.2<br>(2.1, 6.6)	4.4<br>(2.6, 8.3)	0.41<br>(0.02, 1.02)	0.2<br>(0.1, 0.5)	0.1<br>(0.1, 0.3)	-0.33<br>(-0.49, -0.10)
Democratic Republic of the Congo	58.2<br>(35.5, 104.7)	86.3<br>(36.2, 160.6)	0.48<br>(-0.21, 1.28)	0.2<br>(0.1, 0.5)	0.2<br>(0.1, 0.4)	-0.17<br>(-0.40, 0.13)
Equatorial Guinea	0.7<br>(0.4, 1.4)	0.8<br>(0.4, 1.4)	0.11<br>(-0.33, 1.11)	0.3<br>(0.1, 0.6)	0.1<br>(0.1, 0.2)	-0.57<br>(-0.75, -0.18)
Gabon	1.4<br>(0.8, 2.6)	1.4<br>(0.8, 2.6)	0.05<br>(-0.28, 0.59)	0.2<br>(0.1, 0.4)	0.1<br>(0.1, 0.2)	-0.38<br>(-0.58, -0.03)
East Asia	4114.7<br>(3141.4, 5526.1)	7490.0<br>(4986.5, 9266.1)	0.82<br>(0.18, 1.64)	0.6<br>(0.4, 0.8)	0.4<br>(0.3, 0.5)	-0.31<br>(-0.55, 0.01)
China mainland	4058.9<br>(3098.6, 5452.2)	7318.0<br>(4835.7, 9075.8)	0.80<br>(0.17, 1.63)	0.6<br>(0.5, 0.8)	0.4<br>(0.3, 0.5)	-0.31<br>(-0.56, 0.01)
Democratic People's Republic of Korea	43.3<br>(29.4, 78.3)	95.2<br>(66.9, 160.1)	1.2<br>(0.54, 2.21)	0.3<br>(0.2, 0.7)	0.3<br>(0.2, 0.6)	0.02<br>(-0.29, 0.47)
Taiwan (Province of China mainland)	12.6<br>(11.9, 13.3)	76.8<br>(65.7, 84.9)	5.10<br>(4.30, 5.79)	0.1<br>(0.1, 0.1)	0.2<br>(0.2, 0.2)	0.93<br>(0.71, 1.14)
Eastern Europe	562.9<br>(511.9, 651.3)	278.3<br>(257.9, 300.3)	-0.51<br>(-0.58, -0.44)	0.2<br>(0.2, 0.3)	0.1<br>(0.1, 0.1)	-0.62<br>(-0.67, -0.56)
Belarus	4.7<br>(3.6, 5.8)	5.8<br>(4.7, 6.9)	0.23<br>(-0.10, 0.71)	0.0<br>(0.0, 0.1)	0.0<br>(0.0, 0.0)	-0.05<br>(-0.29, 0.29)
Estonia	0.4<br>(0.3, 0.4)	1.1<br>(0.9, 1.3)	1.90<br>(1.35, 2.54)	0.0<br>(0.0, 0.1)	0.0<br>(0.0, 0.1)	0.71<br>(0.40, 1.06)
Latvia	0.7<br>(0.6, 0.8)	4.2<br>(3.5, 4.8)	4.86<br>(3.72, 6.08)	0.0<br>(0.0, 0.1)	0.1<br>(0.1, 0.1)	3.52<br>(2.65, 4.42)

Lithuania	1.4<br>(1.2, 1.6)	4.5<br>(3.8, 5.2)	2.17<br>(1.58, 2.90)	0.0<br>(0.0, 0.1)	0.1<br>(0.1, 0.1)	1.2<br>(0.82, 1.68)
Republic of Moldova	0.1<br>(0.1, 0.1)	0.3<br>(0.3, 0.4)	3.25<br>(2.54, 4.25)	0.0<br>(0.0, 0.1)	0.0<br>(0.0, 0.1)	2.54<br>(1.93, 3.39)
Russian Federation	514.6<br>(465.1, 600.0)	220.7<br>(204.1, 238.3)	-0.57<br>(-0.64, -0.51)	0.3<br>(0.3, 0.4)	0.1<br>(0.1, 0.1)	-0.69<br>(-0.74, -0.65)
Ukraine	41.0<br>(37.0, 45.7)	41.7<br>(30.9, 53.1)	0.02<br>(-0.27, 0.34)	0.1<br>(0.1, 0.1)	0.1<br>(0.1, 0.1)	0.04<br>(-0.25, 0.35)
Eastern Sub-Saharan Africa	365.5<br>(216.9, 686.7)	467.8<br>(219.4, 878.0)	0.28<br>(-0.12, 0.74)	0.3<br>(0.1, 0.5)	0.2<br>(0.1, 0.3)	-0.35<br>(-0.46, -0.14)
Burundi	15.5<br>(8.8, 31.2)	15.9<br>(6.4, 31.9)	0.02<br>(-0.45, 0.67)	0.4<br>(0.2, 0.8)	0.2<br>(0.1, 0.4)	-0.43<br>(-0.70, -0.13)
Comoros	1.0<br>(0.6, 1.7)	0.8<br>(0.4, 1.7)	-0.12<br>(-0.44, 0.38)	0.3<br>(0.1, 0.5)	0.2<br>(0.1, 0.3)	-0.44<br>(-0.61, -0.16)
Djibouti	0.6<br>(0.3, 1.2)	1.2<br>(0.5, 2.3)	1.01<br>(0.14, 2.57)	0.2<br>(0.1, 0.5)	0.2<br>(0.1, 0.3)	-0.33<br>(-0.53, 0.03)
Eritrea	7.1<br>(3.9, 14.4)	8.5<br>(4.0, 16.2)	0.20<br>(-0.22, 0.84)	0.3<br>(0.1, 0.7)	0.2<br>(0.1, 0.4)	-0.29<br>(-0.49, 0.06)
Ethiopia	91.7<br>(48.3, 174.3)	97.4<br>(38.2, 203.4)	0.06<br>(-0.36, 0.66)	0.3<br>(0.1, 0.5)	0.1<br>(0, 0.3)	-0.45<br>(-0.61, -0.22)
Kenya	19.3<br>(10.2, 40.1)	32.9<br>(14.3, 65.3)	0.70<br>(0.27, 1.45)	0.1<br>(0.1, 0.3)	0.1<br>(0.0, 0.2)	-0.16<br>(-0.36, 0.34)
Madagascar	37.9<br>(23.0, 75.0)	58.2<br>(31.0, 112.1)	0.54<br>(0.09, 1.07)	0.4<br>(0.2, 0.8)	0.3<br>(0.1, 0.6)	-0.26<br>(-0.46, -0.03)
Malawi	17.1<br>(10.4, 31.7)	18.7<br>(9.1, 33.1)	0.10<br>(-0.34, 0.73)	0.2<br>(0.1, 0.4)	0.2<br>(0.1, 0.3)	-0.25<br>(-0.42, 0.02)
Mozambique	25.8<br>(14.2, 47.2)	40.2<br>(18.0, 70.3)	0.56<br>(0.02, 1.43)	0.3<br>(0.1, 0.6)	0.2<br>(0.1, 0.5)	-0.12<br>(-0.37, 0.29)
Rwanda	18.0<br>(10.5, 38.6)	13.9<br>(6.20, 29.6)	-0.23<br>(-0.59, 0.37)	0.3<br>(0.2, 0.7)	0.2<br>(0.1, 0.4)	-0.51<br>(-0.71, -0.25)
Somalia	19.7<br>(8.5, 36.7)	34.2<br>(9.4, 73.4)	0.74<br>(-0.09, 1.61)	0.4<br>(0.1, 0.8)	0.3<br>(0.1, 0.6)	-0.26<br>(-0.54, 0.08)

South Sudan	15.1<br>(7.7, 29.3)	16.9<br>(8.8, 30.3)	0.12<br>(-0.25, 0.86)	0.3<br>(0.1, 0.7)	0.2<br>(0.1, 0.4)	-0.27<br>(-0.47, 0.11)
Uganda	37.4<br>(20.2, 67.6)	40.7<br>(22.2, 72.1)	0.09<br>(-0.26, 0.69)	0.3<br>(0.1, 0.6)	0.1<br>(0.1, 0.3)	-0.46<br>(-0.68, -0.07)
United Republic of Tanzania	47.0<br>(27.5, 88.9)	62.0<br>(31.4, 113.1)	0.32<br>(-0.08, 0.87)	0.3<br>(0.1, 0.5)	0.2<br>(0.1, 0.3)	-0.38<br>(-0.57, -0.07)
Zambia	12.2<br>(7.3, 25.5)	25.7<br>(9.5, 51.7)	1.11<br>(-0.13, 2.98)	0.2<br>(0.1, 0.5)	0.2<br>(0.1, 0.5)	0.03<br>(-0.45, 0.62)
High-income Asia Pacific	433.7<br>(409.9, 459.3)	1048.8<br>(825.8, 1200.5)	1.42<br>(1.00, 1.74)	0.3<br>(0.2, 0.3)	0.2<br>(0.2, 0.3)	-0.10<br>(-0.19, -0.02)
Brunei Darussalam	0.9<br>(0.5, 1.2)	1.0<br>(0.6, 1.2)	0.08<br>(-0.21, 0.67)	0.5<br>(0.3, 0.6)	0.3<br>(0.2, 0.3)	-0.43<br>(-0.58, -0.18)
Japan	384.9<br>(365.5, 401.0)	1002.4<br>(785.8, 1147.3)	1.60<br>(1.16, 1.93)	0.3<br>(0.3, 0.3)	0.3<br>(0.3, 0.3)	-0.02<br>(-0.10, 0.06)
Republic of Korea	42.3<br>(28.8, 71.6)	39.4<br>(22.0, 136.2)	-0.07<br>(-0.51, 0.93)	0.1<br>(0.1, 0.3)	0.1<br>(0.0, 0.2)	-0.57<br>(-0.75, -0.27)
Singapore	5.7<br>(5.3, 6.1)	6.0<br>(5.3, 6.6)	0.05<br>(-0.08, 0.18)	0.2<br>(0.2, 0.2)	0.1<br>(0.1, 0.1)	-0.62<br>(-0.66, -0.57)
High-income North America	1064.4<br>(946.6, 1167.0)	1879.8<br>(1619.7, 2043)	0.77<br>(0.58, 0.96)	0.3<br>(0.3, 0.3)	0.3<br>(0.3, 0.3)	-0.08<br>(-0.16, 0.01)
Canada	92.9<br>(85.4, 99.6)	94.6<br>(82.7, 103.8)	0.02<br>(-0.09, 0.13)	0.3<br>(0.3, 0.3)	0.1<br>(0.1, 0.2)	-0.54<br>(-0.58, -0.50)
Greenland	0.3<br>(0.1, 0.4)	0.1<br>(0.1, 0.2)	-0.64<br>(-0.77, -0.04)	0.8<br>(0.4, 1.0)	0.2<br>(0.1, 0.3)	-0.75<br>(-0.84, -0.38)
United States of America	971.2<br>(855.5, 1070.2)	1785.1<br>(1535.4, 1944.9)	0.84<br>(0.64, 1.05)	0.3<br>(0.3, 0.3)	0.3<br>(0.3, 0.3)	-0.02<br>(-0.12, 0.08)
North Africa and Middle East	2141.8<br>(1308.8, 2739.3)	1895.8<br>(1328.3, 2305.0)	-0.11<br>(-0.44, 0.30)	0.8<br>(0.6, 1.0)	0.4<br>(0.3, 0.5)	-0.42<br>(-0.57, -0.20)
Afghanistan	38.0<br>(19.5, 60.7)	125.4<br>(56.3, 188.6)	2.30<br>(0.42, 4.21)	0.4<br>(0.2, 0.7)	0.7<br>(0.2, 1.1)	0.64<br>(-0.35, 1.65)
Algeria	52.1<br>(33.6, 94.2)	119.3<br>(42.5, 184.6)	1.29<br>(-0.33, 3.02)	0.3<br>(0.2, 0.7)	0.4<br>(0.1, 0.6)	0.27<br>(-0.53, 1.19)

Bahrain	0.5<br>(0.3, 1.0)	1.1<br>(0.5, 2.0)	1.38<br>(0.13, 2.58)	0.2<br>(0.1, 0.5)	0.2<br>(0.1, 0.3)	-0.18<br>(-0.56, 0.24)
Egypt	727.2<br>(364.7, 1033.2)	282.2<br>(220.5, 376.8)	-0.61<br>(-0.76, -0.18)	1.2<br>(0.8, 1.6)	0.4<br>(0.3, 0.5)	-0.69<br>(-0.78, -0.49)
Iran (Islamic Republic of)	470.6<br>(306.8, 592.6)	428.6<br>(285.6, 494.0)	-0.09<br>(-0.40, 0.34)	1.4<br>(0.9, 1.9)	0.6<br>(0.4, 0.7)	-0.56<br>(-0.69, -0.36)
Iraq	37.4<br>(20.8, 57.3)	37.6<br>(19.8, 79.9)	0.00<br>(-0.34, 0.65)	0.2<br>(0.1, 0.5)	0.1<br>(0.1, 0.4)	-0.40<br>(-0.62, -0.06)
Jordan	1.6<br>(0.8, 6.7)	4.3<br>(1.9, 14.2)	1.64<br>(-0.05, 4.11)	0.1<br>(0.0, 0.3)	0.1<br>(0.0, 0.2)	-0.02<br>(-0.62, 1.09)
Kuwait	0.9<br>(0.8, 1.0)	4.0<br>(3.4, 4.7)	3.30<br>(2.50, 4.23)	0.1<br>(0.1, 0.1)	0.1<br>(0.1, 0.2)	0.91<br>(0.59, 1.28)
Lebanon	20.6<br>(12.8, 31.2)	23.4<br>(17.5, 38.1)	0.14<br>(-0.31, 1.29)	0.9<br>(0.5, 1.3)	0.4<br>(0.3, 0.6)	-0.58<br>(-0.74, -0.26)
Libya	14.5<br>(8.5, 27.4)	17.8<br>(8.1, 31.2)	0.23<br>(-0.52, 1.08)	0.3<br>(0.2, 0.6)	0.4<br>(0.2, 0.6)	0.17<br>(-0.54, 1.07)
Morocco	71.8<br>(40.4, 126.4)	131.0<br>(50.3, 201.2)	0.83<br>(-0.44, 2.11)	0.3<br>(0.2, 0.7)	0.4<br>(0.2, 0.7)	0.32<br>(-0.52, 1.23)
Oman	1.3<br>(0.7, 3.8)	2.6<br>(1.0, 5.1)	0.99<br>(-0.42, 3.57)	0.1<br>(0.1, 0.4)	0.1<br>(0.0, 0.2)	0.07<br>(-0.65, 1.61)
Palestine	4.2<br>(1.9, 7.8)	3.8<br>(1.5, 10.5)	-0.08<br>(-0.41, 0.45)	0.2<br>(0.1, 0.6)	0.1<br>(0.1, 0.4)	-0.43<br>(-0.61, -0.13)
Qatar	0.3<br>(0.2, 0.6)	1.3<br>(0.7, 2.1)	3.56<br>(0.66, 6.86)	0.2<br>(0.1, 0.4)	0.1<br>(0.1, 0.2)	-0.22<br>(-0.69, 0.33)
Saudi Arabia	14.5<br>(8.9, 30.6)	18.0<br>(9.2, 35.6)	0.25<br>(-0.36, 1.22)	0.1<br>(0.1, 0.4)	0.1<br>(0.0, 0.2)	-0.42<br>(-0.71, 0.02)
Sudan	71.8<br>(36.5, 131.8)	124.0<br>(59.6, 183.2)	0.73<br>(-0.39, 2.23)	0.4<br>(0.2, 0.7)	0.4<br>(0.2, 0.6)	0.13<br>(-0.59, 0.97)
Syrian Arab Republic	10.2<br>(5.4, 38.1)	6.2<br>(2.7, 33.0)	-0.39<br>(-0.71, 0.01)	0.1<br>(0.1, 0.4)	0.1<br>(0.0, 0.3)	-0.41<br>(-0.65, -0.04)
Tunisia	15.2<br>(9.9, 28.4)	39.6<br>(12.2, 66.7)	1.60<br>(-0.28, 4.31)	0.3<br>(0.2, 0.5)	0.4<br>(0.1, 0.6)	0.41<br>(-0.54, 1.88)

Türkiye	537.6<br>(331.3, 723.6)	407.9<br>(312.9, 561.6)	-0.24<br>(-0.47, 0.39)	1.2<br>(0.8, 1.6)	0.5<br>(0.4, 0.7)	-0.58<br>(-0.72, -0.32)
United Arab Emirates	6.5<br>(3.4, 9.6)	9.7<br>(5.4, 12.5)	0.49<br>(-0.02, 1.39)	0.7<br>(0.4, 1.1)	0.4<br>(0.2, 0.5)	-0.48<br>(-0.66, -0.16)
Yemen	43.8<br>(21.4, 72.6)	106.2<br>(49.2, 160.2)	1.42<br>(-0.11, 2.92)	0.4<br>(0.2, 0.8)	0.6<br>(0.2, 0.9)	0.38<br>(-0.45, 1.43)
Oceania	11.9<br>(7.6, 19.2)	24.9<br>(16.7, 43.3)	1.10<br>(0.63, 1.75)	0.3<br>(0.2, 0.5)	0.2<br>(0.2, 0.5)	-0.14<br>(-0.31, 0.06)
American Samoa	0.0<br>(0.0, 0.1)	0.1<br>(0.0, 0.1)	0.29<br>(-0.12, 0.79)	0.2<br>(0.1, 0.4)	0.1<br>(0.1, 0.3)	-0.26<br>(-0.46, 0.04)
Cook Islands	0.0<br>(0.0, 0.1)	0.0<br>(0.0, 0.1)	-0.18<br>(-0.48, 0.27)	0.3<br>(0.2, 0.5)	0.1<br>(0.1, 0.3)	-0.48<br>(-0.67, -0.18)
Fiji	0.9<br>(0.5, 1.7)	1.0<br>(0.5, 2.1)	0.16<br>(-0.20, 0.57)	0.2<br>(0.1, 0.4)	0.1<br>(0.1, 0.3)	-0.28<br>(-0.50, -0.03)
Guam	0.1<br>(0.1, 0.2)	0.1<br>(0.1, 0.2)	-0.07<br>(-0.39, 0.31)	0.1<br>(0.1, 0.4)	0.1<br>(0.0, 0.1)	-0.62<br>(-0.74, -0.46)
Kiribati	0.1<br>(0.1, 0.3)	0.2<br>(0.1, 0.4)	0.40<br>(0.05, 0.91)	0.3<br>(0.2, 0.7)	0.3<br>(0.2, 0.6)	-0.16<br>(-0.39, 0.14)
Marshall Islands	0.1<br>(0.0, 0.1)	0.1<br>(0.0, 0.2)	0.25<br>(-0.18, 0.81)	0.3<br>(0.2, 0.7)	0.2<br>(0.1, 0.5)	-0.30<br>(-0.52, 0.01)
Micronesia (Federated States of)	0.2<br>(0.1, 0.4)	0.2<br>(0.1, 0.3)	-0.16<br>(-0.40, 0.18)	0.4<br>(0.2, 0.8)	0.2<br>(0.2, 0.5)	-0.33<br>(-0.53, -0.07)
Nauru	0.0<br>(0.0, 0.0)	0.0<br>(0.0, 0.0)	0.10<br>(-0.22, 0.68)	0.3<br>(0.2, 0.6)	0.4<br>(0.2, 1.0)	0.16<br>(-0.28, 1.15)
Niue	0.0<br>(0.0, 0.0)	0.0<br>(0.0, 0.0)	-0.34<br>(-0.55, -0.07)	0.3<br>(0.2, 0.6)	0.2<br>(0.1, 0.5)	-0.10<br>(-0.37, 0.25)
Northern Mariana Islands	0<br>(0, 0.1)	0.0<br>(0.0, 0.1)	0.78<br>(0.22, 1.57)	0.1<br>(0.1, 0.3)	0.1<br>(0.1, 0.3)	-0.14<br>(-0.35, 0.13)
Palau	0.0<br>(0.0, 0.0)	0.0<br>(0.0, 0.0)	0.14<br>(-0.19, 0.58)	0.2<br>(0.1, 0.4)	0.1<br>(0.1, 0.3)	-0.31<br>(-0.50, -0.09)
Papua New Guinea	8.6<br>(5.2, 13.8)	20.5<br>(13.2, 35.7)	1.39<br>(0.77, 2.32)	0.3<br>(0.2, 0.6)	0.3<br>(0.2, 0.6)	-0.11<br>(-0.33, 0.21)

Samoa	0.3<br>(0.2, 0.5)	0.3<br>(0.2, 0.6)	0.15<br>(-0.11, 0.47)	0.3<br>(0.2, 0.6)	0.2<br>(0.1, 0.5)	-0.27<br>(-0.45, -0.05)
Solomon Islands	0.4<br>(0.2, 0.7)	0.8<br>(0.5, 1.5)	0.90<br>(0.31, 1.75)	0.3<br>(0.1, 0.5)	0.2<br>(0.1, 0.5)	-0.16<br>(-0.39, 0.23)
Tokelau	0.0<br>(0.0, 0.0)	0.0<br>(0.0, 0.0)	0.02<br>(-0.31, 0.59)	0.3<br>(0.2, 0.6)	0.3<br>(0.2, 0.7)	-0.02<br>(-0.34, 0.52)
Tonga	0.1<br>(0.1, 0.2)	0.1<br>(0.1, 0.3)	0.01<br>(-0.32, 0.44)	0.2<br>(0.1, 0.5)	0.1<br>(0.1, 0.3)	-0.29<br>(-0.52, -0.01)
Tuvalu	0.0<br>(0.0, 0.0)	0.0<br>(0.0, 0.0)	-0.14<br>(-0.38, 0.27)	0.4<br>(0.2, 0.8)	0.2<br>(0.1, 0.5)	-0.39<br>(-0.56, -0.11)
Vanuatu	0.2<br>(0.1, 0.4)	0.4<br>(0.3, 0.9)	0.90<br>(0.33, 1.67)	0.3<br>(0.2, 0.6)	0.2<br>(0.1, 0.5)	-0.19<br>(-0.42, 0.11)
South Asia	2385.2<br>(1502.0, 3417.9)	3548.8<br>(2321.1, 5531.8)	0.49<br>(0.15, 1.05)	0.3<br>(0.2, 0.5)	0.3<br>(0.2, 0.4)	-0.19<br>(-0.38, 0.04)
Bangladesh	262.8<br>(124.4, 437.1)	382.6<br>(189.3, 663.5)	0.46<br>(-0.08, 1.19)	0.4<br>(0.2, 0.9)	0.3<br>(0.1, 0.6)	-0.29<br>(-0.51, 0.11)
Bhutan	1.5<br>(0.8, 2.3)	1.8<br>(0.9, 3.0)	0.24<br>(-0.28, 1.13)	0.4<br>(0.2, 0.7)	0.3<br>(0.2, 0.5)	-0.21<br>(-0.46, 0.29)
India	1755.7<br>(1131.9, 2495.5)	2611.6<br>(1738.6, 4059.8)	0.49<br>(0.11, 1.12)	0.3<br>(0.2, 0.5)	0.2<br>(0.2, 0.4)	-0.20<br>(-0.41, 0.08)
Nepal	54.4<br>(31.0, 84.7)	67.9<br>(34.1, 116.7)	0.25<br>(-0.27, 0.91)	0.4<br>(0.2, 0.7)	0.3<br>(0.1, 0.6)	-0.19<br>(-0.43, 0.18)
Pakistan	310.8<br>(190.4, 457.7)	485.0<br>(311.5, 747.2)	0.56<br>(0.19, 1.15)	0.3<br>(0.2, 0.5)	0.3<br>(0.2, 0.6)	-0.06<br>(-0.32, 0.29)
Southeast Asia	505.8<br>(339.9, 1094.4)	741.5<br>(525.5, 1849.9)	0.47<br>(0.09, 1.02)	0.2<br>(0.1, 0.4)	0.1<br>(0.1, 0.3)	-0.21<br>(-0.39, 0.10)
Cambodia	11.9<br>(6.0, 25.8)	16.8<br>(10.1, 38.1)	0.41<br>(-0.19, 1.49)	0.2<br>(0.1, 0.4)	0.1<br>(0.1, 0.4)	-0.21<br>(-0.46, 0.26)
Indonesia	185.0<br>(106.1, 426.3)	267.7<br>(170.3, 672.4)	0.45<br>(0.04, 1.09)	0.1<br>(0.1, 0.4)	0.1<br>(0.1, 0.4)	-0.15<br>(-0.38, 0.21)
Lao People's Democratic Republic	7.6<br>(2.8, 19.0)	8.8<br>(4.8, 20.0)	0.16<br>(-0.27, 1.01)	0.2<br>(0.1, 0.5)	0.2<br>(0.1, 0.4)	-0.33<br>(-0.54, 0.02)

Malaysia	10.0<br>(6.7, 30.3)	19.1<br>(11.5, 64.9)	0.91<br>(0.22, 1.72)	0.1<br>(0.1, 0.3)	0.1<br>(0.0, 0.3)	-0.16<br>(-0.47, 0.19)
Maldives	0.8<br>(0.6, 1.2)	0.7<br>(0.6, 1.1)	-0.12<br>(-0.43, 0.79)	0.5<br>(0.4, 0.7)	0.2<br>(0.1, 0.3)	-0.63<br>(-0.74, -0.44)
Mauritius	2.3<br>(2.1, 2.5)	11.0<br>(9.8, 12.0)	3.84<br>(3.22, 4.48)	0.3<br>(0.2, 0.3)	0.7<br>(0.6, 0.8)	1.71<br>(1.39, 2.06)
Myanmar	70.0<br>(37.2, 150.7)	75.4<br>(48.3, 178.2)	0.08<br>(-0.32, 0.85)	0.2<br>(0.1, 0.6)	0.2<br>(0.1, 0.4)	-0.30<br>(-0.51, 0.10)
Philippines	51.5<br>(33.5, 115.2)	69.9<br>(47.9, 183.5)	0.36<br>(0.04, 0.88)	0.1<br>(0.1, 0.3)	0.1<br>(0.0, 0.2)	-0.30<br>(-0.46, -0.07)
Seychelles	0.1<br>(0.0, 0.2)	0.1<br>(0.0, 0.2)	0.13<br>(-0.20, 0.51)	0.1<br>(0.1, 0.4)	0.1<br>(0.0, 0.3)	-0.34<br>(-0.54, -0.11)
Sri Lanka	55.0<br>(39.5, 91.9)	101.3<br>(63.1, 147.6)	0.84<br>(-0.08, 2.14)	0.4<br>(0.3, 0.7)	0.4<br>(0.3, 0.6)	0.00<br>(-0.50, 0.68)
Thailand	39.8<br>(25.4, 123.1)	62.4<br>(34.5, 281.8)	0.57<br>(-0.04, 1.54)	0.1<br>(0.1, 0.4)	0.1<br>(0.0, 0.3)	-0.24<br>(-0.49, 0.14)
Timor-Leste	1.1<br>(0.5, 2.5)	1.6<br>(1.0, 3.7)	0.47<br>(-0.12, 1.44)	0.2<br>(0.1, 0.5)	0.2<br>(0.1, 0.4)	-0.18<br>(-0.42, 0.21)
Viet Nam	69.8<br>(38.4, 193.9)	105.6<br>(53.0, 331.8)	0.51<br>(-0.08, 1.48)	0.1<br>(0.1, 0.5)	0.1<br>(0.1, 0.4)	-0.22<br>(-0.51, 0.25)
Southern Latin America	169.3<br>(150.6, 186.2)	150.0<br>(138.5, 161.8)	-0.11<br>(-0.23, 0.02)	0.4<br>(0.3, 0.4)	0.2<br>(0.2, 0.2)	-0.49<br>(-0.56, -0.41)
Uruguay	7.8<br>(6.8, 8.6)	7.9<br>(7.1, 8.7)	0.02<br>(-0.12, 0.19)	0.2<br>(0.2, 0.2)	0.2<br>(0.1, 0.2)	-0.31<br>(-0.40, -0.19)
Argentina	137.3<br>(120.1, 152.8)	108.5<br>(100.0, 117.7)	-0.21<br>(-0.32, -0.08)	0.4<br>(0.4, 0.5)	0.2<br>(0.2, 0.2)	-0.53<br>(-0.59, -0.44)
Chile	24.3<br>(22.8, 25.8)	33.6<br>(30.4, 36.6)	0.38<br>(0.24, 0.52)	0.2<br>(0.2, 0.2)	0.1<br>(0.1, 0.2)	-0.33<br>(-0.40, -0.26)
Southern Sub-Saharan Africa	42.6<br>(32.3, 58.4)	71.7<br>(53.0, 85.7)	0.68<br>(0.31, 1.10)	0.1<br>(0.1, 0.2)	0.1<br>(0.1, 0.1)	-0.07<br>(-0.29, 0.21)
Botswana	1.4<br>(0.9, 2.2)	1.8<br>(1.2, 2.4)	0.31<br>(-0.14, 0.98)	0.2<br>(0.1, 0.3)	0.1<br>(0.1, 0.1)	-0.42<br>(-0.61, -0.10)

Eswatini	0.8<br>(0.6, 1.3)	1.0<br>(0.7, 1.5)	0.28<br>(-0.21, 1.00)	0.2<br>(0.1, 0.3)	0.1<br>(0.1, 0.2)	-0.28<br>(-0.55, 0.18)
Lesotho	1.4<br>(0.9, 2.4)	1.9<br>(1.3, 2.6)	0.33<br>(-0.23, 1.19)	0.1<br>(0.1, 0.2)	0.1<br>(0.1, 0.2)	0.00<br>(-0.43, 0.74)
Namibia	1.4<br>(1.0, 2.0)	2.2<br>(1.5, 3.0)	0.60<br>(0.06, 1.38)	0.2<br>(0.1, 0.2)	0.1<br>(0.1, 0.2)	-0.18<br>(-0.44, 0.19)
South Africa	29.4<br>(23.8, 37.5)	46.4<br>(34.9, 54.5)	0.58<br>(0.26, 0.94)	0.1<br>(0.1, 0.1)	0.1<br>(0.1, 0.1)	-0.11<br>(-0.33, 0.11)
Zimbabwe	8.3<br>(4.1, 14.1)	18.4<br>(11.9, 26.9)	1.23<br>(0.40, 2.65)	0.2<br>(0.1, 0.3)	0.2<br>(0.1, 0.3)	0.20<br>(-0.21, 1.01)
Tropical Latin America	394.1<br>(372.8, 412.5)	779.4<br>(713.7, 822.5)	0.98<br>(0.82, 1.14)	0.4<br>(0.4, 0.4)	0.3<br>(0.3, 0.3)	-0.14<br>(-0.20, -0.08)
Brazil	391.7<br>(370.9, 410.4)	775.1<br>(709.7, 818.5)	0.98<br>(0.82, 1.14)	0.4<br>(0.4, 0.4)	0.3<br>(0.3, 0.3)	-0.14<br>(-0.20, -0.09)
Paraguay	2.4<br>(1.6, 6.5)	4.2<br>(2.6, 12.4)	0.76<br>(0.16, 1.53)	0.1<br>(0.1, 0.3)	0.1<br>(0.0, 0.2)	-0.15<br>(-0.45, 0.23)
Western Europe	1233.0<br>(1094.0, 1380.1)	1787.5<br>(1533.4, 1942.7)	0.45<br>(0.25, 0.65)	0.2<br>(0.2, 0.3)	0.2<br>(0.2, 0.2)	-0.24<br>(-0.34, -0.15)
Andorra	0.1<br>(0.1, 0.2)	0.2<br>(0.1, 0.3)	0.39<br>(-0.12, 1.19)	0.2<br>(0.2, 0.3)	0.1<br>(0.1, 0.2)	-0.54<br>(-0.70, -0.28)
Austria	20.0<br>(18.3, 21.5)	24.9<br>(21.8, 27.6)	0.25<br>(0.12, 0.38)	0.2<br>(0.2, 0.2)	0.1<br>(0.1, 0.1)	-0.27<br>(-0.34, -0.20)
Belgium	44.1<br>(37.9, 55.6)	45.0<br>(37.7, 50.3)	0.02<br>(-0.20, 0.19)	0.3<br>(0.3, 0.4)	0.2<br>(0.2, 0.2)	-0.41<br>(-0.54, -0.33)
Cyprus	13.0<br>(6.7, 17.3)	14.5<br>(7.3, 18.6)	0.12<br>(-0.18, 0.66)	2.1<br>(1.1, 2.7)	0.8<br>(0.4, 1.0)	-0.62<br>(-0.71, -0.42)
Denmark	16.6<br>(14.9, 18.2)	21.0<br>(18.0, 23.1)	0.26<br>(0.11, 0.44)	0.2<br>(0.2, 0.3)	0.2<br>(0.2, 0.2)	-0.24<br>(-0.32, -0.15)
Finland	5.7<br>(4.9, 6.6)	7.7<br>(6.5, 8.7)	0.35<br>(0.13, 0.62)	0.1<br>(0.1, 0.1)	0.1<br>(0.1, 0.1)	-0.30<br>(-0.40, -0.16)
France	239.0<br>(190.9, 295.8)	325.0<br>(279.5, 361.1)	0.36<br>(0.06, 0.74)	0.3<br>(0.2, 0.4)	0.2<br>(0.2, 0.2)	-0.31<br>(-0.45, -0.13)

Germany	304.2<br>(253.6, 350.4)	543.7<br>(452.6, 604.0)	0.79<br>(0.46, 1.22)	0.3<br>(0.2, 0.3)	0.3<br>(0.2, 0.3)	-0.05<br>(-0.20, 0.17)
Greece	31.9<br>(29.3, 34.2)	87.2<br>(75.9, 97.7)	1.73<br>(1.42, 2.10)	0.2<br>(0.2, 0.3)	0.3<br>(0.3, 0.4)	0.33<br>(0.20, 0.48)
Iceland	0.4<br>(0.4, 0.5)	0.8<br>(0.6, 0.9)	0.71<br>(0.46, 0.99)	0.2<br>(0.1, 0.2)	0.1<br>(0.1, 0.1)	-0.17<br>(-0.28, -0.05)
Ireland	5.9<br>(5.4, 6.3)	8.4<br>(7.2, 9.5)	0.44<br>(0.26, 0.63)	0.2<br>(0.1, 0.2)	0.1<br>(0.1, 0.1)	-0.28<br>(-0.35, -0.18)
Israel	22.3<br>(19.1, 29.0)	34.9<br>(29.9, 39.3)	0.57<br>(0.18, 0.89)	0.5<br>(0.4, 0.6)	0.3<br>(0.2, 0.3)	-0.43<br>(-0.57, -0.32)
Italy	170.6<br>(159.1, 179.0)	137.9<br>(115.4, 152.5)	-0.19<br>(-0.29, -0.13)	0.2<br>(0.2, 0.2)	0.1<br>(0.1, 0.1)	-0.58<br>(-0.61, -0.55)
Luxembourg	1.3<br>(1.2, 1.4)	2.3<br>(2.0, 2.6)	0.72<br>(0.50, 0.99)	0.3<br>(0.2, 0.3)	0.2<br>(0.2, 0.2)	-0.23<br>(-0.33, -0.13)
Malta	0.4<br>(0.3, 0.4)	0.8<br>(0.7, 0.9)	1.17<br>(0.88, 1.49)	0.1<br>(0.1, 0.1)	0.1<br>(0.1, 0.1)	-0.07<br>(-0.18, 0.05)
Monaco	0.1<br>(0.1, 0.2)	0.1<br>(0.1, 0.2)	0.23<br>(-0.15, 0.80)	0.2<br>(0.1, 0.2)	0.1<br>(0.1, 0.2)	-0.16<br>(-0.43, 0.23)
Netherlands	30.2<br>(27.8, 32.7)	47.8<br>(41.3, 53.3)	0.58<br>(0.41, 0.78)	0.2<br>(0.1, 0.2)	0.1<br>(0.1, 0.2)	-0.15<br>(-0.23, -0.05)
Norway	7.3<br>(6.8, 7.6)	4.8<br>(4.2, 5.2)	-0.34<br>(-0.40, -0.30)	0.1<br>(0.1, 0.1)	0.1<br>(0.0, 0.1)	-0.64<br>(-0.66, -0.61)
Portugal	28.1<br>(26.3, 30.1)	56.1<br>(47.7, 63.0)	0.99<br>(0.72, 1.23)	0.2<br>(0.2, 0.3)	0.2<br>(0.2, 0.2)	-0.12<br>(-0.21, -0.03)
San Marino	0.0<br>(0.0, 0.1)	0.0<br>(0.0, 0.1)	0.26<br>(-0.19, 0.89)	0.1<br>(0.1, 0.2)	0.1<br>(0.0, 0.1)	-0.51<br>(-0.69, -0.26)
Spain	131.5<br>(121.1, 139.7)	240.6<br>(200.2, 270.1)	0.83<br>(0.59, 1.06)	0.3<br>(0.3, 0.3)	0.2<br>(0.2, 0.2)	-0.19<br>(-0.26, -0.10)
Sweden	17.5<br>(16.0, 18.6)	25.8<br>(22.0, 28.9)	0.48<br>(0.29, 0.66)	0.1<br>(0.1, 0.1)	0.1<br>(0.1, 0.1)	-0.13<br>(-0.23, -0.03)
Switzerland	35.2<br>(29.6, 42.7)	37.4<br>(30.5, 42.5)	0.06<br>(-0.16, 0.28)	0.4<br>(0.3, 0.4)	0.2<br>(0.2, 0.2)	-0.48<br>(-0.59, -0.39)

United Kingdom	106.3<br>(95.9, 132.3)	118.9<br>(106.7, 125.7)	0.12<br>(-0.11, 0.24)	0.1<br>(0.1, 0.2)	0.1<br>(0.1, 0.1)	-0.29<br>(-0.43, -0.20)
Western Sub-Saharan Africa	334.7<br>(194.9, 616.5)	523.3<br>(305.7, 773.9)	0.56<br>(0.16, 1.08)	0.2<br>(0.1, 0.5)	0.2<br>(0.1, 0.3)	-0.33<br>(-0.52, -0.07)
Benin	7.2<br>(4.1, 12.7)	12.8<br>(6.7, 20.5)	0.78<br>(0.21, 1.81)	0.2<br>(0.1, 0.4)	0.2<br>(0.1, 0.3)	-0.28<br>(-0.50, 0.06)
Burkina Faso	20.1<br>(9.2, 38.2)	36.0<br>(17.3, 59.5)	0.79<br>(0.29, 1.52)	0.3<br>(0.1, 0.7)	0.3<br>(0.1, 0.5)	-0.16<br>(-0.41, 0.19)
Cabo Verde	0.6<br>(0.2, 1.4)	0.6<br>(0.2, 1.1)	-0.08<br>(-0.35, 0.50)	0.2<br>(0.1, 0.5)	0.1<br>(0.0, 0.2)	-0.38<br>(-0.58, 0.08)
Cameroon	15.2<br>(8.0, 28.0)	31.6<br>(19.0, 53.5)	1.08<br>(0.51, 2.11)	0.2<br>(0.1, 0.5)	0.2<br>(0.1, 0.3)	-0.30<br>(-0.52, 0.12)
Chad	11.4<br>(5.2, 24.3)	27.4<br>(14.6, 48.7)	1.40<br>(0.68, 2.59)	0.3<br>(0.1, 0.6)	0.2<br>(0.1, 0.5)	-0.05<br>(-0.34, 0.40)
Côte d'Ivoire	17.5<br>(10.3, 29.9)	30.7<br>(16.0, 49.6)	0.76<br>(0.25, 1.65)	0.2<br>(0.1, 0.5)	0.2<br>(0.1, 0.3)	-0.27<br>(-0.49, 0.09)
Gambia	1.3<br>(0.6, 2.3)	2.6<br>(1.2, 4.7)	0.92<br>(0.39, 1.69)	0.2<br>(0.1, 0.5)	0.2<br>(0.1, 0.3)	-0.20<br>(-0.46, 0.24)
Ghana	19.4<br>(11.2, 36.7)	25.9<br>(14.2, 44.4)	0.34<br>(-0.06, 0.99)	0.2<br>(0.1, 0.5)	0.1<br>(0.1, 0.2)	-0.41<br>(-0.63, -0.06)
Guinea	13.0<br>(6.9, 26.6)	18.6<br>(9.2, 32.1)	0.43<br>(-0.08, 1.39)	0.3<br>(0.1, 0.6)	0.2<br>(0.1, 0.4)	-0.18<br>(-0.44, 0.27)
Guinea-Bissau	2.1<br>(1.2, 3.9)	2.7<br>(1.3, 4.7)	0.27<br>(-0.13, 0.85)	0.3<br>(0.1, 0.6)	0.2<br>(0.1, 0.4)	-0.28<br>(-0.44, -0.04)
Liberia	5.4<br>(3.4, 9.9)	5.9<br>(2.6, 10.7)	0.09<br>(-0.42, 0.88)	0.3<br>(0.1, 0.5)	0.2<br>(0.1, 0.4)	-0.31<br>(-0.52, 0.03)
Mali	14.4<br>(7.7, 30.0)	27.4<br>(12.8, 52.8)	0.90<br>(0.33, 1.7)	0.2<br>(0.1, 0.5)	0.2<br>(0.1, 0.4)	-0.22<br>(-0.42, 0.11)
Mauritania	2.7<br>(1.5, 5.5)	3.7<br>(1.8, 6.9)	0.39<br>(-0.04, 1.08)	0.2<br>(0.1, 0.5)	0.1<br>(0.1, 0.3)	-0.30<br>(-0.51, 0.04)
Niger	14.7<br>(7.1, 27.5)	30.6<br>(10.8, 61.2)	1.08<br>(0.12, 2.27)	0.3<br>(0.1, 0.7)	0.2<br>(0.1, 0.5)	-0.19<br>(-0.46, 0.12)

Nigeria	161.2<br>(92.5, 296.3)	227.6<br>(153.3, 332.4)	0.41<br>(-0.05, 1.08)	0.2<br>(0.1, 0.5)	0.1<br>(0.1, 0.2)	-0.43<br>(-0.63, -0.11)
Sao Tome and Principe	0.2<br>(0.1, 0.3)	0.2<br>(0.1, 0.3)	0.10<br>(-0.35, 0.75)	0.2<br>(0.1, 0.4)	0.1<br>(0.0, 0.2)	-0.22<br>(-0.43, 0.11)
Senegal	13.4<br>(7.4, 24.5)	17.9<br>(7.2, 31.8)	0.33<br>(-0.17, 1.05)	0.3<br>(0.1, 0.5)	0.2<br>(0.1, 0.3)	-0.28<br>(-0.52, 0.03)
Sierra Leone	9.9<br>(6.0, 18.0)	12.4<br>(6.6, 20.6)	0.25<br>(-0.21, 1.06)	0.3<br>(0.1, 0.5)	0.2<br>(0.1, 0.3)	-0.27<br>(-0.47, 0.07)
Togo	4.9<br>(2.8, 9.4)	8.8<br>(4.0, 14.6)	0.79<br>(0.16, 1.85)	0.2<br>(0.1, 0.5)	0.2<br>(0.1, 0.3)	-0.20<br>(-0.43, 0.21)

Table S5. Prevalence cases and age-standardized rate for pulmonary arterial hypertension in 1990 and 2021, and estimated percentage change from 1990 to 2021, by 204 countries and 21 regions

Location	Number			Age-standardized Rate		
	1990	2021	Percent change	1990	2021	Percent change
Global	105703.3 (86381.4, 130334.3)	191808.2 (155356.9, 235787.1)	0.81 (0.73, 0.89)	2.3 (1.9, 2.8)	2.3 (1.8, 2.8)	-0.01 (-0.02, 0.00)
Andean Latin America	812.2 (666.9, 996.6)	1777.2 (1434.0, 2193.6)	1.19 (1.05, 1.33)	2.9 (2.3, 3.6)	2.8 (2.2, 3.4)	-0.05 (-0.08, -0.02)
Bolivia (Plurinational State of)	118.6 (96.3, 145.6)	265.9 (215.2, 329.4)	1.24 (1.10, 1.42)	2.6 (2.1, 3.2)	2.5 (2.0, 3.0)	-0.06 (-0.11, 0.00)
Ecuador	213.0 (173.7, 264.1)	518.6 (422.3, 640.2)	1.44 (1.26, 1.60)	2.9 (2.3, 3.6)	3.0 (2.4, 3.6)	0.02 (-0.03, 0.07)
Peru	480.7 (393.9, 592.1)	992.6 (804.1, 1223.5)	1.07 (0.92, 1.22)	3.0 (2.4, 3.7)	2.8 (2.2, 3.4)	-0.08 (-0.12, -0.03)
Australasia	669.8 (543.5, 828)	1166.8 (935.9, 1444.3)	0.74 (0.64, 0.85)	3.0 (2.4, 3.7)	2.8 (2.3, 3.4)	-0.05 (-0.09, -0.01)
Australia	561.5 (458.2, 695.1)	991.9 (794.1, 1225.6)	0.77 (0.66, 0.88)	3.0 (2.5, 3.7)	2.9 (2.3, 3.5)	-0.04 (-0.09, 0.00)
New Zealand	108.3 (87.2, 132.7)	174.9 (141.4, 217.7)	0.62 (0.52, 0.71)	2.9 (2.4, 3.6)	2.6 (2.1, 3.2)	-0.10 (-0.14, -0.06)
Caribbean	778.0 (634.8, 953.7)	1228.9 (987.1, 1506.0)	0.58 (0.49, 0.67)	2.6 (2.1, 3.1)	2.4 (1.9, 2.9)	-0.07 (-0.09, -0.04)
Antigua and Barbuda	1.6 (1.3, 2.0)	2.8 (2.3, 3.5)	0.75 (0.62, 0.90)	2.9 (2.4, 3.6)	2.7 (2.2, 3.4)	-0.06 (-0.11, -0.02)
Bahamas	5.4 (4.4, 6.6)	9.0 (7.3, 11.2)	0.68 (0.53, 0.82)	2.6 (2.1, 3.1)	2.1 (1.7, 2.6)	-0.17 (-0.22, -0.13)
Barbados	7.0 (5.6, 8.4)	10.0 (8.0, 12.3)	0.43 (0.32, 0.55)	2.6 (2.1, 3.2)	2.5 (2.0, 3.0)	-0.06 (-0.10, -0.01)
Belize	2.7 (2.3, 3.4)	8.3 (6.7, 10.2)	2.01 (1.81, 2.23)	2.1 (1.7, 2.6)	2.2 (1.8, 2.6)	0.03 (-0.02, 0.09)

Bermuda	1.9<br>(1.5, 2.3)	2.6<br>(2.1, 3.2)	0.38<br>(0.25, 0.51)	2.9<br>(2.3, 3.6)	2.7<br>(2.2, 3.3)	-0.06<br>(-0.10, -0.01)
Cuba	288.9<br>(234.1, 352.4)	388.6<br>(309.1, 482.3)	0.35<br>(0.22, 0.46)	2.7<br>(2.2, 3.3)	2.5<br>(2.1, 3.1)	-0.05<br>(-0.10, 0.00)
Dominica	1.3<br>(1.1, 1.6)	1.7<br>(1.4, 2.1)	0.29<br>(0.19, 0.40)	2.1<br>(1.7, 2.6)	2.2<br>(1.8, 2.7)	0.05<br>(-0.01, 0.11)
Dominican Republic	150.2<br>(122.5, 185.5)	286.2<br>(230.7, 352.7)	0.91<br>(0.77, 1.05)	2.9<br>(2.3, 3.5)	2.7<br>(2.1, 3.2)	-0.07<br>(-0.12, -0.02)
Grenada	1.7<br>(1.4, 2.1)	2.3<br>(1.9, 2.9)	0.38<br>(0.27, 0.50)	2.3<br>(1.9, 2.8)	2.1<br>(1.7, 2.5)	-0.11<br>(-0.16, -0.06)
Guyana	11.9<br>(9.8, 14.9)	14.3<br>(11.6, 17.6)	0.20<br>(0.11, 0.30)	2.1<br>(1.7, 2.6)	2.0<br>(1.6, 2.4)	-0.05<br>(-0.11, 0.00)
Haiti	88.4<br>(72.4, 109.5)	189.1<br>(151.8, 236.8)	1.14<br>(1.00, 1.30)	2.0<br>(1.6, 2.4)	1.8<br>(1.5, 2.3)	-0.06<br>(-0.11, 0.00)
Jamaica	52.2<br>(42.2, 62.9)	79.3<br>(64.2, 98.2)	0.52<br>(0.42, 0.63)	2.6<br>(2.1, 3.2)	2.6<br>(2.1, 3.2)	0.00<br>(-0.05, 0.05)
Puerto Rico	100.5<br>(79.9, 122.1)	126.2<br>(101.3, 157.6)	0.26<br>(0.15, 0.38)	2.8<br>(2.2, 3.4)	2.6<br>(2.1, 3.2)	-0.05<br>(-0.10, 0.00)
Saint Kitts and Nevis	0.9<br>(0.7, 1.1)	1.4<br>(1.1, 1.7)	0.56<br>(0.40, 0.73)	2.3<br>(1.9, 2.8)	2.0<br>(1.6, 2.4)	-0.16<br>(-0.20, -0.10)
Saint Lucia	2.5<br>(2.0, 3.0)	5.1<br>(4.0, 6.3)	1.05<br>(0.84, 1.27)	2.3<br>(1.8, 2.8)	2.3<br>(1.9, 2.9)	0.02<br>(-0.03, 0.08)
Saint Vincent and the Grenadines	1.8<br>(1.5, 2.2)	2.8<br>(2.3, 3.5)	0.55<br>(0.40, 0.71)	2.1<br>(1.7, 2.6)	2.2<br>(1.7, 2.7)	0.02<br>(-0.03, 0.08)
Suriname	8.5<br>(7.0, 10.4)	15.7<br>(12.7, 19.4)	0.85<br>(0.68, 0.99)	2.7<br>(2.2, 3.3)	2.5<br>(2.1, 3.1)	-0.06<br>(-0.11, -0.01)
Trinidad and Tobago	21.8<br>(17.9, 26.5)	38.8<br>(31.1, 48.2)	0.77<br>(0.62, 0.95)	2.1<br>(1.7, 2.6)	2.3<br>(1.8, 2.8)	0.07<br>(0.02, 0.14)
United States Virgin Islands	2.6<br>(2.1, 3.2)	3.1<br>(2.5, 3.9)	0.21<br>(0.06, 0.38)	2.6<br>(2.1, 3.2)	2.4<br>(2.0, 2.9)	-0.05<br>(-0.10, 0.00)
Central Asia	1408.7<br>(1145.2, 1726.8)	2161.9<br>(1757.9, 2676.5)	0.53<br>(0.46, 0.60)	2.5<br>(2.0, 3.0)	2.3<br>(1.9, 2.9)	-0.05<br>(-0.07, -0.03)

Armenia	90.1<br>(74.3, 110.1)	100.7<br>(80.6, 124.8)	0.12<br>(0.04, 0.21)	2.9<br>(2.4, 3.5)	2.8<br>(2.3, 3.4)	-0.03<br>(-0.08, 0.02)
Azerbaijan	138.1<br>(112.9, 171.8)	298.9<br>(242.3, 370.2)	1.16<br>(1.01, 1.34)	2.2<br>(1.8, 2.7)	2.7<br>(2.2, 3.2)	0.21<br>(0.14, 0.28)
Georgia	157.4<br>(126.8, 192.4)	118.4<br>(96.0, 145.4)	-0.25<br>(-0.29, -0.20)	2.7<br>(2.2, 3.2)	2.6<br>(2.1, 3.1)	-0.03<br>(-0.08, 0.02)
Kazakhstan	368.0<br>(297.7, 444.9)	442.0<br>(360.5, 545.1)	0.20<br>(0.13, 0.27)	2.5<br>(2.0, 3.0)	2.3<br>(1.9, 2.8)	-0.07<br>(-0.12, -0.03)
Kyrgyzstan	87.9<br>(73.0, 106.4)	163.5<br>(133.5, 201.0)	0.86<br>(0.75, 0.98)	2.4<br>(2.0, 3.0)	2.7<br>(2.2, 3.3)	0.13<br>(0.07, 0.19)
Mongolia	28.3<br>(23.0, 35.1)	62.9<br>(50.9, 78.3)	1.22<br>(1.05, 1.44)	1.8<br>(1.5, 2.2)	2.1<br>(1.7, 2.6)	0.16<br>(0.11, 0.22)
Tajikistan	90.3<br>(72.4, 112.6)	191.9<br>(155.2, 238.5)	1.13<br>(1.00, 1.26)	2.3<br>(1.9, 2.9)	2.3<br>(1.8, 2.8)	-0.03<br>(-0.08, 0.01)
Turkmenistan	61.0<br>(49.9, 75.7)	103.2<br>(84.4, 128.2)	0.69<br>(0.56, 0.81)	2.2<br>(1.8, 2.7)	2.2<br>(1.8, 2.6)	-0.04<br>(-0.09, 0.01)
Uzbekistan	387.6<br>(317.0, 476.1)	680.4<br>(554.7, 842.8)	0.76<br>(0.61, 0.91)	2.5<br>(2.0, 3.0)	2.1<br>(1.7, 2.6)	-0.15<br>(-0.20, -0.09)
Central Europe	3455.7<br>(2820.6, 4227.7)	3520.7<br>(2863.7, 4311.3)	0.02<br>(-0.03, 0.07)	2.5<br>(2.1, 3.0)	2.3<br>(1.9, 2.8)	-0.09<br>(-0.10, -0.07)
Albania	84.6<br>(68.4, 104.6)	90.5<br>(73.1, 111.5)	0.07<br>(-0.06, 0.21)	3.1<br>(2.5, 3.8)	2.7<br>(2.2, 3.3)	-0.13<br>(-0.18, -0.09)
Bosnia and Herzegovina	140.0<br>(114.3, 171.7)	121.3<br>(98.3, 149.9)	-0.13<br>(-0.21, -0.05)	3.1<br>(2.5, 3.7)	2.8<br>(2.3, 3.4)	-0.09<br>(-0.14, -0.05)
Bulgaria	255.2<br>(208.3, 311.9)	181.0<br>(146.2, 222.2)	-0.29<br>(-0.35, -0.22)	2.5<br>(2.0, 3.0)	1.9<br>(1.6, 2.4)	-0.22<br>(-0.26, -0.17)
Croatia	163.0<br>(131.2, 199.8)	149.7<br>(120.9, 183.4)	-0.08<br>(-0.15, 0.00)	2.9<br>(2.4, 3.6)	2.6<br>(2.1, 3.1)	-0.11<br>(-0.15, -0.07)
Czechia	349.8<br>(285.7, 424.8)	374.9<br>(302.7, 459.1)	0.07<br>(0.00, 0.15)	2.9<br>(2.4, 3.5)	2.6<br>(2.2, 3.2)	-0.11<br>(-0.15, -0.07)
Hungary	408.8<br>(334.3, 500.6)	397.0<br>(318.5, 484.0)	-0.03<br>(-0.09, 0.04)	3.3<br>(2.8, 4.0)	3.0<br>(2.5, 3.6)	-0.09<br>(-0.14, -0.04)

Montenegro	16.0<br>(13.1, 19.5)	18.3<br>(14.7, 22.5)	0.14<br>(0.06, 0.24)	2.5<br>(2.1, 3.0)	2.4<br>(2.0, 2.9)	-0.05<br>(-0.09, 0.00)
North Macedonia	52.1<br>(42.4, 63.7)	63.3<br>(51.0, 78.3)	0.22<br>(0.13, 0.32)	2.6<br>(2.1, 3.1)	2.3<br>(1.9, 2.8)	-0.11<br>(-0.15, -0.06)
Poland	949.3<br>(777.1, 1161.3)	1091.4<br>(890.4, 1342.7)	0.15<br>(0.09, 0.21)	2.3<br>(1.9, 2.8)	2.2<br>(1.8, 2.6)	-0.06<br>(-0.07, -0.04)
Romania	561.6<br>(455.9, 686.2)	481.0<br>(389.0, 592.3)	-0.14<br>(-0.21, -0.08)	2.2<br>(1.8, 2.7)	1.9<br>(1.5, 2.3)	-0.14<br>(-0.19, -0.10)
Serbia	207.7<br>(167.7, 258.3)	242.1<br>(192.7, 300.0)	0.17<br>(0.09, 0.26)	2.0<br>(1.6, 2.4)	2.1<br>(1.7, 2.5)	0.06<br>(0.01, 0.12)
Slovakia	147.1<br>(120.6, 180.0)	185.1<br>(149.3, 232.1)	0.26<br>(0.17, 0.37)	2.6<br>(2.2, 3.2)	2.6<br>(2.2, 3.2)	0.01<br>(-0.05, 0.08)
Slovenia	65.5<br>(53.7, 79.9)	73.8<br>(60.3, 91.4)	0.13<br>(0.04, 0.22)	2.9<br>(2.4, 3.5)	2.6<br>(2.1, 3.1)	-0.12<br>(-0.15, -0.08)
Central Latin America	3262.3<br>(2678.7, 3991.3)	8421.5<br>(6855.6, 10382.6)	1.58<br>(1.40, 1.77)	2.7<br>(2.2, 3.3)	3.2<br>(2.6, 4.0)	0.20<br>(0.17, 0.22)
Colombia	731.1<br>(600.3, 886.6)	1816.6<br>(1473.8, 2240.8)	1.48<br>(1.28, 1.69)	3.0<br>(2.4, 3.6)	3.4<br>(2.8, 4.1)	0.14<br>(0.08, 0.20)
Costa Rica	68.8<br>(56.0, 85.1)	189.3<br>(152.9, 233.1)	1.75<br>(1.52, 1.99)	2.9<br>(2.4, 3.6)	3.6<br>(2.9, 4.4)	0.23<br>(0.17, 0.30)
El Salvador	95.8<br>(78.7, 116.7)	226.8<br>(185.6, 281.4)	1.37<br>(1.18, 1.57)	2.5<br>(2.0, 3.0)	3.6<br>(2.9, 4.5)	0.47<br>(0.36, 0.57)
Guatemala	123.4<br>(101.3, 153.0)	393.0<br>(319.4, 485.6)	2.19<br>(1.96, 2.42)	2.3<br>(1.9, 2.8)	2.9<br>(2.4, 3.6)	0.28<br>(0.20, 0.37)
Honduras	73.1<br>(59.9, 90.3)	245.8<br>(201.4, 303.8)	2.36<br>(2.13, 2.58)	2.4<br>(1.9, 2.9)	3.0<br>(2.4, 3.6)	0.26<br>(0.18, 0.34)
Mexico	1633.5<br>(1345.4, 2012.0)	4287.3<br>(3482.5, 5273.3)	1.62<br>(1.43, 1.82)	2.6<br>(2.1, 3.2)	3.2<br>(2.6, 3.9)	0.24<br>(0.21, 0.27)
Nicaragua	58.6<br>(48.1, 73.2)	175.0<br>(142.4, 214.3)	1.99<br>(1.76, 2.24)	2.3<br>(1.9, 2.9)	2.9<br>(2.4, 3.6)	0.25<br>(0.18, 0.34)
Panama	53.7<br>(43.8, 65.5)	152.2<br>(122.7, 187.7)	1.83<br>(1.65, 2.04)	2.8<br>(2.3, 3.4)	3.5<br>(2.8, 4.3)	0.23<br>(0.17, 0.30)

Venezuela (Bolivarian Republic of)	424.3 (344.0, 518.1)	935.4 (757.5, 1155.7)	1.20 (1.01, 1.43)	3.0 (2.5, 3.7)	3.2 (2.6, 3.9)	0.05 (0.00, 0.11)
Central Sub-Saharan Africa	972.0 (795.5, 1205.9)	1693.4 (1377.9, 2093.5)	0.74 (0.65, 0.85)	2.8 (2.3, 3.4)	1.9 (1.5, 2.2)	-0.34 (-0.37, -0.30)
Angola	121.4 (96.9, 150.1)	330.9 (268.9, 408.3)	1.72 (1.54, 1.95)	1.9 (1.5, 2.3)	1.6 (1.3, 2.0)	-0.14 (-0.19, -0.07)
Central African Republic	32.7 (26.3, 40.1)	61.0 (48.6, 75.8)	0.87 (0.76, 1.00)	1.9 (1.5, 2.3)	1.7 (1.4, 2.0)	-0.10 (-0.15, -0.04)
Congo	40.8 (33.1, 50.2)	87.6 (71.2, 108.1)	1.15 (1.03, 1.31)	2.6 (2.1, 3.1)	2.1 (1.7, 2.6)	-0.17 (-0.21, -0.12)
Democratic Republic of the Congo	751.2 (615.6, 933.8)	1165.9 (943.9, 1452.2)	0.55 (0.43, 0.68)	3.1 (2.5, 3.9)	1.9 (1.5, 2.3)	-0.38 (-0.43, -0.33)
Equatorial Guinea	5.2 (4.2, 6.3)	18.2 (14.7, 23.0)	2.54 (2.29, 2.79)	1.9 (1.5, 2.2)	1.9 (1.5, 2.3)	0.00 (-0.06, 0.06)
Gabon	20.7 (17.0, 25.1)	29.7 (23.9, 36.8)	0.44 (0.34, 0.54)	2.8 (2.3, 3.4)	2.1 (1.7, 2.5)	-0.28 (-0.32, -0.23)
East Asia	22867.5 (18512.9, 28350.6)	42486.0 (33929.8, 53043.2)	0.86 (0.69, 1.02)	2.1 (1.7, 2.5)	2.2 (1.8, 2.7)	0.08 (0.06, 0.09)
China mainland	22027.9 (17840.2, 27321.5)	41135.2 (32838.9, 51357.3)	0.87 (0.69, 1.03)	2.1 (1.7, 2.5)	2.2 (1.8, 2.8)	0.08 (0.07, 0.10)
Democratic People's Republic of Korea	385.7 (310.7, 477.5)	601.8 (479.0, 752.3)	0.56 (0.45, 0.69)	2.0 (1.6, 2.5)	1.9 (1.6, 2.4)	-0.05 (-0.11, 0.01)
Taiwan (Province of China mainland)	453.9 (367.7, 559.0)	749.0 (598.6, 928.8)	0.65 (0.48, 0.84)	2.4 (1.9, 2.9)	2.3 (1.8, 2.8)	-0.05 (-0.10, 0.00)
Eastern Europe	8178.8 (6670.5, 10049.3)	7686.2 (6263.7, 9489.3)	-0.06 (-0.10, -0.03)	3.2 (2.6, 3.9)	2.8 (2.3, 3.4)	-0.12 (-0.13, -0.10)
Belarus	433.5 (355.8, 530.7)	384.5 (310.6, 480.1)	-0.11 (-0.17, -0.05)	3.7 (3.0, 4.5)	3.1 (2.5, 3.8)	-0.16 (-0.21, -0.11)
Estonia	65.6 (53.9, 80.9)	56.6 (45.3, 70.3)	-0.14 (-0.20, -0.08)	3.6 (3.0, 4.4)	3.1 (2.5, 3.7)	-0.14 (-0.19, -0.10)
Latvia	109.7 (89.7, 134.1)	87.0 (70.6, 107.6)	-0.21 (-0.26, -0.14)	3.5 (2.9, 4.2)	3.2 (2.6, 3.9)	-0.09 (-0.13, -0.04)

Lithuania	137.6<br>(113.3, 167.8)	115.8<br>(94.0, 141.9)	-0.16<br>(-0.22, -0.09)	3.3<br>(2.8, 4.0)	2.9<br>(2.4, 3.6)	-0.12<br>(-0.16, -0.07)
Republic of Moldova	142.0<br>(117.3, 173.5)	138.8<br>(112.2, 171.6)	-0.02<br>(-0.09, 0.04)	3.2<br>(2.6, 3.9)	2.9<br>(2.4, 3.6)	-0.08<br>(-0.12, -0.04)
Russian Federation	5368.1<br>(4376.6, 6595.9)	5452.5<br>(4431.7, 6731.3)	0.02<br>(-0.02, 0.05)	3.2<br>(2.6, 3.9)	2.9<br>(2.4, 3.5)	-0.10<br>(-0.11, -0.08)
Ukraine	1922.2<br>(1562.6, 2366.9)	1450.9<br>(1164.5, 1795.2)	-0.25<br>(-0.29, -0.19)	3.1<br>(2.5, 3.8)	2.5<br>(2.1, 3.1)	-0.19<br>(-0.23, -0.15)
Eastern Sub-Saharan Africa	2874.6<br>(2358.7, 3535.6)	5907.5<br>(4822.1, 7343.5)	1.06<br>(1.01, 1.10)	2.4<br>(1.9, 2.9)	2.1<br>(1.7, 2.5)	-0.13<br>(-0.15, -0.12)
Burundi	88.8<br>(71.9, 110.1)	145.7<br>(116.5, 182.3)	0.64<br>(0.52, 0.75)	2.5<br>(2.0, 3.1)	1.7<br>(1.4, 2.1)	-0.32<br>(-0.37, -0.28)
Comoros	6.0<br>(4.8, 7.4)	10.8<br>(8.7, 13.3)	0.8<br>(0.65, 0.96)	2.0<br>(1.6, 2.4)	1.7<br>(1.4, 2.1)	-0.15<br>(-0.21, -0.09)
Djibouti	4.9<br>(4.0, 6.1)	19.4<br>(15.7, 23.9)	2.99<br>(2.63, 3.28)	1.8<br>(1.5, 2.2)	1.9<br>(1.6, 2.4)	0.05<br>(-0.02, 0.12)
Eritrea	45.2<br>(36.8, 56.0)	82.8<br>(66.5, 104.0)	0.83<br>(0.70, 0.98)	2.2<br>(1.8, 2.6)	1.8<br>(1.4, 2.2)	-0.19<br>(-0.25, -0.13)
Ethiopia	786.7<br>(647.7, 971.7)	1856.6<br>(1521.3, 2307.1)	1.36<br>(1.28, 1.44)	2.5<br>(2.0, 3.0)	2.5<br>(2.1, 3.1)	0.02<br>(-0.01, 0.05)
Kenya	349.5<br>(286.3, 432.3)	926.7<br>(761.5, 1138.6)	1.65<br>(1.55, 1.75)	2.5<br>(2.0, 3.0)	2.6<br>(2.1, 3.1)	0.04<br>(0.02, 0.06)
Madagascar	176.8<br>(144.8, 220.2)	380.3<br>(304.1, 479.4)	1.15<br>(1.00, 1.32)	2.3<br>(1.9, 2.8)	2.0<br>(1.6, 2.4)	-0.15<br>(-0.20, -0.09)
Malawi	131.0<br>(104.9, 160.4)	202.9<br>(163.1, 253.0)	0.55<br>(0.45, 0.67)	2.1<br>(1.7, 2.6)	1.6<br>(1.3, 1.9)	-0.25<br>(-0.30, -0.20)
Mozambique	177.8<br>(145.2, 219.5)	331.4<br>(268.2, 411.6)	0.86<br>(0.72, 1.00)	2.0<br>(1.6, 2.5)	1.7<br>(1.4, 2.1)	-0.15<br>(-0.21, -0.08)
Rwanda	120.4<br>(97.1, 148.6)	165.0<br>(133.3, 206.3)	0.37<br>(0.27, 0.50)	2.7<br>(2.2, 3.3)	1.7<br>(1.4, 2.1)	-0.37<br>(-0.42, -0.32)
Somalia	96.4<br>(76.8, 120.0)	215.2<br>(171.4, 270.7)	1.23<br>(1.08, 1.39)	2.1<br>(1.7, 2.5)	1.7<br>(1.4, 2.1)	-0.17<br>(-0.22, -0.11)

South Sudan	76.5<br>(62.2, 95.3)	117.7<br>(95.1, 146.8)	0.54<br>(0.44, 0.65)	2.0<br>(1.6, 2.5)	1.8<br>(1.5, 2.3)	-0.07<br>(-0.12, -0.01)
Uganda	320.6<br>(256.4, 399.7)	529.6<br>(432.4, 654.2)	0.65<br>(0.55, 0.77)	3.0<br>(2.4, 3.8)	2.0<br>(1.6, 2.4)	-0.34<br>(-0.38, -0.29)
United Republic of Tanzania	386.6<br>(317.6, 471.0)	715.3<br>(575.5, 881.8)	0.85<br>(0.73, 0.98)	2.3<br>(1.9, 2.9)	1.8<br>(1.4, 2.2)	-0.23<br>(-0.28, -0.18)
Zambia	105.5<br>(85.7, 130.3)	202.8<br>(165.3, 252.0)	0.92<br>(0.77, 1.10)	2.2<br>(1.8, 2.7)	1.6<br>(1.3, 2.0)	-0.27<br>(-0.33, -0.21)
High-income Asia Pacific	6368.8<br>(5211.6, 7811.9)	9167.3<br>(7427.0, 11361.8)	0.44<br>(0.33, 0.56)	3.2<br>(2.7, 4.0)	3.0<br>(2.5, 3.7)	-0.07<br>(-0.09, -0.06)
Brunei Darussalam	3.8<br>(3.1, 4.8)	9.0<br>(7.2, 11.2)	1.35<br>(1.13, 1.58)	2.1<br>(1.7, 2.5)	2.0<br>(1.6, 2.5)	-0.03<br>(-0.08, 0.01)
Japan	5141.1<br>(4190.4, 6321.7)	6753.8<br>(5460.2, 8398.5)	0.31<br>(0.21, 0.43)	3.3<br>(2.7, 4.1)	3.1<br>(2.5, 3.8)	-0.07<br>(-0.08, -0.06)
Republic of Korea	1152.3<br>(954.3, 1431)	2233.8<br>(1798.2, 2797.2)	0.94<br>(0.72, 1.18)	3.0<br>(2.5, 3.7)	2.9<br>(2.4, 3.6)	-0.03<br>(-0.09, 0.01)
Singapore	71.6<br>(58.0, 89.7)	170.7<br>(136.9, 212.3)	1.38<br>(1.13, 1.63)	2.5<br>(2.0, 3.1)	2.2<br>(1.8, 2.8)	-0.10<br>(-0.16, -0.05)
High-income North America	5984.1<br>(4856.1, 7324.6)	8625.4<br>(6917.8, 10745.0)	0.44<br>(0.37, 0.51)	1.9<br>(1.5, 2.3)	1.7<br>(1.4, 2.1)	-0.08<br>(-0.09, -0.06)
Canada	661.6<br>(533.3, 810.1)	934.9<br>(749.1, 1164.4)	0.41<br>(0.29, 0.54)	2.1<br>(1.7, 2.6)	1.8<br>(1.4, 2.2)	-0.18<br>(-0.22, -0.13)
Greenland	0.8<br>(0.6, 0.9)	0.9<br>(0.7, 1.1)	0.15<br>(0.02, 0.30)	1.6<br>(1.3, 2.0)	1.3<br>(1.1, 1.6)	-0.18<br>(-0.22, -0.13)
United States of America	5321.6<br>(4318.4, 6522.4)	7689.6<br>(6171.6, 9562.5)	0.44<br>(0.38, 0.51)	1.8<br>(1.5, 2.3)	1.7<br>(1.4, 2.1)	-0.07<br>(-0.08, -0.05)
North Africa and Middle East	4967.6<br>(4055.9, 6153.2)	11590.8<br>(9389.5, 14435.6)	1.33<br>(1.20, 1.46)	2.0<br>(1.6, 2.5)	2.0<br>(1.6, 2.5)	0.00<br>(-0.02, 0.02)
Afghanistan	109.8<br>(89.6, 134.0)	277.8<br>(224.6, 343.8)	1.53<br>(1.25, 1.81)	1.4<br>(1.2, 1.8)	1.5<br>(1.2, 1.8)	0.02<br>(-0.04, 0.09)
Algeria	307.7<br>(251.0, 379.6)	782.1<br>(631.4, 979.0)	1.54<br>(1.30, 1.78)	1.7<br>(1.4, 2.1)	1.9<br>(1.5, 2.3)	0.08<br>(0.02, 0.14)

Bahrain	7.3<br>(5.9, 9.3)	28.3<br>(22.5, 35.8)	2.89<br>(2.50, 3.39)	1.9<br>(1.5, 2.2)	1.8<br>(1.5, 2.3)	-0.01<br>(-0.06, 0.04)
Egypt	1119.6<br>(915.2, 1402.2)	1547.4<br>(1262.2, 1927.2)	0.38<br>(0.29, 0.48)	2.7<br>(2.2, 3.3)	1.7<br>(1.4, 2.1)	-0.36<br>(-0.40, -0.32)
Iran (Islamic Republic of)	807.4<br>(659.6, 992.3)	1903.6<br>(1532.7, 2361.2)	1.36<br>(1.17, 1.55)	2.1<br>(1.7, 2.5)	2.1<br>(1.7, 2.6)	0.03<br>(0.01, 0.05)
Iraq	210.8<br>(173.3, 260.9)	640.3<br>(524.6, 798.2)	2.04<br>(1.81, 2.28)	1.7<br>(1.4, 2.1)	1.9<br>(1.5, 2.3)	0.09<br>(0.03, 0.16)
Jordan	53.4<br>(43.5, 66.5)	242.8<br>(197.1, 299.3)	3.54<br>(3.21, 3.93)	2.2<br>(1.8, 2.7)	2.3<br>(1.8, 2.8)	0.02<br>(-0.03, 0.08)
Kuwait	27.2<br>(21.6, 34.4)	105.9<br>(82.7, 135.2)	2.90<br>(2.55, 3.30)	2.1<br>(1.7, 2.5)	2.2<br>(1.7, 2.6)	0.05<br>(0.00, 0.10)
Lebanon	52.8<br>(43.0, 64.9)	123.0<br>(99.2, 153.0)	1.33<br>(1.20, 1.48)	2.1<br>(1.7, 2.5)	2.1<br>(1.7, 2.6)	0.01<br>(-0.04, 0.06)
Libya	63.9<br>(52.2, 78.8)	144.1<br>(115.3, 181.0)	1.25<br>(1.05, 1.47)	2.2<br>(1.8, 2.7)	2.1<br>(1.7, 2.6)	-0.05<br>(-0.10, 0.00)
Morocco	344.1<br>(281.7, 426.1)	677.6<br>(545.8, 846.3)	0.97<br>(0.80, 1.13)	1.8<br>(1.4, 2.2)	1.8<br>(1.5, 2.2)	0.02<br>(-0.04, 0.08)
Oman	27.2<br>(21.9, 34.2)	83.3<br>(67.2, 107.5)	2.06<br>(1.84, 2.29)	2.0<br>(1.6, 2.4)	2.0<br>(1.6, 2.5)	0.01<br>(-0.04, 0.07)
Palestine	28.2<br>(22.9, 34.2)	75.5<br>(61.3, 92.9)	1.67<br>(1.50, 1.89)	2.2<br>(1.8, 2.7)	1.9<br>(1.5, 2.3)	-0.13<br>(-0.18, -0.07)
Qatar	6.6<br>(5.2, 8.5)	54.3<br>(42.4, 69.8)	7.17<br>(6.55, 7.85)	1.9<br>(1.5, 2.3)	1.9<br>(1.5, 2.3)	0.02<br>(-0.04, 0.07)
Saudi Arabia	178.9<br>(144.2, 224.2)	672.7<br>(535.3, 842.3)	2.76<br>(2.39, 3.12)	1.6<br>(1.3, 2.0)	1.9<br>(1.6, 2.3)	0.18<br>(0.11, 0.25)
Sudan	237.0<br>(193.5, 292.7)	531.0<br>(438.5, 667.3)	1.24<br>(1.11, 1.38)	1.7<br>(1.4, 2.2)	1.7<br>(1.4, 2.0)	-0.03<br>(-0.09, 0.03)
Syrian Arab Republic	156.5<br>(126.9, 194.0)	279.9<br>(227.0, 345.6)	0.79<br>(0.58, 1.01)	1.9<br>(1.5, 2.3)	2.0<br>(1.6, 2.4)	0.05<br>(-0.01, 0.11)
Tunisia	141.5<br>(116.4, 174.3)	306.4<br>(246.0, 379.7)	1.16<br>(0.98, 1.36)	2.2<br>(1.7, 2.6)	2.3<br>(1.9, 2.9)	0.08<br>(0.02, 0.14)

Türkiye	857.9	2423.4	1.82	1.9	2.6	0.40
	(704.5, 1075.7)	(1941.9, 3032.6)	(1.61, 2.05)	(1.5, 2.3)	(2.1, 3.2)	(0.32, 0.48)
United Arab Emirates	24.5	175.2	6.16	1.8	1.7	-0.05
	(19.4, 31.5)	(133.9, 229.6)	(5.10, 7.34)	(1.4, 2.2)	(1.4, 2.0)	(-0.10, 0.01)
Yemen	202.7	505.5	1.49	2.5	2.1	-0.14
	(166.7, 249.1)	(413.2, 634.1)	(1.33, 1.65)	(2.0, 3.0)	(1.7, 2.6)	(-0.19, -0.09)
Oceania	91.5	196.3	1.14	2.0	1.8	-0.09
	(74.6, 112.5)	(158.8, 241.3)	(1.03, 1.26)	(1.6, 2.4)	(1.4, 2.2)	(-0.13, -0.06)
American Samoa	0.8	1.0	0.33	2.1	2.0	-0.06
	(0.6, 1.0)	(0.8, 1.2)	(0.20, 0.45)	(1.7, 2.6)	(1.6, 2.5)	(-0.11, -0.01)
Cook Islands	0.4	0.5	0.27	2.3	2.2	-0.05
	(0.3, 0.4)	(0.4, 0.6)	(0.14, 0.41)	(1.9, 2.8)	(1.8, 2.7)	(-0.11, -0.01)
Fiji	12.8	17.2	0.33	2.2	1.9	-0.13
	(10.5, 15.9)	(13.9, 21.1)	(0.23, 0.46)	(1.8, 2.7)	(1.6, 2.4)	(-0.19, -0.08)
Guam	2.5	3.8	0.53	2.2	2.1	-0.06
	(2.0, 3.1)	(3.1, 4.7)	(0.36, 0.71)	(1.8, 2.7)	(1.7, 2.5)	(-0.12, 0.00)
Kiribati	1.0	1.6	0.68	1.8	1.7	-0.08
	(0.8, 1.2)	(1.3, 2.1)	(0.57, 0.79)	(1.5, 2.2)	(1.3, 2.0)	(-0.14, -0.03)
Marshall Islands	0.5	0.8	0.54	1.8	1.6	-0.08
	(0.4, 0.6)	(0.6, 1.0)	(0.39, 0.69)	(1.4, 2.2)	(1.3, 2.0)	(-0.13, -0.02)
Micronesia (Federated	1.3	1.5	0.16	1.8	1.7	-0.09
States of)	(1.1, 1.6)	(1.3, 1.9)	(0.06, 0.25)	(1.5, 2.2)	(1.4, 2.0)	(-0.14, -0.04)
Nauru	0.1	0.1	0.11	1.7	1.6	-0.05
	(0.1, 0.2)	(0.1, 0.2)	(0.04, 0.17)	(1.4, 2.1)	(1.3, 2.0)	(-0.10, 0.01)
Niue	0.0	0.0	-0.19	2.2	2.0	-0.09
	(0.0, 0.1)	(0.0, 0.0)	(-0.26, -0.12)	(1.8, 2.7)	(1.6, 2.4)	(-0.15, -0.04)
Northern Mariana Islands	0.8	1.1	0.38	2.2	2.1	-0.06
	(0.7, 1.1)	(0.9, 1.4)	(0.19, 0.59)	(1.8, 2.7)	(1.7, 2.6)	(-0.11, -0.01)
Palau	0.3	0.4	0.52	2.1	1.8	-0.10
	(0.2, 0.3)	(0.3, 0.5)	(0.32, 0.71)	(1.6, 2.5)	(1.5, 2.2)	(-0.15, -0.05)
Papua New Guinea	54.7	140.9	1.58	1.9	1.8	-0.07
	(44.6, 67.2)	(113.2, 173.9)	(1.41, 1.75)	(1.6, 2.4)	(1.4, 2.2)	(-0.13, -0.02)

Samoa	2.8<br>(2.3, 3.5)	3.7<br>(3.1, 4.5)	0.31<br>(0.23, 0.40)	2.4<br>(1.9, 2.9)	2.1<br>(1.7, 2.6)	-0.12<br>(-0.16, -0.06)
Solomon Islands	4.1<br>(3.3, 5.0)	8.8<br>(7.2, 10.9)	1.16<br>(1.02, 1.31)	1.8<br>(1.5, 2.2)	1.7<br>(1.4, 2.0)	-0.07<br>(-0.12, -0.01)
Tokelau	0.0<br>(0.0, 0.0)	0.0<br>(0.0, 0.0)	0.06<br>(-0.01, 0.13)	1.9<br>(1.5, 2.3)	2.0<br>(1.6, 2.4)	0.04<br>(-0.02, 0.11)
Tonga	1.7<br>(1.4, 2.0)	1.9<br>(1.6, 2.4)	0.16<br>(0.10, 0.24)	2.3<br>(1.9, 2.8)	2.1<br>(1.7, 2.6)	-0.07<br>(-0.11, -0.01)
Tuvalu	0.2<br>(0.1, 0.2)	0.2<br>(0.2, 0.2)	0.26<br>(0.18, 0.34)	2.0<br>(1.6, 2.4)	1.7<br>(1.4, 2.1)	-0.14<br>(-0.19, -0.08)
Vanuatu	1.7<br>(1.4, 2.1)	3.9<br>(3.1, 4.7)	1.25<br>(1.11, 1.40)	1.7<br>(1.3, 2.0)	1.6<br>(1.3, 1.9)	-0.06<br>(-0.10, -0.01)
South Asia	12896.6<br>(10582.4, 15994.3)	29560.9<br>(23835.1, 36926.6)	1.29<br>(1.20, 1.38)	1.6<br>(1.3, 1.9)	1.7<br>(1.4, 2.1)	0.08<br>(0.06, 0.09)
Bangladesh	918.2<br>(749.5, 1147.3)	2842.4<br>(2308.8, 3540.7)	2.10<br>(1.85, 2.38)	1.3<br>(1.0, 1.6)	1.8<br>(1.5, 2.2)	0.42<br>(0.33, 0.52)
Bhutan	7.9<br>(6.4, 9.7)	11.9<br>(9.8, 14.8)	0.52<br>(0.38, 0.65)	1.9<br>(1.6, 2.3)	1.6<br>(1.3, 2.0)	-0.14<br>(-0.19, -0.08)
India	10592.0<br>(8714.5, 13169.4)	23741.7<br>(19176.4, 29559.1)	1.24<br>(1.14, 1.34)	1.6<br>(1.3, 2.0)	1.7<br>(1.4, 2.1)	0.07<br>(0.05, 0.09)
Nepal	204.3<br>(166.0, 254.3)	445.2<br>(362.0, 545.4)	1.18<br>(1.04, 1.32)	1.5<br>(1.2, 1.8)	1.6<br>(1.3, 1.9)	0.07<br>(0.00, 0.14)
Pakistan	1174.3<br>(958.1, 1432.9)	2519.7<br>(2035.5, 3133.0)	1.15<br>(1.04, 1.25)	1.5<br>(1.2, 1.8)	1.4<br>(1.1, 1.7)	-0.08<br>(-0.12, -0.04)
Southeast Asia	6366.0<br>(5233.7, 7851.6)	13615.9<br>(11019.3, 16957.0)	1.14<br>(1.00, 1.28)	1.8<br>(1.4, 2.2)	1.9<br>(1.5, 2.3)	0.07<br>(0.06, 0.09)
Cambodia	109.7<br>(89.7, 136.7)	245.1<br>(196.8, 301.0)	1.23<br>(1.05, 1.39)	1.6<br>(1.3, 1.9)	1.6<br>(1.3, 1.9)	0.00<br>(-0.06, 0.06)
Indonesia	2130.9<br>(1732.3, 2636.8)	4923.2<br>(3970.5, 6153.6)	1.31<br>(1.17, 1.46)	1.5<br>(1.2, 1.8)	1.7<br>(1.4, 2.1)	0.17<br>(0.14, 0.19)
Lao People's Democratic Republic	50.5<br>(41.2, 63.0)	126.5<br>(102.6, 157.3)	1.50<br>(1.34, 1.67)	1.7<br>(1.4, 2.1)	2.0<br>(1.6, 2.4)	0.16<br>(0.10, 0.24)

Malaysia	257.4<br>(211.4, 322.1)	594.1<br>(479.1, 737.2)	1.31<br>(1.13, 1.48)	1.9<br>(1.5, 2.3)	1.9<br>(1.5, 2.3)	-0.01<br>(-0.06, 0.05)
Maldives	2.6<br>(2.1, 3.2)	10.7<br>(8.5, 13.6)	3.20<br>(2.76, 3.64)	1.7<br>(1.4, 2.1)	2.1<br>(1.7, 2.6)	0.18<br>(0.11, 0.25)
Mauritius	31.5<br>(25.7, 39.5)	51.6<br>(41.6, 63.5)	0.64<br>(0.46, 0.84)	3.3<br>(2.7, 4.0)	3.2<br>(2.6, 4.0)	-0.01<br>(-0.06, 0.05)
Myanmar	485.4<br>(394.5, 595.3)	858.2<br>(680.9, 1058.5)	0.77<br>(0.63, 0.94)	1.6<br>(1.3, 1.9)	1.6<br>(1.3, 1.9)	0.01<br>(-0.05, 0.07)
Philippines	1047.0<br>(859.5, 1291.0)	2238.5<br>(1836.2, 2775.8)	1.14<br>(1.03, 1.23)	2.3<br>(1.9, 2.8)	2.2<br>(1.8, 2.7)	-0.05<br>(-0.06, -0.04)
Seychelles	1.2<br>(0.9, 1.4)	2.2<br>(1.8, 2.7)	0.88<br>(0.73, 1.06)	1.8<br>(1.5, 2.2)	1.8<br>(1.5, 2.3)	0.03<br>(-0.02, 0.09)
Sri Lanka	347.3<br>(285.1, 427.3)	645.8<br>(513.8, 798.3)	0.86<br>(0.69, 1.04)	2.4<br>(1.9, 2.9)	2.5<br>(2.0, 3.1)	0.06<br>(0.00, 0.12)
Thailand	921.7<br>(747.4, 1144.7)	1891.9<br>(1513.4, 2392.0)	1.05<br>(0.81, 1.31)	1.9<br>(1.6, 2.4)	2.1<br>(1.7, 2.6)	0.08<br>(0.03, 0.14)
Timor-Leste	9.0<br>(7.3, 11.4)	19.7<br>(15.9, 24.1)	1.19<br>(1.02, 1.38)	1.7<br>(1.4, 2.1)	1.8<br>(1.5, 2.2)	0.05<br>(-0.01, 0.10)
Viet Nam	962.5<br>(786.9, 1190.9)	1989.5<br>(1591.3, 2490.7)	1.07<br>(0.87, 1.26)	1.8<br>(1.5, 2.3)	1.9<br>(1.5, 2.3)	0.02<br>(-0.04, 0.07)
Southern Latin America	1280.9<br>(1039.5, 1574.4)	2227.3<br>(1800.2, 2760.0)	0.74<br>(0.67, 0.81)	2.7<br>(2.2, 3.3)	2.8<br>(2.3, 3.5)	0.05<br>(0.01, 0.08)
Uruguay	87.9<br>(71.5, 108.2)	98.6<br>(79.5, 121.8)	0.12<br>(0.05, 0.19)	2.5<br>(2.0, 3.1)	2.2<br>(1.8, 2.8)	-0.10<br>(-0.15, -0.05)
Argentina	813.5<br>(655.8, 999.0)	1370.6<br>(1107.8, 1702.3)	0.68<br>(0.60, 0.78)	2.5<br>(2.0, 3.1)	2.7<br>(2.1, 3.3)	0.06<br>(0.01, 0.11)
Chile	379.5<br>(311.1, 466.4)	758.0<br>(610.7, 930.8)	1.00<br>(0.85, 1.15)	3.3<br>(2.7, 4.0)	3.3<br>(2.7, 4.0)	0.01<br>(-0.04, 0.07)
Southern Sub-Saharan Africa	795.8<br>(658.4, 966.6)	1596.2<br>(1287.5, 1944.8)	1.01<br>(0.90, 1.10)	2.1<br>(1.7, 2.6)	2.3<br>(1.9, 2.8)	0.08<br>(0.05, 0.10)
Botswana	19.6<br>(16.0, 24.1)	39.2<br>(31.7, 48.6)	1.00<br>(0.83, 1.17)	2.3<br>(1.8, 2.7)	1.9<br>(1.6, 2.4)	-0.14<br>(-0.19, -0.08)

Eswatini	8.4<br>(6.8, 10.3)	14.7<br>(11.8, 18.0)	0.75<br>(0.63, 0.90)	1.7<br>(1.4, 2.1)	1.7<br>(1.4, 2.1)	0.01<br>(-0.05, 0.09)
Lesotho	18.5<br>(15.2, 22.4)	24.7<br>(20.3, 30.4)	0.34<br>(0.25, 0.43)	1.7<br>(1.4, 2.1)	1.7<br>(1.4, 2.1)	0.01<br>(-0.06, 0.07)
Namibia	26.3<br>(21.4, 32.5)	51.0<br>(42.1, 61.8)	0.94<br>(0.81, 1.08)	2.7<br>(2.2, 3.4)	2.7<br>(2.2, 3.2)	-0.03<br>(-0.08, 0.04)
South Africa	605.4<br>(499.3, 737.4)	1266.7<br>(1021.3, 1543.7)	1.09<br>(0.97, 1.20)	2.2<br>(1.8, 2.7)	2.4<br>(1.9, 2.9)	0.09<br>(0.06, 0.12)
Zimbabwe	117.6<br>(96.2, 144.6)	199.9<br>(162.0, 250.1)	0.70<br>(0.57, 0.82)	1.8<br>(1.4, 2.2)	1.9<br>(1.5, 2.3)	0.04<br>(-0.03, 0.11)
Tropical Latin America	2885.3<br>(2356.8, 3541.2)	6238.6<br>(5019.8, 7679.4)	1.16<br>(1.01, 1.31)	2.3<br>(1.9, 2.9)	2.5<br>(2.0, 3.0)	0.06<br>(0.04, 0.07)
Brazil	2810.8<br>(2296.1, 3451.1)	6077.9<br>(4890.5, 7486.9)	1.16<br>(1.00, 1.31)	2.3<br>(1.9, 2.9)	2.5<br>(2.0, 3.0)	0.06<br>(0.04, 0.08)
Paraguay	74.5<br>(60.7, 91.0)	160.7<br>(130.3, 196.4)	1.16<br>(1.03, 1.31)	2.5<br>(2.0, 3.1)	2.4<br>(1.9, 2.9)	-0.04<br>(-0.10, 0.01)
Western Europe	15675.2<br>(12581.7, 19293.6)	23620.9<br>(19120.7, 29197.3)	0.51<br>(0.45, 0.56)	3.2<br>(2.6, 4.0)	3.6<br>(2.9, 4.4)	0.11<br>(0.09, 0.13)
Andorra	2.1<br>(1.7, 2.6)	4.6<br>(3.7, 5.7)	1.21<br>(1.06, 1.37)	3.5<br>(2.8, 4.2)	3.7<br>(3.0, 4.5)	0.06<br>(0.01, 0.12)
Austria	340.6<br>(273.3, 421.0)	481.0<br>(386.9, 595.2)	0.41<br>(0.33, 0.50)	3.4<br>(2.8, 4.2)	3.6<br>(2.9, 4.4)	0.05<br>(-0.01, 0.10)
Belgium	511.9<br>(412.2, 632.4)	719.5<br>(584.7, 900.0)	0.41<br>(0.33, 0.49)	4.0<br>(3.2, 4.9)	4.2<br>(3.5, 5.2)	0.07<br>(0.02, 0.12)
Cyprus	29.9<br>(24.2, 36.6)	76.4<br>(61.8, 93.0)	1.56<br>(1.41, 1.71)	3.6<br>(2.9, 4.4)	4.2<br>(3.5, 5.1)	0.18<br>(0.11, 0.24)
Denmark	206.0<br>(165.9, 254.8)	349.7<br>(282.2, 438.2)	0.70<br>(0.60, 0.81)	3.1<br>(2.5, 3.9)	4.0<br>(3.3, 5.0)	0.29<br>(0.22, 0.37)
Finland	197.2<br>(157.8, 244.3)	332.3<br>(268.1, 415.4)	0.69<br>(0.58, 0.81)	3.2<br>(2.6, 3.9)	3.8<br>(3.1, 4.6)	0.20<br>(0.14, 0.27)
France	2329.4<br>(1884.4, 2879.1)	4013.5<br>(3243.8, 4928.2)	0.72<br>(0.62, 0.83)	3.3<br>(2.7, 4.1)	4.1<br>(3.3, 5.0)	0.23<br>(0.17, 0.30)

Germany	3020.8<br>(2398.7, 3717.1)	4886.5<br>(3953.6, 6093.8)	0.62<br>(0.51, 0.72)	2.9<br>(2.3, 3.5)	3.6<br>(2.9, 4.4)	0.25<br>(0.18, 0.31)
Greece	403.5<br>(320.5, 502.8)	459.1<br>(370.0, 569.6)	0.14<br>(0.05, 0.22)	3.1<br>(2.5, 3.8)	2.9<br>(2.4, 3.5)	-0.06<br>(-0.11, -0.01)
Iceland	8.3<br>(6.7, 10.1)	14.8<br>(11.9, 18.4)	0.78<br>(0.67, 0.90)	3.1<br>(2.5, 3.8)	3.2<br>(2.6, 3.9)	0.04<br>(-0.01, 0.09)
Ireland	61.5<br>(50.0, 76.0)	123.5<br>(99.1, 154.2)	1.01<br>(0.88, 1.15)	1.6<br>(1.3, 2.0)	1.9<br>(1.5, 2.3)	0.18<br>(0.12, 0.26)
Israel	216.9<br>(177.5, 263.2)	464.2<br>(377.7, 563.8)	1.14<br>(1.03, 1.26)	4.5<br>(3.7, 5.5)	4.4<br>(3.6, 5.3)	-0.02<br>(-0.07, 0.03)
Italy	2891.7<br>(2328.9, 3574.1)	3881.8<br>(3154.3, 4755.0)	0.34<br>(0.29, 0.40)	3.9<br>(3.2, 4.8)	3.9<br>(3.2, 4.8)	0.02<br>(0.00, 0.03)
Luxembourg	14.0<br>(11.3, 17.1)	30.2<br>(24.5, 37.2)	1.15<br>(1.04, 1.31)	2.9<br>(2.4, 3.5)	3.4<br>(2.8, 4.2)	0.18<br>(0.12, 0.25)
Malta	12.9<br>(10.4, 15.9)	26.0<br>(21.0, 32.1)	1.02<br>(0.86, 1.19)	3.1<br>(2.5, 3.9)	3.7<br>(3.1, 4.6)	0.20<br>(0.12, 0.27)
Monaco	1.6<br>(1.3, 2.1)	2.4<br>(1.9, 3.0)	0.44<br>(0.36, 0.54)	3.4<br>(2.7, 4.2)	3.7<br>(3.0, 4.5)	0.09<br>(0.03, 0.15)
Netherlands	781.2<br>(633.7, 962.9)	1192.5<br>(950.7, 1473.3)	0.53<br>(0.43, 0.63)	4.4<br>(3.6, 5.4)	4.7<br>(3.8, 5.8)	0.07<br>(0.02, 0.12)
Norway	219.9<br>(177.7, 269.2)	303.2<br>(246.5, 375.5)	0.38<br>(0.34, 0.42)	4.0<br>(3.3, 5.0)	4.0<br>(3.3, 4.9)	-0.01<br>(-0.03, 0.01)
Portugal	263.5<br>(209.6, 328.0)	364.8<br>(292.9, 453.3)	0.38<br>(0.28, 0.49)	2.1<br>(1.7, 2.6)	2.1<br>(1.7, 2.6)	0.01<br>(-0.05, 0.07)
San Marino	1.1<br>(0.9, 1.3)	2.0<br>(1.6, 2.5)	0.86<br>(0.75, 0.99)	3.6<br>(2.9, 4.4)	3.9<br>(3.1, 4.7)	0.08<br>(0.02, 0.14)
Spain	1296.0<br>(1043.8, 1588.8)	1970.8<br>(1573.2, 2448.1)	0.52<br>(0.41, 0.64)	2.7<br>(2.2, 3.3)	2.8<br>(2.3, 3.5)	0.04<br>(-0.02, 0.09)
Sweden	693.1<br>(560.1, 849.3)	930.9<br>(755.8, 1152.7)	0.34<br>(0.28, 0.40)	6.1<br>(5.0, 7.4)	6.3<br>(5.2, 7.7)	0.04<br>(0.00, 0.08)
Switzerland	590.6<br>(476.7, 727.9)	932.7<br>(761.5, 1150.9)	0.58<br>(0.49, 0.67)	6.8<br>(5.5, 8.3)	7.1<br>(5.8, 8.7)	0.05<br>(0.00, 0.11)

United Kingdom	1568.6<br>(1261.7, 1949.7)	2037.8<br>(1632.8, 2536.7)	0.30<br>(0.27, 0.33)	2.1<br>(1.7, 2.6)	2.1<br>(1.7, 2.6)	-0.01<br>(-0.02, 0.00)
Western Sub-Saharan Africa	3111.8<br>(2565.5, 3828.7)	9318.5<br>(7635.3, 11570.1)	1.99<br>(1.89, 2.11)	2.4<br>(2.0, 3.0)	3.0<br>(2.4, 3.6)	0.23<br>(0.19, 0.27)
Benin	79.7<br>(65.5, 98.4)	215.7<br>(176.3, 267.5)	1.71<br>(1.53, 1.92)	2.6<br>(2.1, 3.3)	2.5<br>(2.0, 3.1)	-0.04<br>(-0.11, 0.03)
Burkina Faso	130.8<br>(106.7, 160.6)	303.4<br>(249.6, 371.1)	1.32<br>(1.16, 1.50)	2.1<br>(1.7, 2.6)	2.1<br>(1.7, 2.6)	-0.02<br>(-0.09, 0.06)
Cabo Verde	5.5<br>(4.6, 6.8)	13.7<br>(11.1, 16.9)	1.47<br>(1.28, 1.71)	2.2<br>(1.8, 2.7)	2.6<br>(2.1, 3.2)	0.20<br>(0.13, 0.29)
Cameroon	163.7<br>(135.0, 200.2)	491.6<br>(402.6, 606.6)	2.00<br>(1.82, 2.23)	2.4<br>(2.0, 2.9)	2.3<br>(1.9, 2.9)	-0.03<br>(-0.09, 0.04)
Chad	69.5<br>(56.8, 85.1)	214.5<br>(173.6, 268.4)	2.08<br>(1.84, 2.39)	1.8<br>(1.4, 2.2)	2.1<br>(1.7, 2.6)	0.19<br>(0.10, 0.27)
Côte d'Ivoire	173.0<br>(140.9, 213.6)	377.0<br>(304.8, 469.3)	1.18<br>(1.03, 1.33)	2.3<br>(1.9, 2.8)	2.0<br>(1.6, 2.5)	-0.13<br>(-0.19, -0.07)
Gambia	13.4<br>(11.0, 16.9)	32.7<br>(26.8, 40.5)	1.44<br>(1.31, 1.59)	2.2<br>(1.8, 2.7)	2.0<br>(1.7, 2.5)	-0.08<br>(-0.13, -0.02)
Ghana	339.8<br>(278.7, 412.7)	807.1<br>(666.4, 999.7)	1.38<br>(1.22, 1.54)	3.5<br>(2.8, 4.3)	3.3<br>(2.7, 4.0)	-0.06<br>(-0.12, 0.00)
Guinea	87.5<br>(71.3, 107.0)	194.9<br>(159.5, 238.5)	1.23<br>(1.06, 1.41)	2.1<br>(1.7, 2.5)	2.2<br>(1.8, 2.7)	0.08<br>(0.00, 0.17)
Guinea-Bissau	11.4<br>(9.2, 14.0)	26.8<br>(21.9, 33.6)	1.36<br>(1.20, 1.52)	1.8<br>(1.5, 2.2)	2.1<br>(1.7, 2.5)	0.14<br>(0.07, 0.23)
Liberia	50.9<br>(41.5, 63.0)	82.4<br>(67.5, 103.2)	0.62<br>(0.51, 0.75)	3.1<br>(2.5, 3.8)	2.2<br>(1.8, 2.7)	-0.28<br>(-0.33, -0.24)
Mali	122.6<br>(100.8, 149.6)	313.6<br>(256.7, 388.1)	1.56<br>(1.38, 1.75)	2.2<br>(1.8, 2.6)	2.2<br>(1.8, 2.6)	0.01<br>(-0.06, 0.08)
Mauritania	24.8<br>(20.2, 30.6)	75.4<br>(61.2, 93.2)	2.03<br>(1.79, 2.30)	1.8<br>(1.4, 2.2)	2.4<br>(2.0, 3.0)	0.39<br>(0.28, 0.51)
Niger	89.9<br>(73.1, 111.9)	301.7<br>(247.1, 374.7)	2.36<br>(2.09, 2.57)	1.9<br>(1.5, 2.3)	2.2<br>(1.7, 2.7)	0.14<br>(0.05, 0.22)

Nigeria	1482.6<br>(1218.6, 1826.8)	5343.6<br>(4375.7, 6636.1)	2.60<br>(2.44, 2.78)	2.4<br>(1.9, 2.9)	3.7<br>(3.0, 4.5)	0.53<br>(0.48, 0.60)
Sao Tome and Principe	1.7<br>(1.4, 2.0)	4.0<br>(3.3, 5.0)	1.42<br>(1.26, 1.60)	2.0<br>(1.6, 2.5)	2.4<br>(2.0, 3.0)	0.21<br>(0.14, 0.29)
Senegal	150.4<br>(123.8, 185.5)	268.3<br>(221.4, 331.6)	0.78<br>(0.67, 0.91)	3.1<br>(2.5, 3.7)	2.4<br>(1.9, 2.9)	-0.22<br>(-0.27, -0.17)
Sierra Leone	57.9<br>(46.9, 71.0)	131.0<br>(106.1, 162.8)	1.26<br>(1.11, 1.43)	2.0<br>(1.6, 2.4)	2.2<br>(1.8, 2.7)	0.09<br>(0.01, 0.18)
Togo	56.5<br>(45.7, 68.9)	121.1<br>(98.2, 150.4)	1.14<br>(0.98, 1.31)	2.6<br>(2.1, 3.1)	2.0<br>(1.7, 2.5)	-0.21<br>(-0.25, -0.16)

Table S6. Number of DALYs, death, and prevalence of pulmonary arterial hypertension in 1990 and 2021, and the percent change from 1990 to 2021, across different age groups

	DALYs			Death			Prevalence		
	Number in 1990	Number in 2021	Percent change	Number in 1990	Number in 2021	Percent change	Number in 1990	Number in 2021	Percent change
All ages	687419.3 (535240.8, 813086.3)	642104.3 (552272.7, 728993.2)	-0.07 (-0.27, 0.18)	14842.5 (12369.9, 17484.9)	22020.5 (18239.2, 25351.6)	1.42 (1.00, 1.74)	105703.3 (86381.4, 130334.3)	191808.2 (155356.9, 235787.1)	0.81 (0.73, 0.89)
> 70	72691.7 (57581.8, 88745.8)	169218.0 (130210.8, 199842.0)	1.33 (0.82, 1.87)	4920.8 (3858.3, 6007.3)	12544.3 (9683.9, 14828.9)	1.55 (0.99, 2.16)	15653.5 (11388.4, 21534.3)	36819.9 (26886.9, 50404.1)	1.35 (1.31, 1.40)
50-69	97977.0 (79225.9, 119253.5)	147104.2 (120476.9, 170135.1)	0.50 (0.23, 0.82)	3164.1 (2533.4, 3854.0)	4736.8 (3790.4, 5546.5)	0.50 (0.22, 0.83)	33093.6 (23553.0, 46264.9)	69388.0 (49465.2, 97042.1)	1.10 (1.07, 1.12)
15-49	158009.4 (125342.1, 196290.2)	174683.9 (149541.1, 204259.9)	0.11 (-0.04, 0.29)	2708.3 (2138.3, 3371.2)	3024.8 (2589.2, 3530.4)	0.12 (-0.03, 0.31)	49341.5 (37691.6, 65709.2)	76626.8 (57920.3, 101894.1)	0.55 (0.48, 0.62)
0-14	358741.2 (209032.7, 498948.5)	151098.1 (119751.9, 185128.4)	-0.58 (-0.69, -0.39)	4049.3 (2365.9, 5629.4)	1714.6 (1357.6, 2102.3)	-0.58 (-0.69, -0.39)	7614.7 (5374.5, 10085.4)	8973.6 (6312.6, 11961.6)	0.18 (0.16, 0.20)

DALYs: Disability adjusted life years.

Table S7. Rate of DALYs, death, and prevalence of pulmonary arterial hypertension in 1990 and 2021, and the percent change from 1990 to 2021, across different age groups

	DALYs			Death			Prevalence		
	Rate in 1990	Rate in 2021	Percent change	Rate in 1990	Rate in 2021	Percent change	Rate in 1990	Rate in 2021	Percent change
All ages	12.9 (10.0, 15.2)	8.1 (7.0, 9.2)	-0.37 (-0.51, -0.20)	0.3 (0.2, 0.3)	0.3 (0.2, 0.3)	0.00 (-0.18, 0.21)	2.0 (1.6, 2.4)	2.4 (2.0, 3.0)	0.23 (0.17, 0.28)
> 70	36.0 (28.5, 43.9)	34.2 (26.3, 40.4)	-0.05 (-0.26, 0.17)	2.4 (1.9, 3.0)	2.5 (2.0, 3.0)	0.04 (-0.19, 0.29)	7.7 (5.6, 10.7)	7.4 (5.4, 10.2)	-0.04 (-0.06, -0.02)
50-69	14.4 (11.6, 17.5)	10.2 (8.4, 11.8)	-0.29 (-0.42, -0.14)	0.5 (0.4, 0.6)	0.3 (0.3, 0.4)	-0.29 (-0.42, -0.13)	4.9 (3.5, 6.8)	4.8 (3.4, 6.8)	0.00 (-0.02, 0.01)
15-49	5.8 (4.6, 7.2)	4.4 (3.8, 5.2)	-0.24 (-0.34, -0.11)	0.1 (0.1, 0.1)	0.1 (0.1, 0.1)	-0.23 (-0.33, -0.10)	1.8 (1.4, 2.4)	1.9 (1.5, 2.6)	0.07 (0.02, 0.11)
0-14	20.6 (12.0, 28.7)	7.5 (6.0, 9.2)	-0.64 (-0.73, -0.47)	0.2 (0.1, 0.3)	0.1 (0.1, 0.1)	-0.63 (-0.73, -0.47)	0.4 (0.3, 0.6)	0.4 (0.3, 0.6)	0.02 (0.00, 0.03)

DALYs: Disability adjusted life years.

Table S8. Global number of DALYs, death, and prevalence of pediatric pulmonary arterial hypertension in 1990 and 2021, and the percent change from 1990 to 2021, across different age groups

Age group	DALYs			Death			Prevalence		
	Number in 1990	Number in 2021	Percent Change	Number in 1990	Number in 2021	Percent change	Number in 1990	Number in 2021	Percent change
15-19 years	22070.3 (17289.8, 28512.2)	19273.1 (15485.6, 24753.1)	-0.13 (-0.25, 0.02)	298.0 (231.8, 387.5)	258.4 (206.9, 334.3)	-0.13 (-0.26, 0.02)	4869.3 (2877.7, 7375.4)	5735.7 (3365.9, 8767.6)	0.18 (0.16, 0.20)
10-14 years	14885.4 (11379.9, 18570.0)	12328.6 (9823.2, 15345.2)	-0.17 (-0.32, 0.01)	187.7 (141.4, 236.1)	153.9 (121.8, 193.8)	-0.18 (-0.33, 0.00)	3535.9 (2364.5, 4992.7)	4317.7 (2876.1, 6154.2)	0.22 (0.20, 0.24)
5-9 years	20588.4 (14825.8, 27100.6)	12812.8 (10347.5, 15867.3)	-0.38 (-0.54, -0.15)	245.5 (176.0, 323.2)	151.2 (121.5, 188.3)	-0.38 (-0.55, -0.16)	2675.0 (1930.3, 3507.3)	3133.2 (2248.5, 4120.2)	0.17 (0.16, 0.18)
2-4 years	27861.9 (12977.3, 60507.7)	13528.0 (7336.0, 23842.3)	-0.51 (-0.65, -0.27)	319.8 (148.3, 695.3)	154.9 (83.2, 274.1)	-0.52 (-0.65, -0.28)	1094.2 (781.4, 1483.7)	1202.1 (858.0, 1646.7)	0.10 (0.09, 0.11)
12-23 months	35308.3 (20231.7, 55903.3)	15113.9 (11528.6, 19462.9)	-0.57 (-0.72, -0.36)	398.4 (228.2, 630.9)	170.4 (129.9, 219.5)	-0.57 (-0.72, -0.36)	218.4 (149.2, 304.0)	227.8 (154.9, 316.7)	0.04 (0.03, 0.05)
< 12 months	260097.2 (151032.3, 353095.7)	97314.9 (74076.5, 122690.2)	-0.63 (-0.72, -0.45)	2898.0 (1681.9, 3936.3)	1084.1 (825.1, 1367.0)	-0.63 (-0.72, -0.45)	91.2 (60.3, 129.5)	92.8 (61.9, 131.7)	0.02 (0.01, 0.03)

DALYs: Disability adjusted life years.

Table S9. Global rate of DALYs, death, and prevalence of pediatric pulmonary arterial hypertension in 1990 and 2021, and the percent change from 1990 to 2021, across different age groups

Age group	DALYs			Death			Prevalence		
	Rate in 1990	Rate in 2021	Percent change	Rate in 1990	Rate in 2021	Percent change	Rate in 1990	Rate in 2021	Percent change
**15-19 years**	4.25 (3.33, 5.49)	3.09 (2.48, 3.97)	-0.27 (-0.34, -0.15)	0.06 (0.04, 0.07)	0.04 (0.03, 0.05)	-0.28 (-0.38, -0.15)	0.94 (0.55, 1.42)	0.92 (0.54, 1.41)	-0.02 (-0.04, -0.01)
**10-14 years**	2.78 (2.12, 3.47)	1.85 (1.47, 2.30)	-0.33 (-0.45, -0.19)	0.04 (0.03, 0.04)	0.02 (0.02, 0.03)	-0.34 (-0.46, -0.19)	0.66 (0.44, 0.93)	0.65 (0.43, 0.92)	-0.02 (-0.03, -0.01)
**5-9 years**	3.53 (2.54, 4.64)	1.86 (1.51, 2.31)	-0.47 (-0.61, -0.28)	0.04 (0.03, 0.06)	0.02 (0.02, 0.03)	-0.48 (-0.62, -0.28)	0.46 (0.33, 0.60)	0.46 (0.33, 0.60)	-0.01 (-0.02, 0.00)
**2-4 years**	7.58 (3.53, 16.46)	3.36 (1.82, 5.92)	-0.56 (-0.68, -0.34)	0.09 (0.04, 0.19)	0.04 (0.02, 0.07)	-0.56 (-0.68, -0.34)	0.30 (0.21, 0.40)	0.30 (0.21, 0.41)	0.00 (-0.01, 0.01)
**12-23 months**	28.33 (16.23, 44.86)	11.77 (8.98, 15.16)	-0.58 (-0.73, -0.37)	0.32 (0.18, 0.51)	0.13 (0.10, 0.17)	-0.58 (-0.73, -0.38)	0.18 (0.12, 0.24)	0.18 (0.12, 0.25)	0.01 (0.00, 0.02)
**< 12 months**	203.60 (118.23, 276.40)	76.81 (58.47, 96.84)	-0.62 (-0.72, -0.44)	2.27 (1.32, 3.08)	0.86 (0.65, 1.08)	-0.62 (-0.72, -0.44)	0.07 (0.05, 0.10)	0.07 (0.05, 0.10)	0.03 (0.02, 0.04)

DALYs: Disability adjusted life years.

Table S10. Projections of global burdens in pulmonary arterial hypertension through 2050 across different sex population

Indicator	Year	Number			Age-standardized rate		
		Both	Female	Male	Both	Female	Male
DALYs	2025	622578.0 (560748.3, 684407.7)	341188.9 (295467.6, 386910.2)	279927.4 (247108.5, 312746.4)	7.51 (6.76, 8.26)	7.85 (6.79, 8.92)	7.12 (6.28, 7.96)
	2030	604806.8 (517218.6, 692395.0)	337134.7 (270418.5, 403850.9)	266969.8 (221716.9, 312222.7)	6.72 (5.75, 7.70)	7.14 (5.73, 8.55)	6.27 (5.21, 7.33)
	2035	586675.3 (502526.6, 670824.0)	332612.2 (266946.3,398278.2)	254264.6 (211743.8, 296785.4)	6.03 (5.17, 6.89)	6.49 (5.22, 7.77)	5.53 (4.61, 6.45)
	2040	567125.6 (466020.7, 668230.5)	326978.7 (246291.5, 407665.9)	241391.0 (191340.7, 291441.2)	5.41 (4.46, 6.36)	5.91 (4.47, 7.36)	4.89 (3.88, 5.89)
	2045	544892.6 (448243.1, 641542.0)	319389.5 (240569.4, 398209.5)	227947.9 (181019.7, 274876.1)	4.86 (4.02, 5.70)	5.39 (4.08, 6.70)	4.33 (3.45, 5.20)
	2050	519398.8 (412506.0, 626291.6)	309349.9 (220303.7, 398396.1)	213738.4 (162790.2, 264686.7)	4.38 (3.50, 5.25)	4.92 (3.53, 6.30)	3.84 (2.94, 4.73)
Death	2025	24518.7 (22771.5, 26266.0)	13808.6 (12601.4, 15015.9)	10545.6 (9646.7 11444.5)	0.28 (0.26, 0.29)	0.28 (0.26, 0.31)	0.26 (0.24, 0.29)
	2030	26800.3 (24441.7, 29158.9)	15235.5 (13546.2, 16924.9)	11371.3 (10175.2, 12567.5)	0.26 (0.24, 0.29)	0.27 (0.24, 0.30)	0.25 (0.22, 0.27)
	2035	29321.5 (26843.3,31799.8)	16847.8 (15015.6, 18679.9)	12248.9 (11008.7, 13489.0)	0.25 (0.23, 0.27)	0.27 (0.24, 0.29)	0.24 (0.21, 0.26)
	2040	31920.2 (28762.3, 35078.0)	18555.4 (16155.9, 20954.9)	13105.1 (11546.3, 14663.9)	0.24 (0.22, 0.27)	0.26 (0.22, 0.29)	0.22 (0.20, 0.25)
	2045	34395.2 (30979.2, 37811.3)	20222.8 (17566.6, 22879.0)	13881.5 (12216.7, 15546.2)	0.23 (0.21, 0.26)	0.25 (0.22, 0.28)	0.21 (0.19, 0.24)

	**2050**	36594.8<br>(32487.7, 40701.9)	21740.5<br>18484.5, 24996.4)	14536.7<br>(12559.3, 16514.1)	0.22<br>(0.20, 0.25)	0.24<br>(0.20, 0.28)	0.20<br>(0.17, 0.23)
**Prevalence**	**2025**	216899.2<br>(182026.5, 251771.9)	133496.5<br>(114053.2, 152939.7)	83219.0<br>(69693.3, 96744.7)	2.39<br>(2.00, 2.78)	2.87<br>(2.45, 3.29)	1.88<br>(1.58, 2.19)
	**2030**	235573.8<br>(190373.2, 280774.4)	144739.6<br>(119581.2, 169897.9)	90744.1<br>(73229.2, 108259.0)	2.40<br>(1.93, 2.86)	2.87<br>(2.36, 3.38)	1.90<br>(1.53, 2.27)
	**2035**	253672.5<br>(211157.7, 296187.2)	155543.1<br>(131900.3, 179185.8)	98121.6<br>(81633.0, 114610.3)	2.40<br>(1.99, 2.81)	2.87<br>(2.43, 3.32)	1.92<br>(1.59, 2.24)
	**2040**	270724.9<br>(218892.6, 322557.1)	165583.8<br>(136783.7, 194383.9)	105200.4<br>(85055.0, 125345.8)	2.41<br>(1.94, 2.88)	2.87<br>(2.36, 3.39)	1.93<br>(1.55, 2.31)
	**2045**	286412.2<br>(235006.7, 337817.7)	174659.5<br>(146128.6, 203190.5)	111864.3<br>(91822.6, 131906.1)	2.41<br>(1.97, 2.86)	2.87<br>(2.39, 3.36)	1.95<br>(1.59, 2.30)
	**2050**	300484.7<br>(240568.8, 360400.6)	182626.5<br>(149420.8, 215832.1)	118009.5<br>(94560.0, 141459.00)	2.42<br>(1.92, 2.92)	2.87<br>(2.33, 3.42)	1.96<br>(1.56, 2.37)

DALYs: Disability adjusted life years.
